# Toward Explicit Solvation for Simulations of Electrocatalytic Reactions: AIMD for p*K*_a_ and Redox Potentials of Transition Metal Compounds and Catalyst Models

**DOI:** 10.1021/acs.jpca.4c06898

**Published:** 2025-02-03

**Authors:** Gustavo T. Feliciano, Alexander A. Auer

**Affiliations:** Department of Molecular Theory and Spectroscopy, Max-Planck-Institut für Kohlenforschung, 45470 Mülheim an der Ruhr, Germany

## Abstract

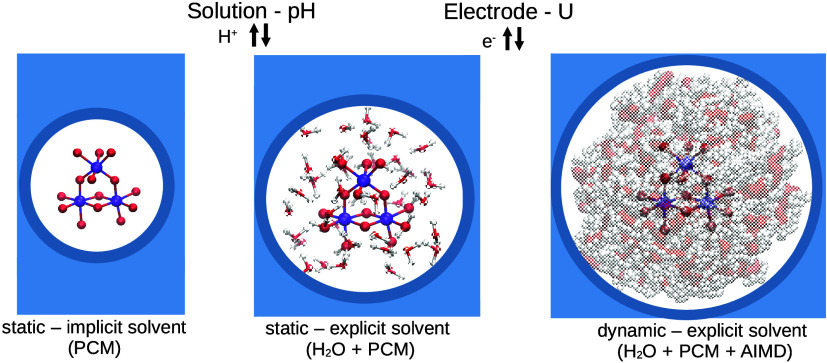

In this work, we study the possibility to extend electronic structure simulations for electrocatalysis by explicit solvation models. In previous work, we proposed a simulation scheme that explicitly includes the effects of pH and electrochemical potential in density functional theory (DFT) simulations with implicit solvation. Based on calculations of protonation and oxidation reactions, the pH and electrochemical potential can be included given appropriate reference values. In this work, we compute the p*K*_a_ values and oxidation potentials for a series of transition metal aquo complexes and compare the results including implicit, explicit static and explicit dynamic (AIMD) models for the aqueous solvent and compare vs experimental p*K*_a_ and redox potential data. This allows the construction of a p*K*_a_/redox potential scale that can in principle be extrapolated to the simulation of other transition metal-based materials. An explicit dynamic solvent model is then proposed and applied to a model system for iridium oxide-based catalysts for the oxygen evolution reaction. We outline the advantages and disadvantages of the different approaches and demonstrate that, at the expense of a larger computational effort, the microsolvation environment of a given model can be described in a robust way using a limited amount of solvent molecules and AIMD. Especially for reactions in which water is solvent and reactant like the oxygen evolution reaction (OER) or oxygen reduction reaction (ORR), this model provides a more detailed and complete description that can be exploited in mechanistic studies.

## 1 Introduction

Quantum chemical methods have become powerful tools in many areas of chemistry. The main reasons for this are (i) the possibility to compute a broad range of molecular properties, (ii) the advent of approximations that allow to apply computations to complex systems within robust and hierarchical approximations, and (iii) the development of computer codes that are available to a broader community. Approaches like analytical derivatives^1−3^ and response theory,^4−7^ approximations like linear scaling density functional theory (DFT) methods,^8−10^ and codes free for academic use like ORCA^11^ or open source codes like DALTON^12^ highlight these features and demonstrate how quantum chemistry has evolved powerful techniques in recent decades.

One of the most challenging problems for theory can be found in the field of heterogeneous catalysis: electrocatalytic reactions, which are essential in many renewable energy economy scenarios, exhibit a whole range of complex phenomena. For key technologies like fuel cells and electrolyzers, the oxygen reduction reaction (ORR) and the oxygen evolution reaction (OER) require a description of the three-phase boundary including exchange of electrons with the electrode and protons with the solvent—water—which is also reactant in this case. Here, DFT calculations are frequently employed to study reaction mechanisms or possible intermediates. For a more complete description, a computational framework including the proton exchange processes and the proton chemical potential in solution (pH) as well as an inclusion of the electrode potential in the simulation is essential in order to accurately compute reaction paths and assign rate-limiting steps.

Several methods have been proposed in order to simulate electrocatalytic systems under constant potential conditions. Lozovoi and Alavi presented an efficient scheme for grand-canonical periodic boundary simulations of constant potential processes.^13^ Pasquarello and co-workers proposed a method where the Fermi level of the system is kept fixed introducing a specific equation of motion for it, as a function of the total charge.^14,15^ Another scheme using ab initio molecular dynamics (AIMD) and a fictitious description of the electrode potential over redox species by evaluating oxidation free energies with enhanced sampling methods was outlined by Blumberger and Sprik.^16^ In order to increase the efficiency of AIMD simulations in computing oxidation (deprotonation) free energies, machine learning potentials can also be employed. Initial full AIMD trajectories are used to ”train” simpler potentials, which are employed to accelerate statistical convergence of enhanced sampling AIMD-based methods, such as thermodynamic integration.^17−19^ In one of the arguably most complete simulations, AIMD has been performed with the explicit inclusion of a doped counterelectrode as reference, enforcing the constant potential condition not only in energy, but also in the water/electrode interface structure description.^20^

The inclusion of the pH effect is even more challenging. Rossmeisl et al. addressed the problem at the metal water interface, employing a generalized computational hydrogen electrode approach, and obtained properties such as differential capacitance and potential of zero charge as a function of the pH.^21^ Another seminal work deals with specific hydration effects at the potential of zero charge in transition metal electrodes in contact with water: Le et al. demonstrate how the deprotonation and oxidation events are related at the PZC, and show that the use of implicit energy references for proton and electron withdrawal, together with structural sampling of the liquid phase, can lead to accurate and efficient models for solid–liquid interfaces under constant potential conditions.^22^ In principle, it is possible to apply that strategy to determine the surface structure of oxidic materials in solution. However, there are multiple possibilities of assigning protons to the titratable oxygen atoms. It was previously demonstrated how this problem can be addressed by evaluating free energy changes in static calculations with implicit solvation.^23,24^ In this approach, the charge and protonation state of a model system are explicitly changed, and with a proper reference value the most stable structures at given pH/potential values can be identified. The only limitation of this model is the neglect of specific microsolvation effects (water distribution upon nucleophilic attack, dynamic proton transfer pathways, etc.) that are present for OER electrocatalysis in transition metal oxides. The method was successfully applied to describe the evolution of the pH/potential dependent surface structure and the OER reaction profile in iridium oxide-based materials, as well as the effects of iron incorporation on the OER efficiency in cobalt oxide catalysts.

In this work we study the possibility to extend the computational models for the description of electrocatalytic reactions in aqueous media using explicit solvent models. On the one hand, we aim at preserving the advantages of a method that explicitly includes the exchange of electrons and protons with a bath, on the other hand we want to go beyond implicit solvent models maintaining a good compromise between efficiency and accuracy. We consider a simple model where explicit solvent molecules are included in a given radius from the system of interest and the bulk/charge screening effects are further described by a polarizable continuum approach. The pH/potential effects are still taken into account by considering proton/electron exchange with an implicit reservoir or bath. In order to sample solvent configurations we extend this approach by including averages over trajectories from AIMD at the DFT level of theory.

After introduction of the methods and models applied, the following sections will first focus on the application of an explicit solvent model plus AIMD for several transition metal aquo complexes for which experimental redox potentials and p*K*_a_ values are available. These not only serve as benchmark examples for the methodology, but are also required as reference values for further studies on catalyst models. After discussing pros and cons and highlighting some of the pitfalls of the approach, we will apply the scheme to an IrO_*x*_ nanoparticle, which is a common model for studying the most potent OER catalyst in acidic conditions.

## 2 Methods

The aim of this work is to study different protocols for the description of electrocatalytic reactions which include as many of the complex influences that are vital for electrochemistry as possible. Among a multitude of factors, there are two key elements common for electrocatalytic processes in aqueous solvents: the presence of the electrode potential and its effect on the interface structure, and the description of the aqueous solvent environment around the catalyst that includes the effects of pH and the possible role of water as reactant.

**Figure 1:**
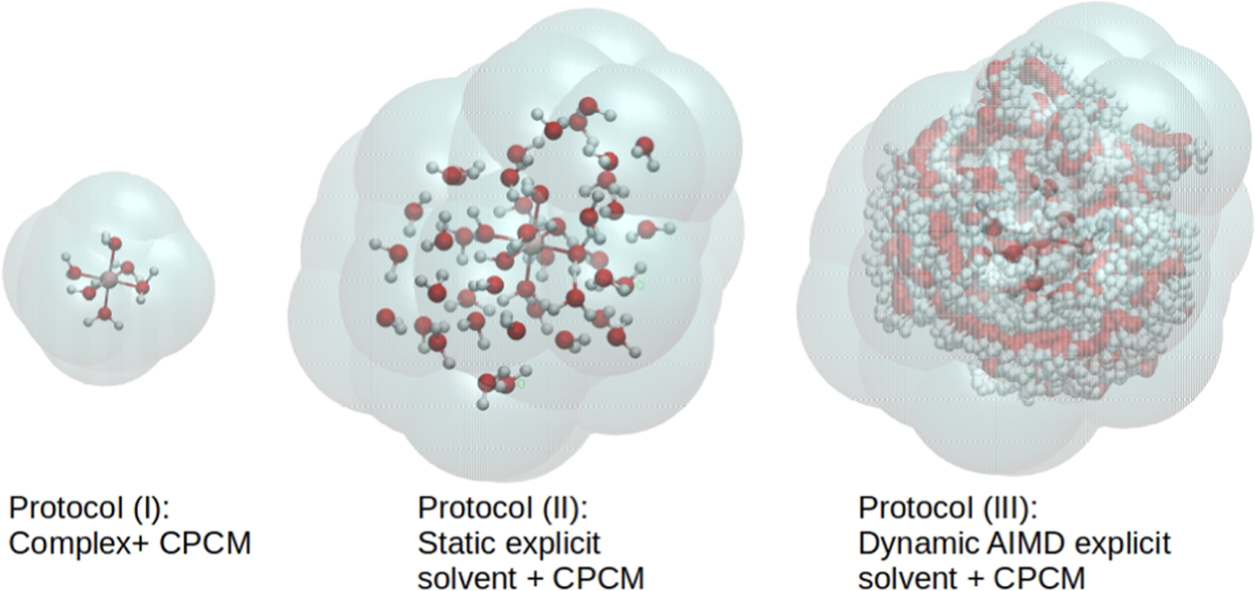
Illustration of the three protocols for solvation considered in this work, for the hexaaqua ion system.

For the description of the solvent, the best trade-off between accuracy and computational effort is not obvious. In this study, three protocols are compared (illustrated in Figure 1): (i) the standard implicit solvation protocol, which consists of geometry optimization of the system including implicit solvation, in this case using the conductor-like polarizable continuum model (CPCM).^25^ (ii) The static explicit solvation protocol: geometry optimization performed with explicit solvation consisting of a cluster of 8 Å radius of water molecules around the considered system (roughly 3 hydration shells) embedded in CPCM. (iii) The dynamic explicit protocol: AIMD applied to generate trajectories in the NVT ensemble based on the structures from (ii). Note that hybrid implicit/explicit solvation schemes like this have recently been applied successfully in the computation of solvation free energies for charged species, with some advantages in handling longe-range electrostatics and configurational sampling of the aqueous environment.^26−31^

The polarizable continuum solvation is obviously the simplest choice: the environment shields the excess charge like a conductor. However, liquid water is a structured medium, and each molecule has its own charge distribution. An explicit representation of solvent molecules is a more complete model, which considers both short-range charge screening properties and water structural rearrangement. The number of degrees of freedom is very large in this case, and a static explicit solvation approach carries an intrinsic arbitrariness of the chosen configuration. AIMD constitutes an efficient way of generating many configurations for a statistical approach to water reorganization and should yield an average over the complex events in the hydrogen bonding network of the solvent. A comparison of these different levels of approximation to solvation should allow to disentangle effects like electrostatic contributions and configurational sampling. On the other hand, energy fluctuation in AIMD usually demands long trajectories for statistically converged averages. This comprises an intensive computational effort: tens of thousands of required timesteps (energy and gradient) in the AIMD with a N^3^ scaling for electronic structure calculations (N being the number of electrons) must be considered.

As for the inclusion of pH and potential effects, the idea is to model the exchange of protons and electrons with a proton and an electron reservoir, while explicitly changing the protonation state and charge of the system in question. However, a proper energy reference must first be assigned to those reservoirs. We chose this reference to be hexaaqua transition metal complexes containing the Cu, Fe, Ni, Co and Ir metal ions. These compounds are structurally simple and well-defined, and experimental p*K*_a_ and oxidation potentials are available across a wide range of conditions, as shown in Table 1. Hence, these are a natural choice for benchmarking methods and approximations as well as referencing the computed energies for the proton and electron reservoirs to relative p*K*_a_ and potential values.

**Table 1:** Hexaaqua Complexes Considered for Benchmarking p*K*_a_ and Oxidation Potential Values with Respect to Experimental Values along with the Corresponding Redox and Acid–Base Pairs

acid/base pairs	p*K*_a(exp.)_
Ir(H_2_O)_6_^3+^/Ir(H_2_O)_5_(OH)^2+^	4.4^32^
Ir(H_2_O)_5_(OH)^2+^/Ir(H_2_O)_4_(OH)_2_^+^	5.3^32^
Fe(H_2_O)_6_^3+^/Fe(H_2_O)_5_(OH)^2+^	2.2^33^
Fe(H_2_O)_6_^2+^/Fe(H_2_O)_5_(OH)^+^	9.5^33^
Co(H_2_O)_6_^3+^/Co(H_2_O)_5_(OH)^2+^	2.9^33^
Co(H_2_O)_6_^2+^/Co(H_2_O)_5_(OH)^+^	9.7^33^
Cu(H_2_O)_6_^2+^/Cu(H_2_O)_5_(OH)^+^	7.4^33^
Ni(H_2_O)_6_^2+^/Ni(H_2_O)_5_(OH)^+^	9.9^33^

redox pairs	*U*_(exp.)_ (V)
Fe(H_2_O)_6_^2+^/Fe(H_2_O)_6_^3+^	0.77^34^
Co(H_2_O)_6_^2+^/Co(H_2_O)_6_^3+^	1.91^34^
Cu(H_2_O)_6_^+^/Cu(H_2_O)_6_^2+^	0.16^34^
Cu(H_2_O)_6_^2+^/Cu(H_2_O)_6_^3+^	2.40^34^^,^^a^
Ni(H_2_O)_6_^2+^/Ni(H_2_O)_6_^3+^	2.30^34^^,^^a^

a Experimental values are only estimated for these species.

For each chosen hexaaqua complex, we compute the energy of a given species and its oxidized form, and we define the difference as the oxidation free energy (without the removed electron energy reference). Analogously, we compute the deprotonation free energy for a given species as the free energy difference between the species and its deprotonated form (without the removed proton energy reference). If well-defined reference energies can be obtained, a linear relation between the energy differences and the corresponding redox potential, or p*K*_a_, respectively, should be obtained.

When a linear regression is applied for the computed deprotonation (oxidation) energies and the corresponding experimental p*K*_a_ (redox potential) values, the most adequate reference energy for the whole set can be extracted from the linear fit parameters, namely, the *y*-intersect, the slope and an *R*^2^ value. The slope contains the information whether relative energies are quantitatively reproduced, and relates to the description of underlying physics of the redox and protonation processes, while the *R*^2^ value provides a measure of the robustness of the fit and the transferability of the results. Hence, we will focus our assessment of the quality of the different approximations mostly on the slope and *R*^2^ values of the fits in the following.

The intention is to describe p*K*_a_ and potentials with an accuracy of 2–3 pH units and 0.1–0.2 V, respectively. This accuracy allows to distinguish several pH and potential regimes and to compute intermediates and reaction paths in acidic or alkaline solution and in oxidizing or reducing environments.

For the calculations applying protocol (ii) and (iii), the initial configuration with the explicit water molecules is generated using classical molecular dynamics. The complex is inserted in a periodic water box of 15 Å × 15 Å × 15 Å, using the force field engine GROMACS.^35^ The SPC force field^36^ was used for description of water, and the AMBER set of parameters for multivalent ions^37^ for the central ion. Soft restraints over the 6 direct aquo ligands have been applied to ensure a proper starting geometry. After 5 ns of molecular dynamics performed at 1 atm and 300 K a configuration is selected, resulting in an approximately spherical cluster with 8 Å diameter and 133 atoms in total (central ion and 44 water molecules as depicted in Figure 1). In protocol (ii), the snapshot extracted from classical MD is used as a starting point. The initial water structure is chosen to be exactly the same for all hexaaqua ions considered, and a simple geometry optimization is performed for all species. Deprotonation and oxidation energies are extracted directly for the optimized structures. Note that the obtained structures need to be checked to correspond to the expected protonation state.

Due to the symmetry of the hexaaqua ion charge distribution, geometry optimizations are expected to capture the solvent response in a uniform way, even with one particular fixed arrangement for the water molecules representing the solvent. Therefore, redox properties are expected to be still well described with a single water configuration, as long as the size and number of explicit molecules is kept constant. On the other hand, deprotonation energies are highly dependent on the explicit position of the protons and the hydrogen bonding network. Thus, the static explicit solvent model (ii) is not expected to have the best performance for p*K*_a_ computations.

For protocol (iii) AIMD simulations are performed using the Born–Oppenheimer approximation (BOMD) for 10 ps for each considered species. A time step of 0.5 fs is used for the integration of the equations of motion through the Velocity Verlet algorithm. The Nose-Hoover algorithm is used to keep the temperature constant at 300 K.^38,39^ The system is initially equilibrated for 2 ps with a coupling constant of 20 fs for the thermostat, which is changed to 100 fs in the production phase, from 2 to 10 ps. The oxidation and deprotonation free energies are computed from energies of all frames of the AIMD trajectories of corresponding species, averaged over the trajectory production phase, the initial 2 ps equilibration section is disregarded.

All electronic structure calculations have been performed using a development version of the ORCA 5.0 program package.^11,40^ Spin unrestricted DFT was employed in conjunction with the def2-SVP basis set and Def2/J auxiliary basis, with the resolution of identity approximation.^41,42^ Two different density functional protocols are compared in the following: the first one being the standard generalized gradient functional Perdew–Burke–Ernzerhoff (GGA-PBE)^43^ with the Grimme’s D3 dispersion correction and Becke-Johnson (BJ) damping.^44^ The second one is a protocol that has been recommended specifically for the simulation of water dynamics. It applies the meta-GGA r^2^SCAN^45^ without dispersion correction. r^2^SCAN has emerged as an interesting alternative for reproducing fundamental properties of liquid water with relatively low computational cost. It has been reported, however, that the D3 correction deteriorates the performance of the SCAN family of density functionals for water structural properties, predicting significantly shorter equilibrium distances and lower binding energies for hexameric water cluster systems, in comparison to coupled cluster based methods.^46,47^ The trust augmented radius Hessian (TRAH) optimizer, implemented in ORCA, was employed for accelerated SCF convergence.^48^ Geometry optimization was performed until energy changes are smaller than 10^–6^ E_*h*_ and maximum gradient is smaller than 3 × 10^–4^E_*h*_/Bohr, with no restraints in the water molecules. All molecular illustrations were rendered using the VMD 1.9.3 program.^49^

## 3 Redox Potentials/p*K*_a_ for Reference Systems in Electrocatalysis

### 3.1 Redox Potentials

**Figure 2:**
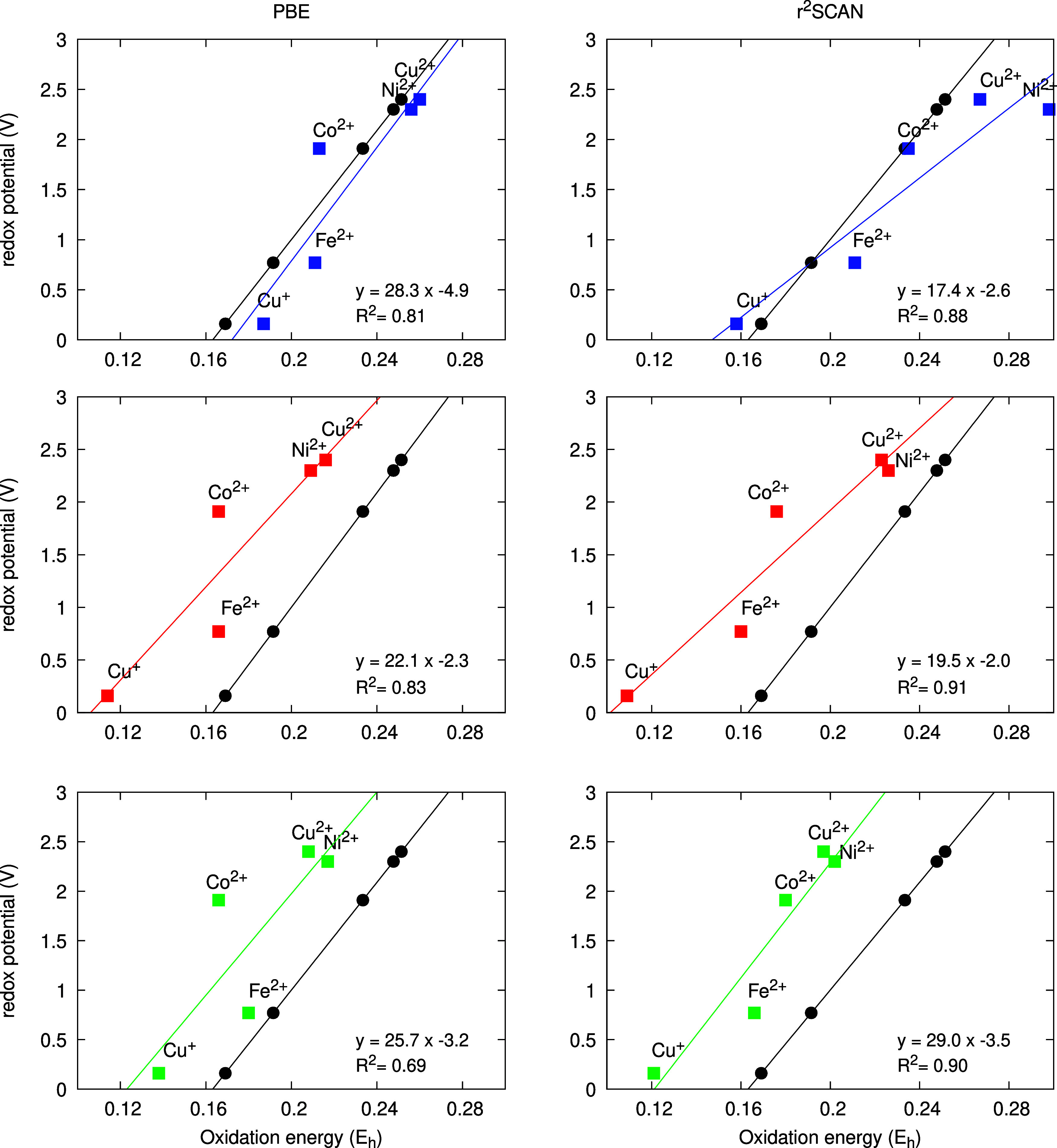
Oxidation energy of the transition metal aquo complexes, labeled according to the central metal ion as a function of the redox potential (top, blue for protocol (i), middle, red for (ii) and bottom, green for (iii)) obtained with the PBE-D3 (left) and r^2^SCAN (right) functionals, together with least-squares linear regression. Experimental data using experimental redox potentials and an experimental electron reference energy vs SHE as guide to the eye (in black).

The relation between the oxidation free energy of a given species and the one-electron oxidation potential can be written as , where the reference energy is the energy of the electron after removal from the complex, and Δ*G*_oxidation_ is the previously defined oxidation free energy. However, if one wants to remain within a quantum chemical model chemistry^50^ it is very challenging to compute the corresponding reference system energies. The expression above, however, denotes a linear relationship, where the reference system should be valid for any chemical species under consideration. Therefore, the available data from the chosen hexaaqua complexes in Table 1 are used to perform a linear regression analysis, relating the computed oxidation free energy to the experimental redox potential values. The computed ”redox scale” is compared to the ideal linear relationship obtained only from the experimental redox potential values and an experimental reference potential for the electron, e.g., the standard hydrogen electrode reference (SHE), and the *U*_SHE_ is taken as 4.44 V.^51^ The results of the redox potential scale benchmark of the selected hexaaqua ions for the three protocols are presented in Figure 2 for r^2^SCAN and PBE-D3. In the data shown in Figure 2, the oxidation free energies are expressed in Hartree, and in this case the ideal experimental value is 27.2 (V·E_*h*_^–1^) as the slope and −4.44 (V) as the *y*-intersect.

In protocol (i), the environment of the complexes is described via an implicit solvation approach, in which the average electrostatic effect of the solvent is included via a polarizable continuum with a given dielectric constant. The results show that the overall ordering of oxidation energies of the different species is reproduced correctly. The PBE-D3 + CPCM results exhibit remarkable agreement with the optimal behavior while for r^2^SCAN a smaller slope is obtained for the redox scale. The largest deviations are found for Co^2+^ and Fe^2+^. Note that this trend is observed for all protocols and both functionals. Hence, the Fe and Co species can be identified as cases where independent of the solvent model used, general functional deficiencies manifest themselves.

In protocol (ii) short-range solvent effects are described by explicit water molecules around the species in consideration. The electrostatic response now depends on one specific configuration chosen and how the optimized structure changes upon oxidation of the species. Nevertheless, the results indicate that a redox scale with fair quality can be obtained. However, while there is a slight improvement of the r^2^SCAN slope and *R*^2^ values, the PBE-D3 results actually deteriorate. The largest change is found for the *y*-intersect—as the electrostatic description of the environment charge has changed from implicit to explicit solvation, so does the electron energy reference. As it was previously outlined, a simple geometry optimization can capture solvent reorganization upon oxidation, due to the symmetry of the metal ion charge distribution. As a consequence, the explicit static solvation models predict redox potentials with reasonable accuracy.

For protocol (iii), the effect of averaging over the dynamics of the solvent reorganization under ambient temperature is taken into account. This should in principle present the most complete picture. As a result, the linear regression yields slopes which are much closer to the experimental scale than for protocol (ii). However, the statistical sampling does not systematically improve the scales in all aspects and while this model yields the best slope and *R*^2^ values for r^2^SCAN overall, the agreement for PBE-D3 is poor compared to the results from the implicit solvent model.

Overall, in combination with the correct functional, protocol (i) yields remarkable agreement with the optimal reference values and offers a cost-effective way to obtain redox potentials for the simulation of electrocatalytic processes. Explicit inclusion of solvent molecules with one single configuration as in protocol (ii) can yield results of acceptable accuracy as also demonstrated in previous studies.^52^ However, there is no clear advantage of this approach over implicit solvation for redox potentials in our case. Inclusion of the dynamics of an explicit solvent following protocol (iii) does improve on the static explicit protocol (ii) but is significantly more demanding computationally. However, note that for the r^2^SCAN functional there is a systematic improvement going from implicit to explicit to explicit dynamic solvent which implies, given the right functional, robust and accurate redox potentials can be obtained from AIMD averages.

### 3.2 p*K*_a_ Scale

For the p*K*_a_ (or pH, respectively) of a given species a similar expression as for the potential can be derived: Δ*G*_deprot_ – Δ*G*_ref_ = 2.303*RT* p*K*_a_, where Δ*G*_deprot_ is the deprotonation free energy. Δ*G*_ref_ stands for the reference free energy, which is the proton solvation free energy. A linear regression is applied, relating the computed deprotonation energies to the experimental p*K*_a_ values. The computational p*K*_a_ scale is also compared to the ideal experimental p*K*_a_ scale, constructed using −1104.5 kJ/mol^53^ for the proton solvation free energy. When the expression is rearranged as , the equation yields 460.14 (E_*h*_^–1^) for the slope and −193.62 for the *y*-intercept of the experimental p*K*_a_ scale at 298.15K.

**Figure 3:**
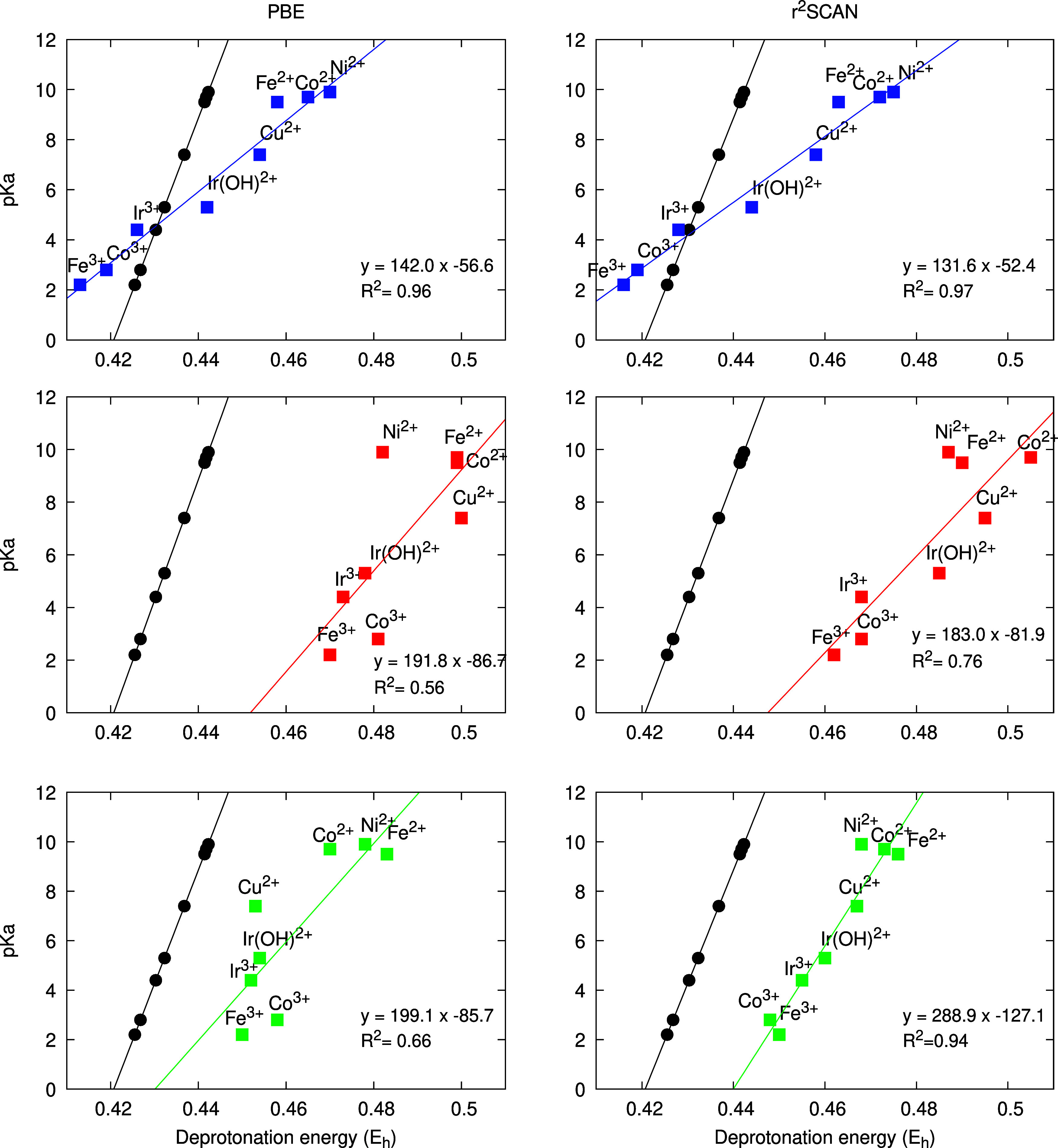
Deprotonation energy of the transition metal aquo complexes, labeled according to the central metal ion as a function of the p*K*_a_ (blue for protocol (i), red for (ii) and green for (iii)) obtained with the PBE-D3 (left) and r^2^SCAN (right) functionals with least-squares linear regression. Experimental data using experimental redox potentials and an experimental electron reference energy vs SHE is also supplied as a guide to the eye (in black).

The results for the p*K*_a_ scale from deprotonation free energies with the three protocols using PBE-D3 and r^2^SCAN are presented in Figure 3.

The results clearly show that the simplified description of the microsolvation environment with polarizable continuum methods has a different behavior for protonation or deprotonation than for oxidation and reduction. As protonation and deprotonation directly affects the local structure of the model, all p*K*_a_ scale slopes are significantly lower than the experimental p*K*_a_ scale. In contrast to the redox scale, however, the p*K*_a_ scales show very similar slopes and *y*-intercepts for both functionals and also exhibit significantly higher *R*^2^ values.

Protocol (ii) does not represent an improvement over protocol (i). On the contrary, using a single water structure to describe microsolvation reduces the quality of the p*K*_a_ scale results. This comes as no surprise, as deprotonation can result in many different configurations of the hydrogen bonding network, and a simple rearrangement of a given hydrogen bond can cause changes as high as 6 kcal/mol (approximately 4.5 pH units). Although static implicit models can overestimate the deprotonation energy differences, the simplification of the hydrogen bonding network description causes a similar dielectric response for all species, avoiding the sampling issue, and preserves the energy scale ordering.

Analysis of protocol (iii) shows that, for PBE-D3, the trends of the deprotonation energy for the most acidic and most alkaline compounds have improved, but Cu^2+^ and Co^3+^ exhibit large deviations from the linear scale. In the r^2^SCAN case, however, we see the most balanced performance among all previous calculations. The deprotonation energy ordering shows small deviations from the fitted linear scale, while the slope is closest to the experimental p*K*_a_ scale. One common trend in p*K*_a_ and redox scale for protocol (iii) is that PBE-D3-based scales are always poorer than the corresponding r^2^SCAN ones.

In summary, protocol (i) results in robust p*K*_a_ and redox scales for application in electrocatalysis despite its simplicity. It is also readily applicable to implicit solvation schemes with charge/protonation sampling for inclusion of pH and potential effects. The difficulty here lies in the generation of all relevant protonation/charge configurations for the electrocatalyst material to be studied, as in this case, pH and potential effects are considered by explicit charge and protonation changes in the initial structure. Note that this scheme is currently the state-of-the-art in simulation of electrocatalytic processes, and it was already successfully applied to many systems.^23,24^ The effort is significantly lower than for AIMD, but specific effects of microsolvation over the considered chemical reactions are neglected.

When monitoring the changes going from protocol (i) to (iii) for the redox and the p*K*_a_ scale, the r^2^SCAN results seem to improve significantly, while the PBE-D3 results deteriorate for the redox scale and only show slight improvement for the p*K*_a_ scale. This implies that in addition to the inherent behavior of the functional for energy differences and the effect of a dynamic explicit solvent, there is another important contribution—the description of the water microstructure and dynamics of the DFT functional. The next section includes a more detailed analysis of the AIMD simulations for the cases described above.

### 3.3 Structural Features from AIMD Simulations—Influence of the Water Structure over pH/Redox Properties

Previous studies have shown that the description of structural properties of water can be very sensitive to the employed density functional.^54−57^ GGA density functionals are known to not fully capture dispersion related phenomena. This is often counterbalanced by an empirical atom-centered potential (as it is in this study, with the PBE functional), but the overall effect is usually an overcompensation, in case of liquid water. As a result, the hydrogen bonding network is slightly overstructured. Furthermore, the thermal energy distribution in AIMD becomes more concentrated in vibrational contributions rather than translational ones. This can be seen, for example, when AIMD is used to describe the water self-diffusion coefficient, as it is considerably smaller when using GGA functionals.^57^

**Figure 4:**
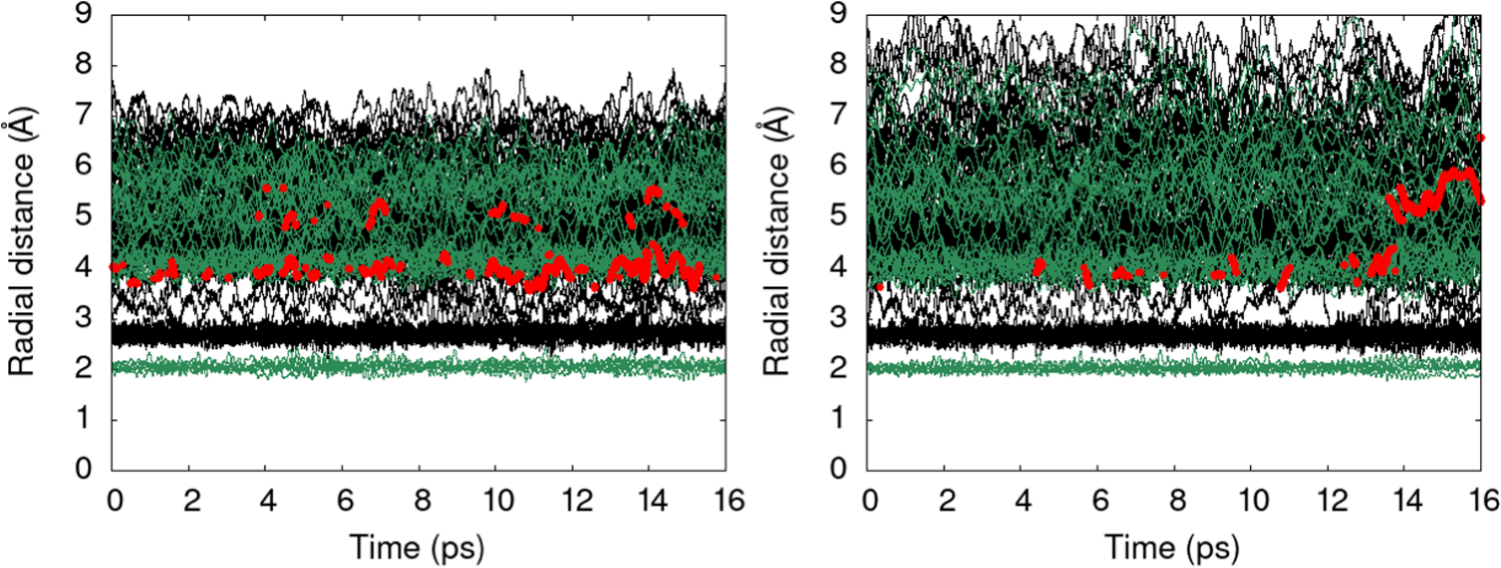
Distance distribution functions for hydrogen atoms (black) and oxygen atoms (green) for Fe(H_2_O)_6_^3+^ (PBE-D3, left and r^2^SCAN, right) as a function of time in AIMD. The distances are calculated with respect to the central metal ion. The distances of oxygen atoms coordinating 3 hydrogen atoms within a 1.2 Å radius region are highlighted in red.

One manifestation of this issue is illustrated in Figure 4. Here, a distance distribution analysis of the hydrogen and oxygen atoms around the metal ion is presented for the case of the Fe(H_2_O)_6_^3+^ AIMD trajectories. In addition to the distances of protons (black) and oxygen atoms (green) to the central ion, we highlight the distance of protons that leave the central ion (red). For the PBE-D3 trajectory, proton detachment from the first solvation shell occurs, and on several occasions an excess proton is observed in the second solvation shell. In comparison, this event occurs much less frequently in the r^2^SCAN trajectory. Hence, there is a pronounced difference in the configurational sampling for the two functionals. An analogous event can be seen when simulating complexes with high p*K*_a_ values, especially in the Ni^2+^ case. The additional graphs are provided in the Supporting Information (see Figure S1). As the deprotonated form of the hexaaqua ion is simulated, proton abstraction from a water molecule takes place, generating hydroxide, and its diffusion influences the configurational sampling. This explains why larger deviations are observed at the extreme ends of the p*K*_a_ scale.

**Figure 5:**
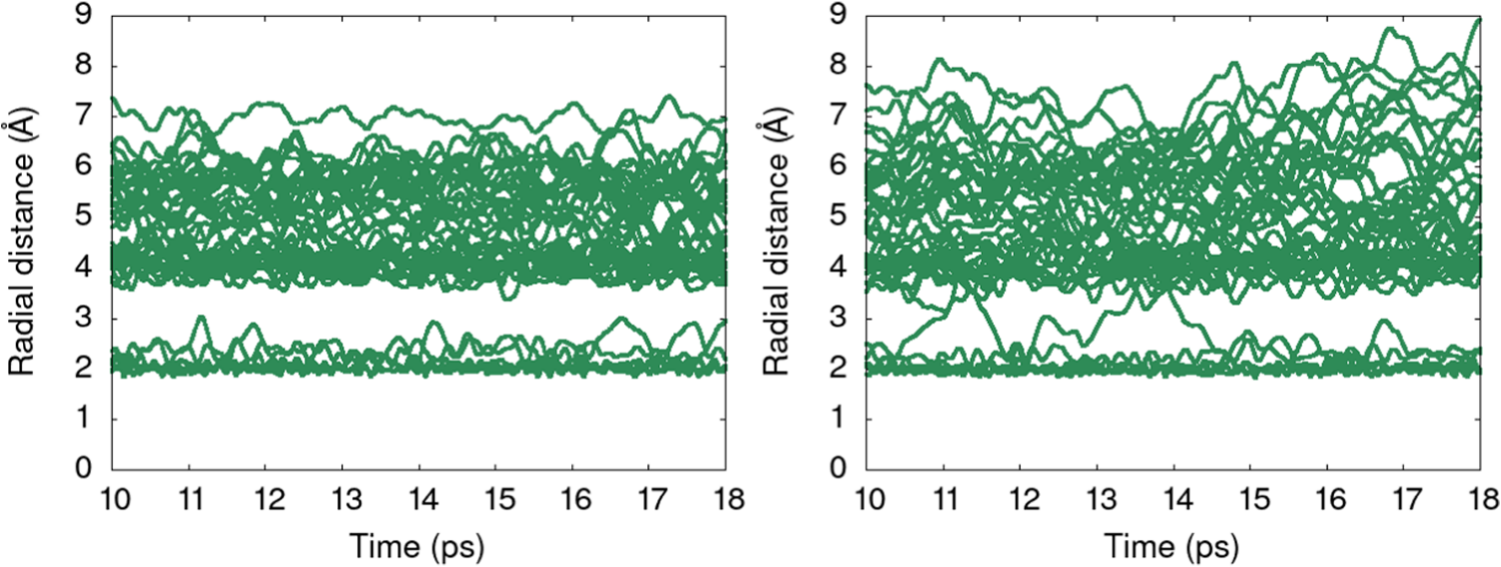
Distance distribution functions for oxygen atoms (green) for Cu(H_2_O)_6_^2+^ (PBE-D3, left and r^2^SCAN, right) as a function of time in AIMD. The distances are calculated with respect to the central metal ion.

Another case where configurational sampling imbalance between the two functionals can be seen is Cu^2+^ (Figure 5). Hexaaquacopper complex is known to undergo significant distortion.^58,59^ For this case, exchange of aquo ligands between the complex and the second solvation shell affects the hydrogen bond rearrangement between the first and the second solvation shells. This process is more hindered in PBE-D3 than for r^2^SCAN due to the water motion description. The distribution function illustrated in Figure 5 shows that there is more water ligand exchange between the first and second solvation shell, and subsequent increased fluctuations of the oxygen atom distances in the second and third solvation shell. As a consequence, r^2^SCAN and PBE-D3 sample different configurations which affects the respective average deprotonation energy.

Overall, we find that all structural features of water influence especially the p*K*_a_ values for protocol (iii). r^2^SCAN based AIMD simulations yield trajectories with more uniformity in the exploration of the accessible structures, which results in an overall more coherent p*K*_a_ scale, regardless of the chemical nature of the metal ion. Hence, while the explicit dynamic treatment of the solvent allows for a much more detailed description of solvent effects, the deficiencies of the underlying methods to describe water dynamics will occur as an additional source of error in the results.

## 4 p*K*_a_ and Redox Properties of the Ir_3_O_14_ Electrocatalyst

Transition metal oxides (TMO) are promising materials for OER electrocatalysis, and also a widely studied class of materials in computational simulations. Yet, many challenges still lie ahead for an accurate description of catalyst/electrolyte interface structure under the effect of an electrolyte at a given pH and electrode potential. The solvent can play multiple roles in the TMO catalyzed OER reaction and all relevant events along the reaction path need to be taken into consideration. Hence, computational models can, for example, be chosen as open systems in contact with proton and electron reservoirs in an efficient fashion and with reasonable accuracy. A previous study by our group focused on the description of the OER in nanoparticle models for iridium oxide, more specifically, Ir_3_O_14_,^23^ at the PBE-D3(CPCM)/def2-SVP level of theory. This model is small enough to allow for efficient computations, yet large enough to describe multiple effects on protonation and charge states. The resting state of the material could be clearly identified in the low pH and potential range. Furthermore, the structural changes in different potential and pH regions were studied: a first oxidation step occurs at *U* = 1.2 V, around pH 5 one deprotonation is observed and a second deprotonation occurs around pH 13.

**Figure 6:**
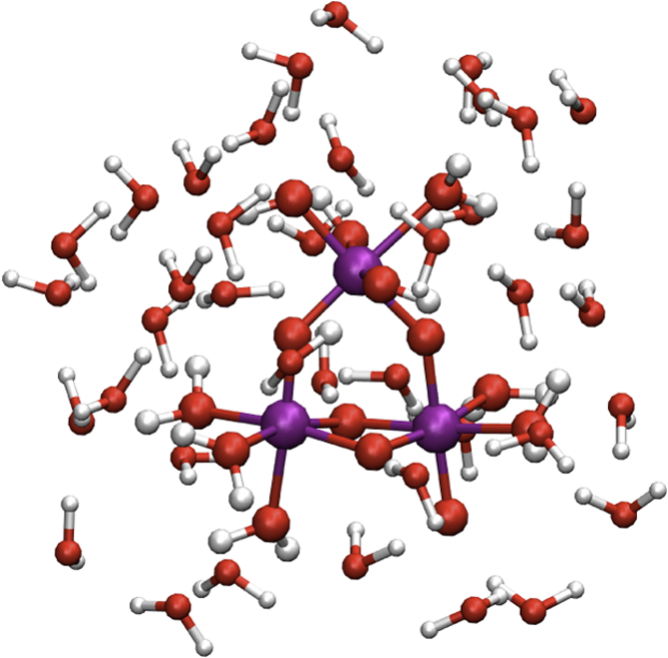
Illustration of the solvated Ir_3_O_14_ nanoparticle model.

**Figure 7:**
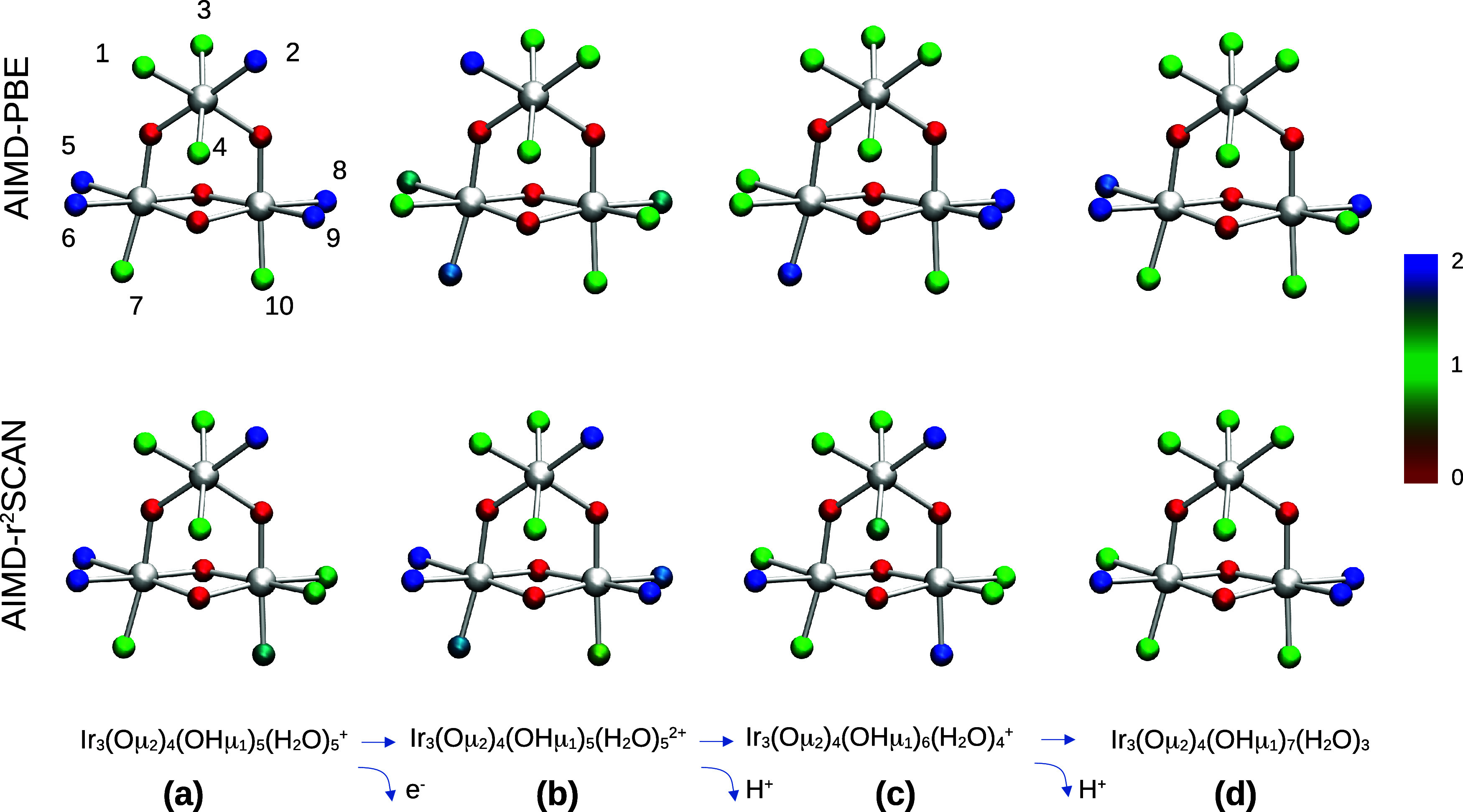
Average number of protons bound to each oxygen atom over the AIMD 10 ps trajectories for a Ir_3_O_14_ nanoparticle, PBE-D3 (upper panel) and r^2^SCAN (lower panel). Oxygen atoms are color coded for the average number of protons bound to the corresponding atom ranging from 0 (red) to 2 (blue). Ir atoms are depicted in white. Labels for the terminal oxygen atoms are displayed on the top left, with correspondence to the indexes in Tables 2 and 3.

In the following, we will use Ir_3_O_14_ to test the AIMD-based methodology introduced as protocol (iii) above. This allows to assess the dynamic structure of the interface, and whether the thermodynamic averages are comparable to the previously successfully applied static approach with implicit solvent. The four species considered in this study, labeled (a–d) in Figure 7 represent the intermediate structures of the oxidation/deprotonation processes referred to earlier. The structures are now solvated with an explicit layer of 39 water molecules and protocol (iii) is applied. An illustration of the modeled system with the water structure is shown in Figure 6. Deprotonation and oxidation energies are converted to p*K*_a_ and potential using the reference scales obtained for the hexaaqua ion systems, and the results are compared to the static implicit solvation method (Table 4). The average and standard deviation of the number of protons within 1.3 Å of each oxygen atom are shown in Figure 7 and Tables 2 and 3.

**Table 2:** Average Number of Protons within 1.3 Å of Each μ_1_ Oxygen Atom and Standard Deviation from AIMD with PBE-D3^a^

index	(a)	(b)	(c)	(d)
1	1.00 ± 0.02	1.99 ± 0.07	1.07 ± 0.25	1.00 ± 0.04
2	1.99 ± 0.06	1.00 ± 0.00	1.00 ± 0.08	1.00 ± 0.00
3	1.00 ± 0.00	1.00 ± 0.00	1.14 ± 0.35	1.01 ± 0.12
4	1.00 ± 0.00	1.00 ± 0.00	1.00 ± 0.00	1.00 ± 0.07
5	1.91 ± 0.29	1.48 ± 0.50	1.00 ± 0.02	1.68 ± 0.47
6	1.86 ± 0.34	1.00 ± 0.03	1.00 ± 0.00	1.98 ± 0.14
7	1.05 ± 0.26	1.61 ± 0.49	1.76 ± 0.42	1.01 ± 0.12
8	1.99 ± 0.02	1.49 ± 0.50	1.99 ± 0.05	1.86 ± 0.34
9	1.92 ± 0.26	1.00 ± 0.03	1.85 ± 0.36	1.00 ± 0.00
10	1.00 ± 0.06	1.00 ± 0.00	1.00 ± 0.00	1.07 ± 0.26
total	14.75 ± 0.58	12.58 ± 0.86	12.82 ± 0.71	12.63 ± 0.68

a The indexes are referenced in Figure 7.

**Table 3:** Average Number of Protons within 1.3 Å of Each μ_1_ Oxygen Atom and Standard Deviation from AIMD with r^2^SCAN Functional^a^

index	(a)	(b)	(c)	(d)
1	1.00 ± 0.00	1.00 ± 0.04	1.00 ± 0.02	1.00 ± 0.00
2	2.00 ± 0.00	1.02 ± 0.12	1.00 ± 0.00	1.00 ± 0.00
4	1.00 ± 0.00	1.00 ± 0.00	1.42 ± 0.49	1.00 ± 0.02
5	1.96 ± 0.20	1.98 ± 0.15	1.18 ± 0.39	1.00 ± 0.00
6	2.00 ± 0.00	2.00 ± 0.00	1.99 ± 0.07	1.86 ± 0.34
7	1.00 ± 0.00	1.56 ± 0.50	1.00 ± 0.00	0.99 ± 0.08
8	1.31 ± 0.46	1.62 ± 0.48	1.00 ± 0.05	1.99 ± 0.09
9	1.28 ± 0.45	1.82 ± 0.38	1.00 ± 0.00	1.95 ± 0.02
10	1.48 ± 0.50	0.64 ± 0.89	1.77 ± 0.41	1.00 ± 0.00
total	14.04 ± 0.84	14.52 ± 1.24	13.36 ± 0.76	12.80 ± 0.41

a The indexes are referenced in Figure 7.

From the proton distribution functions and standard deviations (Figure 7 and Tables 2 and 3) it is apparent that there are regions of significant proton fluctuation, and also oxygen sites with a well-defined proton occupation. The first oxidation process (see Figure 7(a,b)) leads to an increased positive charge of the system, and this has already a noteworthy effect on the acidity of some of the sites. Figure 7 and Tables 2 and 3 show that going from species (a) to (b), the average number of protons bound to the catalyst changes, but in a different way for each functional. The proton distribution on the catalyst in species (b) corresponds to H_2_O and OH groups for PBE-D3, while for r^2^SCAN oxo groups are observed. As the nature of the resulting species (b) is different, the corresponding redox potentials differ: PBE-D3 yields 1.17 V vs 1.87 V for r^2^SCAN (Table 4). This is in good agreement with what has been found for the static implicit model from our previous study: the local decrease of electron density by oxidation is often compensated by deprotonation events. However, while in the implicit model only discrete protonation states can be described, the explicit dynamic model yields average proton distributions, as sampled by AIMD.

**Table 4:** Average Oxidation and Deprotonation Energies for Ir_3_O_14_, for the Species in Figure 7 with Predicted p*K*_a_ and Redox Potentials Using the p*K*_a_-Redox Scales from Protocol (iii)^a^

	static (previous work)	PBE-D3 (iii)	r^2^SCAN (iii)
Δ*G*_a→b_	5.49 eV	4.62 eV	5.04 eV
Δ*G*_b→c_	11.50 eV	12.87 eV	12.60 eV
Δ*G*_c→d_	11.99 eV	11.68 eV	11.52 eV
*U*_a→b_	1.21 V	1.17 V	1.87 V
p*K*_ab→c_	5.09	8.47	6.66
p*K*_ac→d_	13.36	–0.20	–4.74

a The values are compared to the results of the previous study, performed at the PBE-D3(CPCM)/def2-SVP level.

As for the p*K*_a_ results using protocol (iii), different patterns for the hydrogen bonding network rearrangement emerge upon deprotonation (Figure 7, Tables 2 and 3). In the first deprotonation event (from species (b) to (c)), the proton distribution analysis from AIMD-r^2^SCAN shows that many sites are deprotonated and reprotonated, but the average net change of protons at the surface is still ∼−1. AIMD-PBE-D3 also displays multiple rearrangements in different sites, but this time the net change of protons at the surface is +0.2. This means that the net deprotonation event occurs at a solvent molecule rather than at the catalyst. The corresponding p*K*_a_ values of 6.66 for AIMD-r^2^SCAN and 8.47 for AIMD-PBE-D3 reflect that observation compared to 5.09 from the previously reported static implicit protocol study (see Table 4).

When considering the second deprotonation event (from species (c) to (d)), the AIMD results are remarkably different from the static implicit solvation protocol. The distribution functions show that proton rearrangement occurs in multiple sites, but the average net change of protons in the catalyst surface is less than 1 for both functionals. When comparing the corresponding p*K*_a_ calculation, instead of 13.36 (previous study), protocol (iii) predicts much lower values (−0.20 for PBE-D3 and −4.74 for r^2^SCAN).

These results illustrate, that when applying a dynamic explicit solvent scheme, the ”single process” description of deprotonation and oxidation is replaced by an average over several other possible events due to proton redistribution in the dynamics. Moreover, many configurations can now contribute to a given deprotonation or oxidation process, and AIMD is efficient in sampling these events.

## 5 Conclusions

A key aspect for computational studies on electrocatalytic processes is the inclusion of the electrochemical potential and the effect of the electrolyte with its pH and structure around the active site. In previous studies we have shown that this can be achieved using a combination of a cluster model for the active site with an implicit solvent model and explicitly adding and removing electrons and protons. In this study, pathways to extend this approach for the explicit inclusion of solvation were explored, reaching all the way to large-scale AIMD simulations with explicit solvent and connecting average ionization potentials and proton affinities with pH and potential.

**Figure 8:**
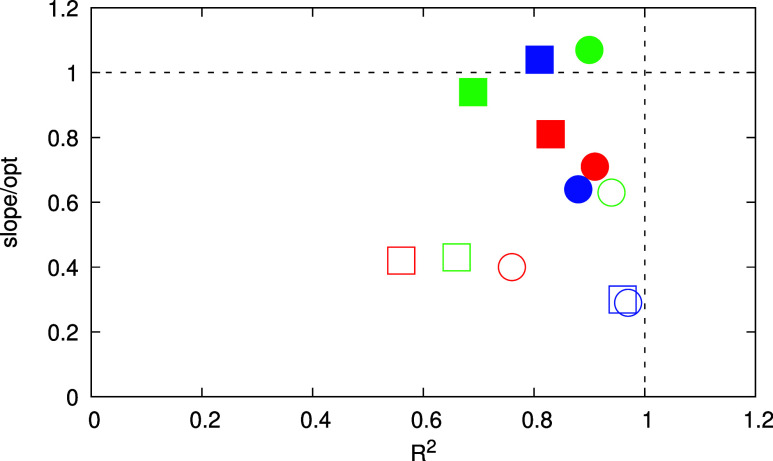
*R*^2^ and slope parameters from the different regression analyses for pH and redox scales normalized by the experimental scale parameters (square symbols for PBE-D3 and round for r^2^SCAN, filled symbols for redox scale and hollow for p*K*_a_). Results from protocols (i–iii) are colored in blue, red, and green, respectively. Dashed lines at the ideal values for each parameter are depicted as a guide to the eye.

For a series of molecular transition metal aquo complexes the results show that, given the right functional, the established and simple implicit solvent models yield excellent results for redox potentials and acceptable results for p*K*_a_ values. This is illustrated in Figure 8, where the *R*^2^ and the normalized slope from p*K*_a_ and redox scales are compared for all protocols and functionals.

For the redox potentials, a static explicit description of the solvent is counter productive while a dynamic explicit model yields results of satisfactory quality. For the p*K*_a_ values, however, a systematic improvement of the results can be observed from implicit, to explicit to explicit dynamic solvation in the case of r^2^SCAN. A detailed analysis shows that if explicit dynamic solvation is considered, the results not only depend on the description of the electronic structure of the central ion, but also on the description of the water dynamics of the given functional.

When applying the explicit dynamic protocol to a simple IrO_*x*_ OER catalyst model, the representation of a single protonation structure is extended to a distribution of protons with a given probability at different sites. Using appropriate functionals and models, averages over AIMD trajectories can be applied to capture the effects of microsolvation of a given active site. While this type of simulation is much more demanding computationally, it yields more consistent results and a more detailed and complete description of electrocatalytic processes. Furthermore, it not only allows for the inclusion of pH and potential effects, but also contains the physics for training machine learning potential energy functions, for example, offering an option to overcome current time and size limitations in explicit solvation simulation approaches.

In future applications this approach can play an important role in simulations for reactions in systems like electrolyzers and fuel cells, where the complex structure and dynamics of the solvent and reactant water are a key component.

## Supporting Information Available

xyz coordinates for the geometry optimized models considered, for implicit and explicit solvation; an additional description of the application of the p*K*_a_-U scale procedure for the model catalyst Ir_3_O_14_; a sample input file for ORCA AIMD simulations and supplementary distribution analysis of water atoms in AIMD transition metal complexes simulations, for tracking relevant changes in solvent structure upon oxidation/deprotonation (PDF)
