# Supplementary material for: Toward Explicit Solvation for Simulations of Electrocatalytic Reactions: AIMD for pKa and Redox Potentials of Transition Metal Compounds and Catalyst Models
Source: J Phys Chem A. 2025 Feb 3;129(6):1757–68. doi: 10.1021/acs.jpca.4c06898 (PMC11831669; doi:10.1021/acs.jpca.4c06898)
Supplement: Supplementary file 1 — jp4c06898_si_001.pdf [file jp4c06898_si_001.pdf]

Supporting Information: Towards explicit solvation for simulations of electrocatalytic reactions: AIMD for pKa and redox potentials of transition metal compounds and catalyst models

Gustavo T. Feliciano and Alexander A. Auer*

Department of Molecular Theory and Spectroscopy, Max-Planck-Institut für Kohlenforschung, 45470 Mülheim an der Ruhr, Germany

E-mail: alexander.auer@kofo.mpg.de

Contents

1 Distribution Functions for $M(H_2O)_5OH^+$ (M=Fe,Co,Ni) **S3**

2 Input sample for AIMD simulations **S4**

3 pKa-U grid procedure for IrOx simulation **S4**

4 Atomic coordinates from employed models **S6**

4.1 Molecular structure of the hexaaqua metal complexes, for protocol (i) calculations using PBE S6

4.2 Molecular structure of the hexaaqua metal complexes, for protocol (i) calculations using R2SCAN S20

4.3 Molecular structure of the hexaaqua metal complexes, for protocol (ii) calculations using PBE S35

4.4 Molecular structure of the hexaaqua metal complexes, for protocol (ii) calculations using R2SCAN S125

4.5 Molecular structure of initial Ir_3O_{14} configurations for protocol (iii) calculations using PBE S211

4.6 Molecular structure of initial Ir_3O_{14} configurations for protocol (iii) calculations using R2SCAN S233

1 Distribution Functions for $M(H_2O)_5OH^+$ (M=Fe,Co,Ni)

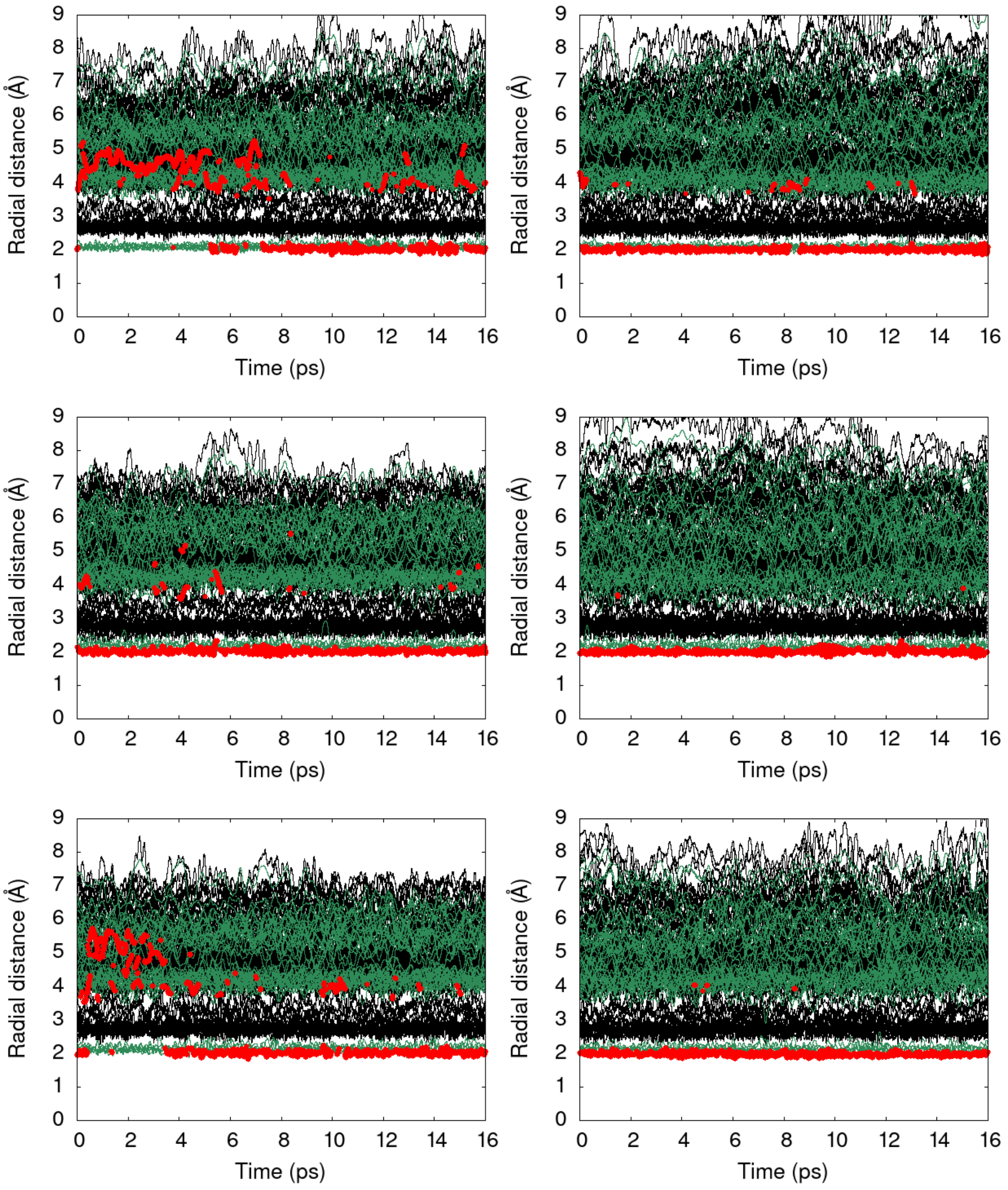

Figure S1: Distance distribution functions for hydrogen atoms (black) and oxygen atoms (green) in $Ni(H_2O)_5OH^+$ (upper panel), $Fe(H_2O)_5OH^+$ (middle panel) and $Co(H_2O)_5OH^+$ (lower panel), for the PBE-D3 (left) and r2SCAN (right) functionals, as a function of time in AIMD. The distances are calculated with respect to the central metal ion. The distances of oxygen atoms forming a hydroxide group (only one hydrogen atom within 1.2 Å radius), are highlighted in red.

2 Input sample for AIMD simulations

```
! MD PBE D3 def2-SVP CPCM(Water)
%md
initvel 300_K
timestep 0.5_fs
thermostat NHC 300_K timecon 20.0_fs
dump position stride 1 filename "trajectory.xyz"
dump velocity stride 1 filename "velocity.xyz"
run 20000 CenterCOM
end
%scf DirectResetFreq 200 end
%pal nprocs 8 end
%scf MaxIter 500 end
```

3 pKa-U grid procedure for IrOx simulation

In this section, we illustrate how to obtain pKa and U_0 values for deprotonation and oxidation processes using the pKa-U scale obtained from the hexa-aquo transition metal ion models. We first exemplify how the evaluation of redox potentials is performed. According to the fitting procedure in protocol (iii), we find in Figure 2 in the main manuscript, the equations Y = 25.7 X - 3.2 for PBE-D3 and Y = 29.0 X - 3.5 for r2SCAN, where Y is the U_0 value (in V), and X is the oxidation energy (in E_h) from AIMD trajectory average. From Table 4, in the manuscript, we can see that the oxidation energy is 4.62 eV (0.170 E_h) for PBE-D3 and 5.04 eV (0.185 E_h) for r2SCAN. Substituting these values in the respective equations yields 25.7 x 0.170 - 3.2 = 1.17 V for PBE-D3 and 25.7 x 0.185 - 3.2 = 1.87 V for r2SCAN.

The same is performed, regarding the pKa calculations, from AIMD deprotonation en-

ergies and the corresponding pKa scale. For example, also in the Figure 3, we obtain Y = 199.1 X -85.7 for PBE-D3 and Y = 288.9 X -127.1 for r2SCAN. Y is the pKa value, and X is the oxidation energy (in E_h) from AIMD trajectory average. From Table 4, we have, as deprotonation energies for the first deprotonation process, 12.87 eV (0.473 E_h) for PBE-D3 and 12.60 eV (0.463 E_h) for r2SCAN. Substituting in the corresponding equations, we have 199.1 x 0.4731 - 85.7 = 8.50 for PBE-D3 and 288.9 x 0.463 - 127.1 = 6.66 for r2SCAN.

The resulting pKa-U grid for IrOx is illustrated in the following scheme:

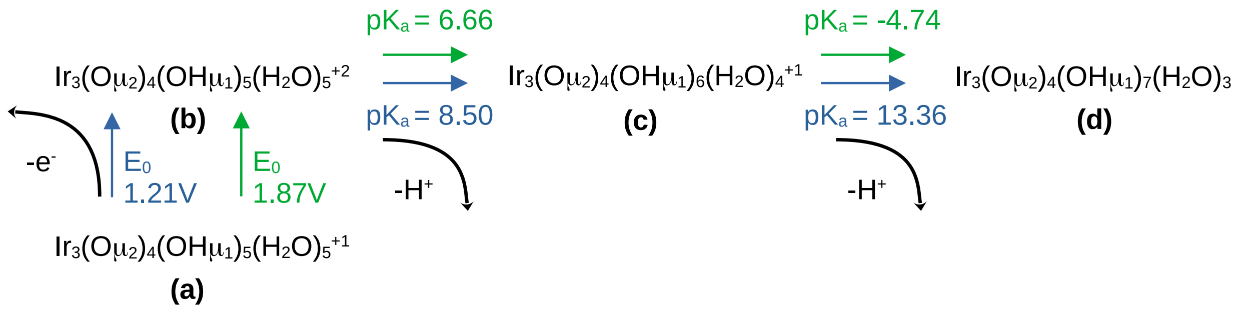

Figure S2: pH-U grid for IrOx, obtained from the AIMD trajectory averaged deprotonation and oxidation energies using r2SCAN, in Table 4. The value in blue is what is obtained from protocol (i), for comparison.

As an additional reference, we also supply the structure of the IrOx species corresponding to the deprotonation and oxidation processes studied, from the static protocol, obtained in the previous study (J. Phys. Chem. C 2021, 125, 8, 4379–4390)

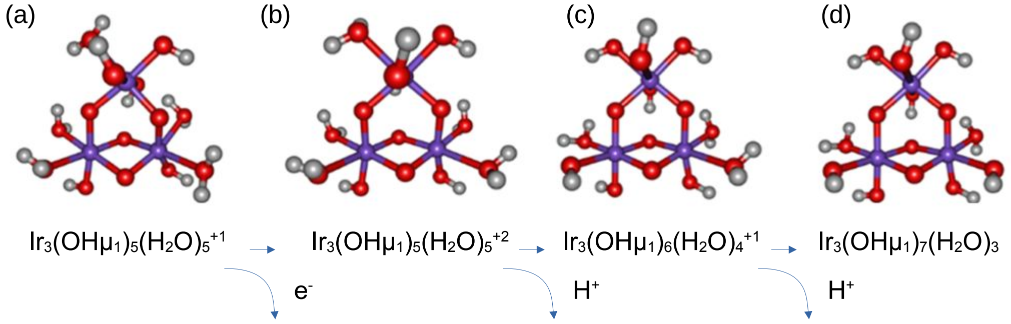

Figure S3: Illustration of the IrOx species studied with AIMD. The initial structures come from the previous study (J. Phys. Chem. C 2021, 125, 8, 4379–4390) with implicit pH/U protocol for iridium oxides under the static protocol.

4 Atomic coordinates from employed models

4.1 Molecular structure of the hexaaqua metal complexes, for protocol (i) calculations using PBE

XYZ Coordinates from $Fe(H_2O)_6^{2+}$

Fe 14.294192 15.397699 14.835209

O 15.745059 14.754296 16.281538

H 16.387540 15.438771 16.561992

H 16.297088 13.983695 16.033081

O 12.946558 15.190971 16.513762

H 12.199835 14.570753 16.382209

H 13.459210 14.800518 17.252823

O 14.653343 17.400048 15.418948

H 14.230188 17.699629 16.249273

H 14.526628 18.139442 14.790158

O 12.635934 15.987564 13.669937

H 12.014750 15.271331 13.422767

H 12.068846 16.695239 14.040917

O 13.930320 13.398634 14.282058

H 14.114815 12.997884 13.409062

H 13.958511 12.660293 14.923230

O 15.822094 15.577865 13.377322

H 16.579446 16.145847 13.631437

H 16.238185 14.738970 13.088014

XYZ Coordinates from $Co(H_2O)_6^{2+}$

Co 14.346870 15.314360 14.880324

O 15.931943 14.777857 16.132754

H 16.800311 15.176654 15.918405

H 16.119006 13.829390 16.288943

O 13.055718 15.062775 16.510476

H 12.133836 15.366241 16.380367

H 12.973837 14.175178 16.916882

O 14.649461 17.348296 15.427712

H 14.156025 17.584172 16.240874

H 14.389717 18.042447 14.786907

O 12.688221 15.891603 13.736655

H 11.894034 15.323036 13.811802

H 12.349368 16.803817 13.849824

O 14.084365 13.331881 14.276602

H 14.852008 12.726322 14.332969

H 13.346448 12.835440 14.687449

O 15.720541 15.659946 13.342228

H 16.125546 16.552094 13.323029

H 16.485287 15.047942 13.319534

XYZ Coordinates from $Cu(H_2O)_6^{2+}$

Cu 14.291527 15.864868 14.874717

O 16.050346 15.752291 15.883553

H 16.707510 16.392720 15.533509

H 16.456447 14.875414 15.704020

O 13.399777 15.023257 16.514693

H 13.256363 15.901468 16.956613

H 12.492171 14.739507 16.266912

O 13.707093 17.649808 16.210524

H 14.407285 18.231758 16.569842

H 13.013397 18.263071 15.894545

O 12.480616 15.784608 13.926290

H 12.440212 16.481280 13.234807

H 12.684609 14.951397 13.427925

O 14.345997 13.903662 13.656499

H 15.010139 13.840765 12.940252

H 14.443734 13.065945 14.152776

O 15.072057 16.877658 13.298271

H 15.262851 17.804999 13.561877

H 15.944468 16.515530 13.027923

XYZ Coordinates from $Ni(H_2O)_6^{2+}$

Ni 14.321294 15.308931 14.956382

O 15.739421 14.597019 16.297989

H 16.406770 15.287134 16.497269

H 16.261596 13.840146 15.958879

O 12.971131 15.251598 16.532394

H 12.094090 14.881762 16.298604

H 13.308484 14.658060 17.236461

O 14.822935 17.217697 15.609111

H 14.112683 17.557576 16.193771

H 14.889840 17.883989 14.893372

O 12.903615 16.020581 13.613997

H 12.245839 15.324310 13.404468

H 12.370645 16.767389 13.958565

O 13.819360 13.400075 14.302894

H 14.530593 13.058914 13.720196

H 13.748310 12.733434 15.017900

O 15.671297 15.366023 13.380017

H 15.335534 15.961382 12.676746

H 16.549104 15.733432 13.614721

XYZ Coordinates from $Fe(H_2O)_6^{3+}$

Fe 14.355803 15.295983 14.977823

O 15.886389 14.738393 16.167289

H 16.485913 15.356896 16.640053

H 16.263700 13.833323 16.229307

O 13.078141 15.012031 16.518164

H 12.099141 15.066564 16.457693

H 13.313911 14.601225 17.378977

O 14.593116 17.222622 15.555377

H 14.018918 17.657710 16.223868

H 15.004246 17.923727 15.002906

O 12.820706 15.851254 13.781206

H 12.221870 15.228356 13.313461

H 12.432240 16.751976 13.725151

O 14.094493 13.376769 14.390493

H 14.379619 13.025851 13.517800

H 13.400640 12.779126 14.747592

O 15.636555 15.586406 13.442777

H 15.401714 16.005523 12.585718

H 16.615428 15.535718 13.508081

XYZ Coordinates from $Co(H_2O)_6^{3+}$

Co 14.297365 15.374755 14.886153

O 15.676245 14.814549 16.262063

H 16.275709 15.490660 16.651696

H 16.231417 14.022309 16.083810

O 12.983171 15.133692 16.454576

H 12.143630 14.665299 16.243353

H 13.375295 14.643324 17.213018

O 14.547263 17.277042 15.368429

H 14.637089 17.640020 16.278653

H 14.591009 18.028067 14.733232

O 12.726162 15.889792 13.762404

H 12.113339 15.223528 13.374302

H 12.185929 16.680590 13.994369

O 14.087666 13.474821 14.354711

H 13.985027 13.113930 13.444877

H 14.043419 12.723115 14.988526

O 15.687869 15.598233 13.450834

H 16.414080 16.251354 13.579011

H 16.100858 14.804374 13.039719

XYZ Coordinates from $Cu(H_2O)_6^{3+}$

Cu 14.322744 15.308401 14.956751

O 15.705733 14.650280 16.304670

H 16.395520 15.349512 16.415603

H 16.197120 13.855139 15.984117

O 13.006359 15.276543 16.499931

H 12.123683 14.909305 16.248605

H 13.351095 14.658829 17.190620

O 14.838489 17.160388 15.623675

H 14.112383 17.474621 16.216747

H 14.883090 17.832904 14.901086

O 12.935898 15.964692 13.611099

H 12.251709 15.260309 13.498088

H 12.438818 16.755377 13.933802

O 13.802348 13.458627 14.286204

H 14.527541 13.146346 13.690873

H 13.759911 12.784343 15.007346

O 15.635318 15.340434 13.409456

H 15.293609 15.964515 12.723111

H 16.521173 15.698889 13.661952

XYZ Coordinates from $Ir(H_2O)_6^{3+}$

Ir 14.331823 15.311312 14.947723

O 15.749544 14.629159 16.293158

H 16.432874 15.302243 16.524634

H 16.248113 13.829713 16.000265

O 13.021950 15.223924 16.546506

H 12.116176 14.896692 16.334086

H 13.349858 14.649564 17.279286

O 14.797945 17.208530 15.636058

H 14.120231 17.556638 16.263829

H 14.882515 17.896553 14.934001

O 12.916780 15.995516 13.601683

H 12.285245 15.296011 13.308757

H 12.361015 16.743062 13.926379

O 13.869126 13.418229 14.246189

H 14.638314 12.938153 13.856649

H 13.491744 12.808696 14.923479

O 15.647334 15.406807 13.353995

H 15.305213 15.965298 12.615367

H 16.536740 15.773351 13.571691

XYZ Coordinates from $Ni(H_2O)_6^{3+}$

Ni 14.321375 15.307533 14.957168

O 15.691585 14.588765 16.244210

H 16.295206 15.309156 16.544672

H 16.288186 13.898847 15.869309

O 13.026184 15.279019 16.484019

H 12.123146 14.955488 16.253077

H 13.339093 14.685796 17.207851

O 14.796790 17.034135 15.551279

H 14.097706 17.366845 16.171768

H 14.857212 17.709724 14.828922

O 12.952638 16.027254 13.667411

H 12.347673 15.307474 13.368106

H 12.357235 16.718346 14.042112

O 13.845912 13.581247 14.362318

H 14.545037 13.250340 13.740894

H 13.785764 12.904298 15.083396

O 15.615707 15.335792 13.427948

H 15.297605 15.926612 12.704394

H 16.518486 15.662781 13.654886

XYZ Coordinates from $Fe(H_2O)_5OH^+$

Fe 14.459297 15.044511 14.549195

O 14.606196 14.320889 16.302843

H 14.033343 13.541978 16.447318

O 13.801365 16.446920 17.548063

H 12.827668 16.395874 17.473058

H 14.099651 15.525800 17.202771

O 14.524097 17.149370 15.157631

H 14.233051 17.049974 16.139645

H 13.828831 17.706450 14.754169

O 12.469080 15.407372 13.938588

H 11.906679 14.675678 13.612506

H 11.912376 15.878770 14.591135

O 14.464885 13.344634 13.245454

H 15.251246 12.764297 13.293920

H 13.709269 12.726468 13.315578

O 16.083483 15.763921 13.407117

H 16.411278 16.573148 13.852082

H 16.873628 15.212195 13.239539

XYZ Coordinates from $Co(H_2O)_5OH^+$

Co 14.220103 15.307320 14.940139

O 15.203206 15.162930 16.728430

H 15.989016 14.586678 16.754803

O 12.753172 14.942778 16.501082

H 12.355442 14.051490 16.444195

H 13.650056 14.812281 16.978839

O 14.530120 17.309939 15.709917

H 15.178844 17.824801 15.190437

H 15.083506 16.722967 16.341962

O 12.891880 15.807045 13.372435

H 11.974992 15.476530 13.461378

H 12.793788 16.762853 13.186066

O 14.074178 13.310166 14.294800

H 13.699145 12.651498 14.913664

H 13.578265 13.177680 13.460908

O 15.752158 15.576653 13.516251

H 15.387498 15.730272 12.620084

H 16.380055 16.314366 13.655221

XYZ Coordinates from $Cu(H_2O)_5OH^+$

Cu 14.104788 16.123050 15.547203

O 15.722484 15.576165 16.379429

H 16.361285 16.317428 16.337387

O 13.277065 14.456473 16.363843

H 13.201650 13.878956 15.532127

H 14.044544 14.094436 16.859889

O 14.774514 17.906649 14.881319

H 14.800107 18.562382 15.611359

H 14.092820 18.254326 14.265977

O 12.610449 16.183814 14.158957

H 12.704113 15.239633 13.809054

H 11.731415 16.199920 14.593127

O 13.355718 13.670562 13.895707

H 14.376631 13.782473 13.856197

H 13.116476 12.870105 13.390088

O 15.882765 14.242495 14.096023

H 16.103021 14.955652 13.463960

H 15.960718 14.705249 15.004408

XYZ Coordinates from $Ir(H_2O)_4(OH)_2^+$

Ir 14.378212 15.389034 14.813174

O 15.978613 14.531627 15.647584

H 15.856864 14.546317 16.620504

O 13.451172 15.433101 16.593115

H 12.483090 15.338848 16.476970

O 14.627104 17.325194 15.539797

H 14.047193 17.052897 16.339078

H 14.070689 17.958270 15.030023

O 12.677995 16.285993 13.809290

H 12.747742 16.234996 12.832067

H 11.830832 15.835558 14.017325

O 14.244211 13.352859 14.368684

H 15.066408 13.211765 14.962990

H 13.498876 12.926961 14.846609

O 15.598173 15.394352 13.027473

H 15.687602 16.284386 12.625354

H 16.509375 15.179460 13.331011

XYZ Coordinates from $Ni(H_2O)_5OH^+$

Ni 14.338079 15.329326 14.973865

O 15.430992 14.553569 16.458507

H 15.471615 13.591230 16.291075

O 13.126406 15.287823 16.716817

H 12.484475 14.551254 16.681308

H 14.077180 14.832530 16.938327

O 14.730760 17.304026 15.556790

H 13.994027 17.527427 16.164514

H 14.627601 17.936369 14.816217

O 13.000342 16.111848 13.533433

H 12.454987 15.367169 13.204210

H 12.349843 16.729982 13.925056

O 13.774303 13.459667 14.195876

H 14.545854 13.136382 13.684503

H 13.628673 12.769779 14.875775

O 15.714504 15.346718 13.397153

H 15.363128 15.942984 12.702906

H 16.580688 15.736737 13.634134

XYZ Coordinates from $Fe(H_2O)_5OH^{2+}$

Fe 14.526050 15.282604 15.103287

O 15.794694 14.542350 16.158523

H 15.950551 13.584583 16.297959

O 13.010085 15.183810 16.529804

H 12.068822 15.117669 16.263034

H 13.129101 14.575649 17.289790

O 14.738373 17.305133 15.595902

H 14.055842 17.659858 16.205886

H 14.792942 17.950699 14.858742

O 12.977523 15.928612 13.758452

H 12.407816 15.209962 13.411284

H 12.370521 16.653317 14.017849

O 13.883125 13.418135 14.260866

H 14.615270 12.926883 13.829043

H 13.514841 12.790237 14.919407

O 15.680433 15.555571 13.410281

H 15.332609 16.132332 12.697412

H 16.646824 15.710846 13.463089

XYZ Coordinates from $Co(H_2O)_5OH^{2+}$

Co 14.226291 15.348872 14.892655

O 15.419849 14.929417 16.217245

H 15.851133 14.072390 16.011541

O 12.927555 15.347012 16.305039

H 12.161680 14.739891 16.185527

H 13.485462 14.959458 17.030038

O 14.646727 17.176371 15.330209

H 15.397633 17.054101 15.965367

H 13.930611 17.576977 15.877329

O 12.947932 15.948669 13.496833

H 12.346396 15.225916 13.211414

H 12.349733 16.679969 13.764575

O 13.830833 13.528315 14.402451

H 14.509490 13.197383 13.768529

H 13.855371 12.893388 15.155249

O 15.607738 15.325828 13.554417

H 15.513965 16.073731 12.920099

H 16.487025 15.450562 13.982094

XYZ Coordinates from $Ir(H_2O)_5OH^{2+}$

Ir 14.231444 15.347378 14.915693

O 15.385639 14.933434 16.486292

H 15.912438 14.120926 16.343309

O 12.933352 15.266296 16.527329

H 12.215840 14.594267 16.472331

H 13.624040 14.899756 17.164081

O 14.778931 17.252082 15.506226

H 15.393776 17.072778 16.265779

H 14.045346 17.791708 15.881800

O 12.970645 15.943119 13.271805

H 12.357218 15.228641 12.989030

H 12.395968 16.717530 13.455084

O 13.795014 13.419175 14.256663

H 14.597457 12.952244 13.926166

H 13.423011 12.824091 14.947037

O 15.648928 15.400695 13.392411

H 15.299332 15.913193 12.625080

H 16.487043 15.850934 13.644495

XYZ Coordinates from $Cu(H_2O)_6^+$

Cu 15.008382 15.322764 15.581394

O 16.894061 14.702828 15.407159

H 17.505284 15.465356 15.366421

H 16.976540 14.264157 14.536047

O 13.259934 15.632838 16.425510

H 13.248698 16.630097 16.384543

H 12.679587 15.350913 15.643773

O 13.383540 18.336776 15.926068

H 13.648874 18.950845 16.641163

H 12.503735 18.659995 15.640650

O 12.073386 14.961654 14.192667

H 12.108484 15.828350 13.744301

H 12.912195 14.519460 13.884026

O 14.475705 13.971898 13.593013

H 14.889191 14.473607 12.862689

H 14.683433 13.032299 13.418809

O 14.920125 16.937194 13.980196

H 14.465609 17.611817 14.548719

H 15.829835 17.267160 13.858402

4.2 Molecular structure of the hexaaqua metal complexes, for protocol (i) calculations using R2SCAN

XYZ Coordinates from $Fe(H_2O)_6^{2+}$

Fe 14.304587 15.376022 14.867182

O 15.761872 14.779918 16.292853

H 16.402462 15.446963 16.590276

H 16.291316 13.984278 16.118285

O 12.939593 15.166367 16.508706

H 12.106195 14.701254 16.327842

H 13.337202 14.684355 17.252869

O 14.665764 17.377849 15.403949

H 14.415851 17.694032 16.286980

H 14.506871 18.127633 14.808306

O 12.651841 15.942796 13.701124

H 12.006226 15.268539 13.432728

H 12.121515 16.708744 13.976720

O 14.009447 13.376490 14.313630

H 14.063539 13.002001 13.420312

H 14.051540 12.622207 14.922426

O 15.767102 15.579680 13.365677

H 16.472703 16.232273 13.508506

H 16.226912 14.778050 13.065365

XYZ Coordinates from $Co(H_2O)_6^{2+}$

Co 14.321581 15.308458 14.955122

O 15.948335 14.741536 16.117251

H 16.815932 15.155231 15.978956

H 16.131808 13.800451 16.269943

O 13.020625 14.952193 16.534059

H 12.109249 15.283527 16.485707

H 12.951144 14.066522 16.926265

O 14.530264 17.288044 15.552481

H 13.750516 17.859265 15.642959

H 15.226905 17.856920 15.186529

O 12.694835 15.874150 13.792874

H 11.827054 15.461371 13.932686

H 12.511914 16.816285 13.646010

O 14.110518 13.328068 14.358920

H 14.888927 12.753200 14.280443

H 13.407473 12.764283 14.720345

O 15.624097 15.663899 13.376855

H 15.697538 16.548120 12.982164

H 16.533827 15.327928 13.424168

XYZ Coordinates from $Cu(H_2O)_6^{2+}$

Cu 14.241929 15.914797 14.855244

O 15.993473 15.833504 15.774305

H 16.702598 15.518987 15.184616

H 15.941754 15.167350 16.484759

O 13.472692 14.843066 16.499046

H 12.916817 15.449045 17.018066

H 12.888141 14.097746 16.281405

O 13.769392 17.675269 16.007823

H 14.544626 18.195378 16.275139

H 13.173482 18.321879 15.596028

O 12.468513 16.038494 13.986617

H 12.540406 16.645914 13.226974

H 12.248688 15.174755 13.590876

O 14.503449 14.030207 13.844858

H 15.034352 14.043132 13.031154

H 14.930137 13.354046 14.397131

O 14.998907 17.083387 13.282594

H 15.453810 17.880884 13.602796

H 15.643424 16.652156 12.696111

XYZ Coordinates from $Ni(H_2O)_6^{2+}$

Ni 14.321676 15.308731 14.956353

O 15.747464 14.618516 16.250790

H 16.415442 15.283411 16.488208

H 16.254637 13.841233 15.962755

O 12.988429 15.214470 16.503644

H 12.092820 14.903640 16.290200

H 13.289378 14.641630 17.229066

O 14.787871 17.200074 15.584380

H 14.113118 17.572972 16.176543

H 14.914726 17.871289 14.893185

O 12.895857 16.001216 13.662673

H 12.239959 15.329503 13.411016

H 12.374511 16.764478 13.962677

O 13.855449 13.418229 14.325808

H 14.532435 13.043045 13.737708

H 13.718170 12.745811 15.013777

O 15.653471 15.402719 13.407529

H 15.355698 15.982062 12.685954

H 16.551429 15.706423 13.621472

XYZ Coordinates from $Fe(H_2O)_6^{3+}$

Fe 14.330230 15.301061 14.963960

O 15.862924 14.746453 16.132137

H 16.446825 15.358704 16.617085

H 16.228393 13.845728 16.208911

O 13.065348 15.009738 16.493758

H 12.094968 15.091893 16.445508

H 13.300503 14.621429 17.356797

O 14.585522 17.203457 15.553760

H 14.080795 17.630792 16.270374

H 15.051990 17.895974 15.050084

O 12.800160 15.863696 13.794452

H 12.227691 15.258627 13.287274

H 12.440255 16.766432 13.714896

O 14.075574 13.400980 14.377945

H 14.502047 12.989231 13.603813

H 13.482032 12.746449 14.790409

O 15.596783 15.593531 13.434405

H 15.362507 15.998041 12.578553

H 16.567996 15.527237 13.489617

XYZ Coordinates from $Co(H_2O)_6^{3+}$

Co 14.297365 15.374755 14.886153

O 15.676245 14.814549 16.262063

H 16.275709 15.490660 16.651696

H 16.231417 14.022309 16.083810

O 12.983171 15.133692 16.454576

H 12.143630 14.665299 16.243353

H 13.375295 14.643324 17.213018

O 14.547263 17.277042 15.368429

H 14.637089 17.640020 16.278653

H 14.591009 18.028067 14.733232

O 12.726162 15.889792 13.762404

H 12.113339 15.223528 13.374302

H 12.185929 16.680590 13.994369

O 14.087666 13.474821 14.354711

H 13.985027 13.113930 13.444877

H 14.043419 12.723115 14.988526

O 15.687869 15.598233 13.450834

H 16.414080 16.251354 13.579011

H 16.100858 14.804374 13.039719

XYZ Coordinates from $Cu(H_2O)_6^{3+}$

Cu 14.322106 15.308376 14.957946

O 15.695226 14.649462 16.247499

H 16.370851 15.340707 16.410058

H 16.192169 13.861820 15.942062

O 13.011961 15.255934 16.456332

H 12.129475 14.898503 16.223094

H 13.344537 14.679770 17.176440

O 14.820564 17.136550 15.590291

H 14.129952 17.472230 16.199879

H 14.883914 17.805241 14.876333

O 12.951185 15.978050 13.672338

H 12.306974 15.271596 13.456622

H 12.418736 16.730982 14.003936

O 13.821604 13.482496 14.320871

H 14.515325 13.146510 13.714914

H 13.753034 12.813017 15.033639

O 15.626316 15.361464 13.454087

H 15.294888 15.948579 12.742282

H 16.513724 15.708165 13.685115

XYZ Coordinates from $Ir(H_2O)_6^{3+}$

Ir 14.330813 15.309852 14.949363

O 15.756198 14.642000 16.272702

H 16.419625 15.313063 16.531861

H 16.259561 13.849345 15.996531

O 13.030489 15.209354 16.536753

H 12.108680 14.954875 16.331021

H 13.325785 14.621346 17.261148

O 14.783820 17.199550 15.626473

H 14.128050 17.554126 16.261076

H 14.888188 17.887665 14.938458

O 12.907471 15.978495 13.624988

H 12.278313 15.291049 13.325920

H 12.366949 16.738170 13.922400

O 13.882161 13.424212 14.260439

H 14.626839 12.954867 13.832831

H 13.512546 12.804683 14.921005

O 15.637770 15.414849 13.367328

H 15.319748 15.970729 12.627071

H 16.539536 15.731221 13.576368

XYZ Coordinates from $Ni(H_2O)_6^{3+}$

Ni 14.321959 15.303224 14.957163

O 15.741145 14.635162 16.214464

H 16.353801 15.347535 16.490527

H 16.307267 13.920742 15.856803

O 13.036309 15.062912 16.370498

H 12.075508 14.943144 16.244487

H 13.263048 15.036218 17.320162

O 14.827010 17.148830 15.685388

H 14.153325 17.512696 16.294007

H 14.902449 17.819051 14.976463

O 12.904085 15.970615 13.694700

H 12.293152 15.254928 13.423099

H 12.336327 16.684116 14.051737

O 13.819402 13.458996 14.227303

H 14.494224 13.096402 13.619248

H 13.742436 12.788025 14.935288

O 15.604356 15.545249 13.540526

H 15.365793 15.624929 12.596632

H 16.560943 15.696680 13.665243

XYZ Coordinates from $Fe(H_2O)_5OH^+$

Fe 13.813789 14.975731 14.585910

O 13.723381 14.417229 16.406058

H 14.119611 13.548625 16.571203

O 15.254981 16.403856 16.905947

H 15.585070 16.594396 17.795031

H 14.670467 15.567080 16.949756

O 13.531460 17.061447 15.020387

H 14.110291 17.159684 15.819022

H 12.649227 17.347801 15.304641

O 12.227288 15.160036 13.251338

H 12.210788 14.665736 12.416174

H 11.836001 16.025156 13.049846

O 14.431053 13.350574 13.401833

H 15.351976 13.066943 13.514003

H 13.921846 12.540433 13.242588

O 15.911826 15.508944 14.504122

H 16.147315 16.244263 13.915620

H 15.999053 15.890316 15.417132

XYZ Coordinates from $Co(H_2O)_5OH^+$

Co 14.231329 15.299754 14.939264

O 15.229509 15.137338 16.680905

H 16.006897 14.564049 16.713513

O 12.765310 14.945868 16.490129

H 12.333242 14.078569 16.459608

H 13.635264 14.803851 16.977858

O 14.506234 17.300509 15.713324

H 15.119145 17.866005 15.218988

H 15.076480 16.751143 16.335340

O 12.882330 15.798350 13.417146

H 11.964005 15.496427 13.504660

H 12.818546 16.728383 13.146758

O 14.051056 13.321521 14.290379

H 13.693212 12.652318 14.895331

H 13.581364 13.180211 13.452303

O 15.752412 15.599117 13.542357

H 15.466506 15.679651 12.617830

H 16.382583 16.325185 13.674917

XYZ Coordinates from $Cu(H_2O)_5OH^+$

Cu 14.131162 16.094710 15.524377

O 15.714924 15.669505 16.426661

H 16.336614 16.412154 16.364024

O 13.332791 14.435314 16.324881

H 13.198963 13.847525 15.529220

H 14.059614 14.035447 16.834424

O 14.760197 17.857028 14.828221

H 14.930698 18.511464 15.527545

H 14.073976 18.256574 14.265984

O 12.610932 16.160200 14.208786

H 12.646304 15.240189 13.827151

H 11.743837 16.224184 14.644514

O 13.311304 13.638882 13.842990

H 14.306848 13.765603 13.820495

H 13.101169 12.846223 13.327307

O 15.843535 14.304468 14.159766

H 16.147053 14.978106 13.531618

H 15.970643 14.742191 15.058090

XYZ Coordinates from $Ir(H_2O)_4(OH)_2^+$

Ir 14.328678 15.417048 14.734696

O 15.923252 14.581760 15.586572

H 16.128141 14.999251 16.436939

O 12.955043 15.451440 16.176613

H 12.778114 14.554667 16.501817

O 14.708140 17.354080 15.363008

H 14.208471 17.479878 16.195112

H 14.355328 18.030308 14.753979

O 12.564889 16.247845 13.833119

H 12.199214 15.737103 13.090406

H 12.007012 16.023218 14.622102

O 14.067685 13.428460 14.232049

H 14.753066 13.008895 14.800910

H 13.217159 13.054902 14.529020

O 15.865040 15.436605 13.229772

H 16.125655 16.324949 12.931416

H 16.569264 15.151208 13.863515

XYZ Coordinates from $Ni(H_2O)_5OH^+$

Ni 14.311423 15.335005 14.957515

O 15.457497 14.601037 16.386904

H 15.564274 13.652406 16.221549

O 13.115091 15.235749 16.688627

H 12.487831 14.496494 16.694963

H 14.046525 14.821220 16.921382

O 14.688659 17.299860 15.511063

H 14.003414 17.591819 16.135317

H 14.642962 17.943811 14.785631

O 12.953280 16.095672 13.574424

H 12.397295 15.383805 13.216621

H 12.322808 16.733052 13.947401

O 13.759274 13.463035 14.224366

H 14.484626 13.129038 13.669994

H 13.638567 12.779041 14.903638

O 15.696798 15.366687 13.426435

H 15.516555 16.053191 12.763502

H 16.606577 15.533898 13.721135

XYZ Coordinates from $Fe(H_2O)_5OH^{2+}$

Fe 14.491455 15.272919 15.111892

O 15.759155 14.574454 16.176266

H 15.974739 13.636801 16.306941

O 12.993428 15.155384 16.522042

H 12.048208 15.136451 16.294770

H 13.110785 14.610529 17.318934

O 14.694898 17.283388 15.581032

H 14.043717 17.674325 16.189809

H 14.828428 17.934780 14.870396

O 12.948606 15.905967 13.796443

H 12.332277 15.240794 13.445833

H 12.423331 16.707151 13.960834

O 13.898992 13.416970 14.284732

H 14.545776 13.043963 13.659418

H 13.726849 12.706392 14.927536

O 15.664704 15.553646 13.451131

H 15.374726 16.081140 12.687378

H 16.635349 15.593194 13.485225

XYZ Coordinates from $Co(H_2O)_5OH^{2+}$

Co 14.250273 15.335102 14.912254

O 15.469210 14.911990 16.182377

H 15.892206 14.066881 15.951546

O 12.937680 15.315811 16.290809

H 12.115194 14.821879 16.109038

H 13.366014 14.886357 17.060059

O 14.627075 17.158935 15.330286

H 15.401931 17.115558 15.929359

H 13.918284 17.560696 15.870894

O 12.957959 15.916354 13.548216

H 12.309544 15.237101 13.288430

H 12.436584 16.716265 13.739380

O 13.862251 13.529036 14.432731

H 14.507228 13.179348 13.786973

H 13.868435 12.895726 15.176724

O 15.601088 15.343403 13.567932

H 15.490664 16.070432 12.924521

H 16.483803 15.467378 13.969081

XYZ Coordinates from $Ir(H_2O)_5OH^{2+}$

Ir 14.233907 15.339338 14.927693

O 15.423469 14.896263 16.439542

H 15.899650 14.067273 16.270080

O 12.909628 15.273301 16.502149

H 12.165806 14.646236 16.416791

H 13.504180 14.908047 17.202362

O 14.723699 17.258059 15.487721

H 15.377230 17.170931 16.214734

H 13.987270 17.781136 15.860222

O 12.940234 15.945061 13.320405

H 12.277297 15.275630 13.071022

H 12.433472 16.761585 13.478331

O 13.813875 13.405095 14.326862

H 14.489055 13.051625 13.714765

H 13.761976 12.754829 15.054255

O 15.657980 15.367633 13.429431

H 15.373712 15.894176 12.656391

H 16.522981 15.732033 13.697857

XYZ Coordinates from $Cu(H_2O)_6^{2+}$

Cu 14.468971 15.768163 13.919497

O 15.184625 14.191101 15.500739

H 16.122276 13.962484 15.587459

H 14.712905 13.358798 15.272346

O 13.728246 16.210797 16.623364

H 12.964125 16.099255 16.033204

H 14.279757 15.422618 16.408889

O 15.302203 18.274672 15.847298

H 14.685747 17.565199 16.176659

H 14.706432 18.986492 15.566756

O 12.733376 14.928393 13.992924

H 12.818355 13.945062 14.125491

H 12.172245 15.050822 13.211437

O 13.340927 12.408307 14.595241

H 13.519498 11.782428 13.874508

H 12.756492 11.928663 15.204839

O 15.978137 16.941694 13.740773

H 15.827809 17.557916 14.538254

H 16.800412 16.466589 13.944061

4.3 Molecular structure of the hexaaqua metal complexes, for protocol (ii) calculations using PBE

XYZ Coordinates from $Fe(H_2O)_6^{2+}$

Fe 14.446553 15.529866 14.891286

O 15.821038 14.941369 16.339990

H 16.761715 15.304343 16.377786

H 15.950372 13.944391 16.399418

O 12.986415 15.262260 16.467293

H 12.215191 14.692472 16.170832

H 13.379509 14.746561 17.250548

O 14.736799 17.592343 15.467415

H 14.592375 17.763362 16.465174

H 14.084504 18.246618 15.028754

O 12.734287 16.063653 13.670866

H 12.148703 15.267008 13.488664

H 12.061818 16.774246 13.984868

O 14.278719 13.605821 13.998907

H 15.162903 13.131479 13.931074

H 13.687597 12.963463 14.500270

O 15.849903 15.756772 13.274700

H 15.993155 16.688804 12.896676

H 16.797729 15.500319 13.521463

O 9.486743 16.510191 12.503393

H 9.883004 15.608049 12.688456

H 9.864062 16.673021 11.596937

O 17.078962 18.691471 14.824338

H 16.199516 18.263727 15.103039

H 16.901015 19.651019 14.902387

O 11.726672 14.812975 19.712894

H 12.492061 14.257081 19.313073

H 11.499857 14.386803 20.563512

O 12.659178 11.905310 15.269956

H 12.360842 11.332142 14.486528

H 11.887119 12.491657 15.499076

O 18.637130 18.042042 17.029193

H 17.974597 18.216880 17.759997

H 18.144941 18.331723 16.212800

O 14.697050 15.140698 10.904276

H 15.505101 15.469103 10.413099

H 15.051354 15.116096 11.841182

O 18.264773 13.495201 18.056907

H 17.563899 13.741428 18.724077

H 17.727841 12.932941 17.434553

O 13.152397 17.257697 11.338925

H 12.946597 16.850153 12.244941

H 13.816087 16.565888 11.016682

O 9.586694 12.068726 13.487362

H 10.329063 11.408629 13.334225

H 9.584669 12.180016 14.460705

O 15.625908 11.739463 11.257402

H 16.256807 12.477743 10.917153

H 15.953510 10.934879 10.807622

O 10.938741 16.432372 10.154141

H 10.768570 16.869299 9.296808

H 11.768224 16.881531 10.523285

O 18.784529 13.042472 12.864181

H 19.641223 12.626880 13.080423

H 18.793227 13.967088 13.283898

O 14.046888 11.390526 17.378818

H 13.842819 10.543303 17.819690

H 13.451529 11.446635 16.552654

O 18.379194 15.549677 16.439044

H 18.546703 16.500469 16.797919

H 18.482195 14.817711 17.143447

O 11.156388 17.312537 16.820692

H 10.503071 16.712849 17.280284

H 11.953505 16.714328 16.751495

O 15.834156 16.437306 20.583149

H 16.221195 16.976470 19.823262

H 14.846112 16.578399 20.460516

O 13.255094 16.965508 19.983405

H 12.831715 17.607432 20.587406

H 12.609982 16.187684 19.920867

O 12.316669 19.806178 17.036287

H 12.610983 19.877575 16.090698

H 11.749742 18.986547 17.023345

O 10.943797 17.841232 14.214778

H 10.764312 17.589705 15.170189

H 10.279919 17.383903 13.577931

O 14.365075 19.443670 12.052473

H 13.846061 18.645452 11.679176

H 14.265222 20.158736 11.393278

O 12.919708 11.482658 10.975700

H 13.894452 11.662747 11.106077

H 12.543012 12.358524 10.698022

O 17.500023 13.395083 10.515130

H 18.100520 13.243488 11.297383

H 17.393676 14.379615 10.489235

O 14.286879 18.344672 17.919716

H 13.575145 19.032791 17.658594

H 13.831500 17.758956 18.595448

O 9.856945 15.182728 17.868729

H 10.489585 14.980271 18.633729

H 8.955302 15.108256 18.238220

O 16.464091 12.062759 13.769325

H 17.368672 12.417077 13.511280

H 16.101124 11.737902 12.890220

O 16.535493 18.963909 21.518151

H 15.667155 19.405555 21.608659

H 16.284632 18.002106 21.511065

O 10.779411 13.807035 15.807452

H 10.280495 14.240981 16.573277

H 10.558012 14.324650 15.004050

O 11.820272 10.655852 13.129155

H 11.838560 9.680695 13.074059

H 12.286084 11.001863 12.259969

O 18.400061 15.373109 13.910107

H 18.892936 16.148849 13.576242

H 18.470845 15.411693 14.944745

O 13.739192 13.755387 18.511714

H 13.772444 12.808839 18.159601

H 14.644982 13.890409 18.945176

O 10.936603 14.254603 12.903518

H 11.427239 14.112578 12.030885

H 10.426404 13.391530 13.083501

O 16.735975 18.333491 18.904949

H 16.756800 18.819518 19.782510

H 15.825531 18.522817 18.517002

O 16.636129 18.042422 12.241477

H 16.999960 18.337565 13.129611

H 15.847220 18.651227 12.096450

O 13.108230 19.376169 14.422325

H 12.257990 18.900431 14.185916

H 13.551578 19.588148 13.547683

O 16.384760 12.311708 16.440195

H 15.588263 11.838984 16.819918

H 16.405560 12.056359 15.474764

O 16.116559 14.011888 19.699975

H 16.112142 13.398461 20.462026

H 16.062572 14.946172 20.118846

O 12.205924 14.079126 10.557634

H 13.138475 14.427555 10.641856

H 11.685246 14.847714 10.181784

O 17.086781 16.171392 10.299954

H 17.323317 16.601851 9.455630

H 17.065893 16.908389 10.977074

XYZ Coordinates from $Co(H_2O)_6^{2+}$

Co 14.447447 15.528592 14.892867

O 15.803482 14.978209 16.316716

H 16.750621 15.334379 16.335510

H 15.921779 13.979321 16.388416

O 12.984153 15.239644 16.449945

H 12.218792 14.665435 16.152711

H 13.379685 14.720723 17.226975

O 14.748548 17.581075 15.415157

H 14.588301 17.729313 16.413751

H 14.089295 18.236047 14.988543

O 12.775691 16.071675 13.697282

H 12.194969 15.271370 13.509004

H 12.096204 16.775546 14.016778

O 14.254854 13.614960 14.120999

H 15.132391 13.139681 14.002474

H 13.660813 12.957125 14.597342

O 15.823804 15.764940 13.302431

H 15.978397 16.694685 12.926453

H 16.769754 15.489608 13.530567

O 9.507719 16.505760 12.519756

H 9.918502 15.607703 12.694617

H 9.876362 16.682546 11.612518

O 17.080181 18.707617 14.821143

H 16.198146 18.273274 15.087303

H 16.894209 19.666197 14.891597

O 11.732931 14.792156 19.711402

H 12.496733 14.237553 19.305482

H 11.511649 14.364353 20.562653

O 12.630963 11.865786 15.308465

H 12.357310 11.317284 14.499381

H 11.855419 12.451161 15.527860

O 18.634639 18.058494 17.020102

H 17.968760 18.224948 17.750264

H 18.143584 18.352584 16.204202

O 14.705778 15.136582 10.917379

H 15.516983 15.468583 10.433187

H 15.049704 15.122883 11.858606

O 18.263312 13.526188 18.035525

H 17.567530 13.774609 18.706930

H 17.719770 12.960672 17.421559

O 13.165857 17.262583 11.351325

H 12.967578 16.859990 12.260133

H 13.827365 16.570850 11.026466

O 9.601618 12.097628 13.460466

H 10.339938 11.430800 13.316710

H 9.583373 12.203365 14.434363

O 15.609744 11.748008 11.272217

H 16.250201 12.477238 10.927711

H 15.940525 10.934307 10.841329

O 10.944015 16.458529 10.162458

H 10.777878 16.902991 9.308237

H 11.775934 16.898578 10.536354

O 18.766028 13.014821 12.879554

H 19.615562 12.590854 13.107262

H 18.783666 13.945578 13.284948

O 14.052883 11.361254 17.399222

H 13.867091 10.511778 17.843743

H 13.445276 11.408308 16.582816

O 18.353323 15.573784 16.408127

H 18.531010 16.521644 16.771190

H 18.465581 14.840717 17.110852

O 11.174642 17.286415 16.833209

H 10.516806 16.688597 17.288568

H 11.965479 16.679185 16.749679

O 15.833605 16.442490 20.590887

H 16.223565 16.975708 19.828460

H 14.846070 16.579488 20.459124

O 13.258617 16.947634 19.960748

H 12.826155 17.602680 20.543960

H 12.616913 16.165885 19.912087

O 12.326783 19.784875 17.036978

H 12.611754 19.867517 16.089568

H 11.761094 18.964369 17.022241

O 10.967290 17.824486 14.233283

H 10.784333 17.574692 15.188949

H 10.301794 17.370693 13.594444

O 14.373274 19.459984 12.047578

H 13.858302 18.658197 11.678623

H 14.276535 20.167538 11.379802

O 12.909180 11.504426 10.956826

H 13.885308 11.684124 11.078535

H 12.533218 12.382368 10.684253

O 17.500561 13.379600 10.523713

H 18.099278 13.222282 11.306369

H 17.396652 14.364649 10.504095

O 14.293587 18.296521 17.879744

H 13.587284 18.994682 17.630455

H 13.836552 17.715470 18.558648

O 9.859563 15.163525 17.875486

H 10.493546 14.960998 18.639802

H 8.958538 15.096252 18.247813

O 16.429331 12.078763 13.786250

H 17.336851 12.426664 13.529891

H 16.066803 11.758516 12.904834

O 16.503944 18.991195 21.494253

H 15.629450 19.422601 21.574190

H 16.265889 18.026340 21.501045

O 10.760992 13.783911 15.807547

H 10.271453 14.223201 16.576475

H 10.549568 14.314374 15.010258

O 11.829848 10.671176 13.119607

H 11.837226 9.696565 13.053208

H 12.291102 11.021577 12.250374

O 18.392682 15.365009 13.880652

H 18.877446 16.134029 13.520538

H 18.469210 15.433057 14.912315

O 13.744507 13.737205 18.508860

H 13.781920 12.785729 18.172856

H 14.645868 13.882042 18.948232

O 10.987246 14.268981 12.905752

H 11.469218 14.132856 12.027274

H 10.467301 13.409799 13.076370

O 16.729949 18.322726 18.891770

H 16.736494 18.825280 19.759956

H 15.820251 18.493321 18.491444

O 16.636756 18.040810 12.249454

H 17.005139 18.339852 13.134730

H 15.855222 18.657058 12.099547

O 13.110762 19.383456 14.415183

H 12.259601 18.906397 14.184219

H 13.546507 19.603419 13.539104

O 16.358095 12.350544 16.449930

H 15.578363 11.856614 16.837601

H 16.383622 12.081821 15.488242

O 16.120654 14.021821 19.694998

H 16.133630 13.401815 20.451552

H 16.061535 14.952216 20.121492

O 12.211213 14.104373 10.538510

H 13.141479 14.460608 10.615921

H 11.679287 14.872222 10.176499

O 17.100484 16.158756 10.325724

H 17.339483 16.586095 9.480476

H 17.073666 16.898949 10.999535

XYZ Coordinates from $Cu(H_2O)_6^{2+}$

Cu 14.425498 15.510974 14.875104

O 15.691211 14.950878 16.307209

H 16.641683 15.323995 16.273557

H 15.837079 13.948423 16.335591

O 12.841806 15.286142 16.619680

H 12.124197 14.677417 16.309131

H 13.282421 14.769959 17.363674

O 14.750999 17.450938 15.415931

H 14.578896 17.586682 16.420768

H 14.098674 18.120612 14.984266

O 12.851168 16.013391 13.691914

H 12.292836 15.195120 13.468003

H 12.147397 16.679907 14.057447

O 14.258308 13.673709 14.086636

H 15.158343 13.221456 13.991852

H 13.687236 13.040126 14.631741

O 15.924692 15.722598 13.105839

H 16.070792 16.661764 12.780762

H 16.861296 15.467500 13.361144

O 9.555638 16.453891 12.531675

H 10.005100 15.567859 12.670010

H 9.904317 16.667872 11.624344

O 17.069141 18.644298 14.779599

H 16.220603 18.160546 15.047055

H 16.824160 19.588931 14.859667

O 11.695449 14.788477 19.803035

H 12.465037 14.225230 19.420050

H 11.465416 14.382510 20.662553

O 12.633521 12.024431 15.347862

H 12.371086 11.439258 14.557366

H 11.839581 12.592664 15.545326

O 18.613098 18.036831 16.992014

H 17.951415 18.215823 17.723304

H 18.129577 18.342836 16.176104

O 14.736132 15.172381 10.798507

H 15.519910 15.515700 10.282269

H 15.135927 15.158115 11.726411

O 18.224401 13.535708 17.987015

H 17.546220 13.780539 18.678111

H 17.670924 12.962407 17.389412

O 13.196201 17.251734 11.349953

H 13.030145 16.842688 12.259291

H 13.850892 16.565306 10.991466

O 9.641223 12.194680 13.532812

H 10.355031 11.498614 13.432909

H 9.675501 12.426295 14.487898

O 15.616738 11.791608 11.258535

H 16.253084 12.521453 10.906228

H 15.949445 10.974440 10.835971

O 10.966008 16.470510 10.165235

H 10.798407 16.947490 9.328965

H 11.798053 16.899093 10.553430

O 18.768091 13.018774 12.866666

H 19.602533 12.558425 13.079233

H 18.834946 13.950539 13.266955

O 14.018545 11.389905 17.429729

H 13.826566 10.516992 17.823264

H 13.405937 11.494906 16.623116

O 18.201185 15.577768 16.359196

H 18.420739 16.516377 16.728830

H 18.358957 14.840616 17.052257

O 11.067583 17.283992 16.896497

H 10.396005 16.710815 17.358393

H 11.855685 16.657237 16.865534

O 15.859854 16.450004 20.626466

H 16.241360 16.975950 19.854757

H 14.870796 16.568859 20.493886

O 13.271162 16.913043 19.993211

H 12.851510 17.603473 20.544121

H 12.609662 16.146453 19.972707

O 12.349586 19.719634 17.057966

H 12.623359 19.784235 16.105899

H 11.732748 18.936409 17.058015

O 11.026689 17.697813 14.282558

H 10.795392 17.494246 15.241889

H 10.350067 17.282147 13.628295

O 14.388539 19.446507 12.072195

H 13.882357 18.645954 11.688458

H 14.252415 20.176132 11.435851

O 12.912084 11.516295 11.017734

H 13.890534 11.701011 11.110148

H 12.535635 12.375461 10.695116

O 17.503306 13.421695 10.509992

H 18.098660 13.264813 11.294633

H 17.416368 14.408333 10.466066

O 14.283967 18.169010 17.852076

H 13.582863 18.879687 17.616270

H 13.837811 17.606865 18.555148

O 9.801253 15.125059 17.965476

H 10.453933 14.937234 18.716509

H 8.911394 15.019764 18.355690

O 16.408614 12.164528 13.770721

H 17.331927 12.482513 13.524164

H 16.058446 11.827456 12.889396

O 16.415429 19.063289 21.440669

H 15.521520 19.458666 21.483798

H 16.219686 18.090606 21.487853

O 10.579455 13.800323 15.809672

H 10.166663 14.204223 16.643535

H 10.442717 14.477937 15.115100

O 11.862633 10.728271 13.218185

H 11.900059 9.752707 13.187649

H 12.306685 11.061644 12.335976

O 18.500192 15.384526 13.836030

H 19.014563 16.149374 13.511185

H 18.482634 15.457857 14.865890

O 13.718190 13.726217 18.636945

H 13.755875 12.787889 18.271697

H 14.632308 13.871384 19.044581

O 11.122890 14.261839 12.822997

H 11.554152 14.111008 11.923114

H 10.580814 13.429353 13.040971

O 16.716727 18.300429 18.870621

H 16.695533 18.838475 19.716742

H 15.804023 18.421748 18.458600

O 16.723458 18.104267 12.136170

H 17.058641 18.335332 13.050879

H 15.908187 18.683421 12.041334

O 13.152546 19.270467 14.445678

H 12.297557 18.797986 14.214681

H 13.578480 19.520451 13.571047

O 16.302517 12.355722 16.427066

H 15.525619 11.863333 16.825386

H 16.338149 12.078531 15.467407

O 16.144810 14.026730 19.724911

H 16.194409 13.407986 20.481004

H 16.094336 14.956143 20.153096

O 12.258996 14.102976 10.402774

H 13.188164 14.460759 10.490430

H 11.717879 14.888685 10.098486

O 17.136512 16.194347 10.220266

H 17.432937 16.614997 9.390206

H 17.113014 16.931302 10.897585

XYZ Coordinates from $Ni(H_2O)_6^{2+}$

Ni 14.342427 15.429847 14.886447

O 15.758591 14.849975 16.226822

H 16.666529 15.291328 16.157658

H 15.956528 13.863987 16.208970

O 12.965471 15.300796 16.456682

H 12.178109 14.736926 16.179071

H 13.382109 14.776683 17.221334

O 14.783480 17.419374 15.415685

H 14.614669 17.570722 16.411854

H 14.148782 18.094812 14.983320

O 12.759636 16.021800 13.651325

H 12.177972 15.217901 13.484140

H 12.090433 16.727497 13.987106

O 13.912162 13.546943 14.167813

H 14.731388 13.013112 13.924024

H 13.376632 12.920957 14.740489

O 15.721741 15.455552 13.300150

H 16.083102 16.363358 12.993602

H 16.533264 14.912846 13.504217

O 9.492474 16.519727 12.529858

H 9.879518 15.612948 12.716453

H 9.872981 16.679942 11.624015

O 17.092000 18.644443 14.868374

H 16.232018 18.168943 15.123655

H 16.819177 19.580666 14.783998

O 11.782846 14.800497 19.741069

H 12.547583 14.263849 19.315831

H 11.566759 14.343573 20.578427

O 12.398234 11.778093 15.485708

H 12.194491 11.282880 14.627798

H 11.640577 12.400043 15.668377

O 18.611209 18.152796 17.111505

H 17.952440 18.336010 17.842419

H 18.129899 18.460063 16.295217

O 14.671229 15.045308 10.857210

H 15.489778 15.373308 10.383958

H 14.996362 15.007347 11.806986

O 18.303915 13.561263 17.744040

H 17.629461 13.785979 18.444509

H 17.769789 12.963713 17.157566

O 13.195654 17.200919 11.315371

H 12.992372 16.806213 12.227487

H 13.827314 16.482568 10.984176

O 9.576459 11.998966 13.300634

H 10.360790 11.375471 13.217476

H 9.390792 11.994792 14.261014

O 15.683908 11.745035 10.991118

H 16.385744 12.469989 10.790987

H 16.058336 10.932844 10.595435

O 10.929633 16.456013 10.171427

H 10.756316 16.899421 9.318113

H 11.779784 16.877114 10.525728

O 18.078843 13.988383 13.398619

H 18.485162 13.461252 14.117568

H 18.605206 14.890089 13.466918

O 14.091301 11.404722 17.393173

H 13.948098 10.583737 17.902822

H 13.390789 11.415024 16.655016

O 18.240397 15.740078 16.242097

H 18.394045 16.622834 16.741056

H 18.435144 14.954279 16.856523

O 11.180172 17.351835 16.830864

H 10.527258 16.771418 17.315248

H 11.970537 16.742265 16.760787

O 15.944097 16.410379 20.592884

H 16.310377 17.008117 19.866874

H 14.953135 16.528882 20.476091

O 13.349184 16.918533 20.019943

H 12.945529 17.585122 20.610587

H 12.687671 16.153317 19.981936

O 12.450601 19.782293 17.000235

H 12.753821 19.820602 16.055257

H 11.830797 19.001832 16.996896

O 11.001437 17.817198 14.216713

H 10.810138 17.598577 15.178328

H 10.316880 17.377269 13.588801

O 14.593294 19.244196 12.090879

H 14.012292 18.509817 11.678036

H 14.617717 19.966292 11.432102

O 12.944105 11.503070 10.961658

H 13.923749 11.685804 10.952549

H 12.525828 12.364501 10.700157

O 17.676026 13.412698 10.713417

H 17.977313 13.498453 11.655611

H 17.510255 14.360689 10.461887

O 14.326424 18.186930 17.866216

H 13.645028 18.907693 17.611289

H 13.870173 17.643587 18.576733

O 9.869438 15.269825 17.955190

H 10.519830 15.045144 18.698111

H 8.978452 15.211365 18.352285

O 16.044471 12.094339 13.567894

H 16.740987 12.779233 13.419255

H 15.868973 11.760689 12.628967

O 16.327498 19.003671 21.580936

H 15.407634 19.329834 21.648896

H 16.203675 18.018735 21.569808

O 10.754395 13.871382 15.890757

H 10.249241 14.314580 16.644920

H 10.472416 14.312909 15.061937

O 11.892368 10.675335 13.150395

H 11.896638 9.702771 13.057880

H 12.375723 11.037908 12.304218

O 19.283729 16.165203 13.888626

H 20.245435 15.993306 13.922019

H 18.973125 16.035048 14.852099

O 13.790430 13.794338 18.480988

H 13.830638 12.843008 18.144081

H 14.716934 13.949386 18.858901

O 10.909486 14.245791 12.916581

H 11.380237 14.117172 12.032148

H 10.402781 13.377231 13.071559

O 16.705460 18.415154 18.973005

H 16.626703 18.904164 19.843992

H 15.799054 18.492138 18.534226

O 16.746678 17.679538 12.387837

H 17.150064 18.021148 13.240652

H 15.985769 18.322185 12.224466

O 13.220157 19.276136 14.397743

H 12.354830 18.832198 14.154737

H 13.688894 19.462748 13.531234

O 16.384459 12.220718 16.254904

H 15.590679 11.807655 16.706765

H 16.299816 11.950272 15.302925

O 16.242023 14.053965 19.524093

H 16.274262 13.403170 20.253989

H 16.200985 14.961855 19.996570

O 12.147737 14.075513 10.541180

H 13.085126 14.414599 10.604544

H 11.626486 14.850949 10.180722

O 17.073042 16.063432 10.239796

H 17.299241 16.564823 9.432613

H 17.128238 16.722451 10.993070

XYZ Coordinates from $Fe(H_2O)_6^{3+}$

Fe 14.352010 15.401486 14.917835

O 15.801177 14.859528 16.166964

H 16.744823 15.328214 16.203508

H 15.978009 13.850328 16.208573

O 13.009662 15.197226 16.485819

H 12.157899 14.692800 16.237177

H 13.407306 14.669691 17.284627

O 14.707932 17.370603 15.419783

H 14.579369 17.576701 16.441069

H 14.092607 18.085678 14.967982

O 12.771514 15.950081 13.747656

H 12.171874 15.155513 13.458501

H 12.075998 16.691785 14.037535

O 14.019782 13.519457 14.325389

H 14.787127 12.948850 13.933708

H 13.380117 12.865820 14.796724

O 15.701033 15.454457 13.324901

H 16.060215 16.377816 12.985514

H 16.529041 14.901863 13.492522

O 9.546610 16.499335 12.515013

H 9.967029 15.602435 12.663823

H 9.886624 16.686114 11.597496

O 17.069307 18.639510 14.863737

H 16.224950 18.162991 15.124640

H 16.788904 19.574353 14.783190

O 11.832243 14.807828 19.824966

H 12.567100 14.251752 19.385165

H 11.639406 14.376207 20.681346

O 12.396765 11.844550 15.411746

H 12.173028 11.319181 14.568403

H 11.622394 12.433151 15.628428

O 18.580702 18.125918 17.114380

H 17.939681 18.347729 17.852004

H 18.112819 18.455151 16.299651

O 14.671977 15.055035 10.792361

H 15.505258 15.403413 10.353234

H 14.970668 14.951126 11.736803

O 18.312066 13.570938 17.803268

H 17.618172 13.779491 18.491903

H 17.805165 12.963748 17.206682

O 13.172273 17.168343 11.331635

H 12.979175 16.793745 12.241189

H 13.807085 16.452867 10.994006

O 9.637369 12.151644 13.245113

H 10.358181 11.455469 13.175857

H 9.397595 12.136650 14.193224

O 15.682524 11.740256 10.957992

H 16.387642 12.463406 10.762936

H 16.062998 10.922398 10.579440

O 10.917769 16.450104 10.126517

H 10.742288 16.937297 9.297667

H 11.758923 16.861915 10.504797

O 18.001724 14.030621 13.423788

H 18.341285 13.524415 14.191835

H 18.553656 14.919758 13.496398

O 14.079033 11.402218 17.360717

H 13.959587 10.562500 17.846282

H 13.382884 11.413924 16.626075

O 18.158912 15.730532 16.326640

H 18.322788 16.641406 16.796920

H 18.401650 14.953752 16.945075

O 11.166359 17.344809 16.847864

H 10.539308 16.763803 17.367659

H 11.975503 16.772752 16.798143

O 15.970375 16.414519 20.556896

H 16.343513 17.019918 19.841963

H 14.981109 16.541985 20.433109

O 13.388541 16.943218 19.959106

H 13.000347 17.627652 20.540111

H 12.732498 16.172412 19.971795

O 12.458401 19.781700 17.011120

H 12.736420 19.835323 16.060669

H 11.819764 19.017429 17.006242

O 11.055322 17.723494 14.216874

H 10.817871 17.553582 15.179124

H 10.352063 17.323867 13.568639

O 14.579600 19.212991 12.089233

H 13.989010 18.490913 11.672180

H 14.615294 19.943325 11.439730

O 12.938041 11.491560 10.950069

H 13.917599 11.669076 10.930198

H 12.525309 12.334320 10.630857

O 17.673870 13.415003 10.708653

H 17.966788 13.510741 11.650283

H 17.513253 14.359449 10.443081

O 14.341765 18.178878 17.799064

H 13.650378 18.906390 17.568633

H 13.898394 17.625513 18.515363

O 9.901358 15.283424 18.072190

H 10.554233 15.055699 18.812659

H 9.009975 15.229148 18.469440

O 15.983897 12.095324 13.516959

H 16.683448 12.778804 13.376560

H 15.823860 11.762128 12.567482

O 16.306870 19.007695 21.578383

H 15.382676 19.317935 21.661185

H 16.200936 18.021167 21.565676

O 10.745475 13.936742 15.981073

H 10.267353 14.324741 16.788307

H 10.366642 14.410735 15.212404

O 11.862086 10.687787 13.135288

H 11.860189 9.713657 13.060134

H 12.352556 11.037624 12.287203

O 19.222799 16.182469 13.946007

H 20.187991 16.029126 13.978012

H 18.917374 16.048360 14.903436

O 13.799961 13.773337 18.487892

H 13.827989 12.821137 18.149606

H 14.729588 13.922524 18.875095

O 11.098504 14.279827 12.803604

H 11.506415 14.123724 11.890023

H 10.570987 13.434538 13.025928

O 16.707514 18.461486 18.974848

H 16.606549 18.931205 19.855507

H 15.812924 18.525643 18.519846

O 16.691987 17.624422 12.406239

H 17.119379 17.994907 13.235754

H 15.939199 18.279798 12.233109

O 13.249698 19.226533 14.401731

H 12.375101 18.800533 14.159170

H 13.715982 19.419403 13.531063

O 16.347751 12.294779 16.258057

H 15.563845 11.853720 16.708996

H 16.309348 11.968775 15.322712

O 16.223946 14.042682 19.533413

H 16.262857 13.405061 20.274817

H 16.197759 14.963534 19.988164

O 12.166768 14.059286 10.371682

H 13.101288 14.401843 10.440433

H 11.636316 14.844356 10.048384

O 17.069215 16.066358 10.206032

H 17.283112 16.573374 9.398699

H 17.125833 16.718887 10.961946

XYZ Coordinates from $Co(H_2O)_6^{3+}$

Co 14.330843 15.387071 14.901849

O 15.680424 14.835314 16.133819

H 16.607118 15.321203 16.093126

H 15.881978 13.829684 16.117682

O 13.061333 15.276588 16.367457

H 12.211128 14.756251 16.121527

H 13.466413 14.738152 17.165008

O 14.742915 17.240242 15.410757

H 14.575517 17.415703 16.428170

H 14.126330 17.954765 14.959065

O 12.883135 15.942220 13.714683

H 12.300766 15.129597 13.459258

H 12.169409 16.638391 14.068699

O 13.944657 13.608056 14.290261

H 14.742659 13.048570 13.949997

H 13.389180 12.971884 14.872465

O 15.597596 15.448268 13.410876

H 15.951991 16.388716 13.103000

H 16.439149 14.918777 13.596039

O 9.583899 16.468919 12.540987

H 10.015022 15.573762 12.678951

H 9.907570 16.664075 11.619355

O 17.053198 18.643512 14.902686

H 16.242239 18.116542 15.164304

H 16.718392 19.561229 14.833044

O 11.846559 14.811474 19.804011

H 12.586902 14.295853 19.323148

H 11.669937 14.326104 20.634703

O 12.437643 11.881913 15.468413

H 12.242160 11.362972 14.613732

H 11.649388 12.458390 15.664525

O 18.573893 18.090426 17.127114

H 17.939706 18.310106 17.871051

H 18.115896 18.462022 16.326233

O 14.670711 15.049664 10.805532

H 15.529150 15.385716 10.404660

H 14.919059 14.937057 11.761179

O 18.267236 13.588006 17.706427

H 17.589271 13.807223 18.407258

H 17.749271 12.969067 17.130649

O 13.184733 17.187222 11.297100

H 13.016859 16.816610 12.212705

H 13.816825 16.471616 10.957348

O 9.699333 12.125346 13.254357

H 10.433813 11.442174 13.205931

H 9.445306 12.117232 14.198755

O 15.671595 11.744716 10.946659

H 16.386864 12.459452 10.760788

H 16.061346 10.915200 10.604888

O 10.916079 16.444509 10.132100

H 10.723192 16.928848 9.305424

H 11.759585 16.864057 10.495921

O 17.916481 14.052209 13.446148

H 18.272962 13.566132 14.219675

H 18.480007 14.937563 13.472484

O 14.083513 11.429164 17.420134

H 13.944593 10.619161 17.948725

H 13.385489 11.427341 16.685850

O 18.048353 15.740810 16.230420

H 18.239889 16.625058 16.733142

H 18.302759 14.951234 16.829756

O 11.179339 17.376393 16.871021

H 10.562880 16.775147 17.381586

H 11.997597 16.821580 16.803205

O 15.990368 16.409693 20.619692

H 16.359185 17.014612 19.901334

H 15.001236 16.534374 20.497032

O 13.407678 16.933826 20.014245

H 13.010239 17.634071 20.569366

H 12.740234 16.173393 20.011140

O 12.551949 19.764499 17.016441

H 12.821885 19.784066 16.061479

H 11.869145 19.040978 17.031615

O 11.120284 17.652516 14.234917

H 10.860539 17.520262 15.197431

H 10.411375 17.264952 13.582391

O 14.580718 19.225817 12.087955

H 13.992729 18.518187 11.644774

H 14.642644 19.969924 11.456612

O 12.938087 11.489790 10.950105

H 13.916697 11.672311 10.920333

H 12.519807 12.330881 10.633445

O 17.683601 13.400965 10.727184

H 17.953731 13.487365 11.676037

H 17.526761 14.349617 10.474535

O 14.357494 18.058644 17.790740

H 13.697972 18.814216 17.559640

H 13.909928 17.553682 18.539547

O 9.927566 15.294496 18.052215

H 10.573249 15.060250 18.798094

H 9.030179 15.226652 18.433400

O 15.909795 12.148839 13.510339

H 16.622508 12.818666 13.363047

H 15.753313 11.802953 12.565868

O 16.233612 19.058587 21.560340

H 15.297995 19.338135 21.621467

H 16.164144 18.070091 21.590213

O 10.808612 14.007403 15.942498

H 10.322627 14.386138 16.750629

H 10.399114 14.444090 15.167318

O 11.957829 10.709459 13.186526

H 11.971240 9.734328 13.127313

H 12.410458 11.053529 12.314564

O 19.167342 16.201597 13.885571

H 20.130317 16.037636 13.930016

H 18.847088 16.073031 14.840255

O 13.801491 13.865233 18.388333

H 13.827617 12.896099 18.098034

H 14.728237 14.014744 18.784146

O 11.147802 14.273492 12.819487

H 11.537680 14.124725 11.897584

H 10.627696 13.423672 13.035597

O 16.700457 18.423802 18.986887

H 16.568592 18.928555 19.843315

H 15.812411 18.441726 18.512525

O 16.624259 17.586162 12.484591

H 17.078843 17.979501 13.288547

H 15.900954 18.265547 12.277160

O 13.310415 19.132852 14.427122

H 12.422946 18.731719 14.184586

H 13.757773 19.366279 13.556733

O 16.304180 12.286524 16.212339

H 15.545101 11.842456 16.700640

H 16.242999 11.944884 15.282586

O 16.212385 14.088692 19.470309

H 16.228489 13.409502 20.174906

H 16.199648 14.981962 19.976897

O 12.169588 14.056472 10.363678

H 13.104514 14.397966 10.426654

H 11.636065 14.842834 10.048494

O 17.080707 16.058318 10.288470

H 17.275444 16.594601 9.495290

H 17.103335 16.694834 11.061540

XYZ Coordinates from $Cu(H_2O)_6^{3+}$

Cu 14.334264 15.401168 14.905914

O 15.736184 14.847598 16.205335

H 16.669781 15.315859 16.117411

H 15.929192 13.839912 16.162176

O 13.011534 15.277114 16.435627

H 12.181780 14.736671 16.168520

H 13.435259 14.725662 17.213081

O 14.756176 17.332824 15.424371

H 14.570751 17.494890 16.439043

H 14.117827 18.021976 14.967899

O 12.811892 15.976185 13.694331

H 12.229481 15.158717 13.466118

H 12.112969 16.678598 14.048952

O 13.925074 13.561671 14.208754

H 14.760264 13.018771 13.939612

H 13.377754 12.930566 14.805255

O 15.658118 15.448667 13.360435

H 16.011579 16.390738 13.059214

H 16.492507 14.918299 13.569550

O 9.554060 16.496216 12.522614

H 9.970473 15.597047 12.675937

H 9.898403 16.681316 11.606399

O 17.075202 18.653681 14.883796

H 16.244595 18.153449 15.147777

H 16.771923 19.581556 14.806037

O 11.833919 14.799753 19.801992

H 12.574787 14.271356 19.337270

H 11.646452 14.329435 20.638974

O 12.433471 11.853307 15.441588

H 12.215901 11.329905 14.594465

H 11.652332 12.435179 15.652289

O 18.574000 18.083073 17.115115

H 17.929097 18.302024 17.850510

H 18.124191 18.446326 16.304984

O 14.673548 15.046770 10.806314

H 15.521694 15.383652 10.387006

H 14.945125 14.942130 11.757921

O 18.292511 13.580613 17.737427

H 17.610464 13.797584 18.435104

H 17.776649 12.961181 17.160015

O 13.188476 17.186881 11.298763

H 13.002124 16.811223 12.211551

H 13.821111 16.470226 10.963438

O 9.654722 12.119910 13.266762

H 10.391163 11.439633 13.202837

H 9.417102 12.106082 14.215424

O 15.683141 11.731284 10.984183

H 16.392381 12.448344 10.785236

H 16.064131 10.905658 10.623533

O 10.920652 16.450702 10.130411

H 10.733393 16.924059 9.296153

H 11.765927 16.869797 10.491187

O 17.968452 14.047628 13.452956

H 18.319843 13.567070 14.232111

H 18.530624 14.931791 13.475497

O 14.091588 11.416579 17.390657

H 13.956503 10.599879 17.909974

H 13.395678 11.416011 16.654321

O 18.112764 15.721721 16.237968

H 18.284989 16.617492 16.733444

H 18.351982 14.938840 16.852338

O 11.149446 17.371387 16.855062

H 10.531913 16.774932 17.368697

H 11.967382 16.812925 16.793711

O 15.977710 16.407145 20.587056

H 16.339108 17.011584 19.864706

H 14.986845 16.531061 20.474844

O 13.389722 16.928977 20.009458

H 12.992416 17.613192 20.584362

H 12.729479 16.162112 20.001028

O 12.490445 19.780340 17.014389

H 12.771188 19.812057 16.063217

H 11.826195 19.039233 17.017410

O 11.065202 17.707606 14.226967

H 10.818940 17.545874 15.188846

H 10.366044 17.307859 13.572814

O 14.585319 19.221082 12.097435

H 13.999192 18.507669 11.659080

H 14.642328 19.959123 11.458394

O 12.942298 11.493925 10.966730

H 13.921742 11.673017 10.948017

H 12.526606 12.338927 10.656697

O 17.685595 13.396002 10.733195

H 17.974814 13.485415 11.675756

H 17.525942 14.343364 10.476949

O 14.325300 18.116565 17.811818

H 13.652486 18.858438 17.578169

H 13.879886 17.589740 18.546220

O 9.912981 15.276446 18.044169

H 10.561368 15.046949 18.788825

H 9.017629 15.202013 18.429066

O 15.963384 12.142576 13.538464

H 16.666549 12.820745 13.383920

H 15.805527 11.784937 12.598095

O 16.307905 19.025474 21.562277

H 15.381796 19.329603 21.646359

H 16.210138 18.038437 21.570179

O 10.793104 13.961709 15.952152

H 10.302249 14.349794 16.753156

H 10.407851 14.410022 15.170993

O 11.905945 10.691544 13.168502

H 11.910748 9.717094 13.097772

H 12.379285 11.042168 12.310012

O 19.222669 16.200384 13.887148

H 20.185765 16.035119 13.923623

H 18.909843 16.066438 14.841898

O 13.797491 13.828308 18.413541

H 13.828770 12.866465 18.100617

H 14.725631 13.980122 18.804872

O 11.085706 14.277006 12.832221

H 11.494903 14.127509 11.919222

H 10.565778 13.424689 13.041420

O 16.693602 18.429574 18.966306

H 16.594075 18.921474 19.834575

H 15.795979 18.477895 18.512813

O 16.662903 17.608788 12.458367

H 17.103668 17.992717 13.275422

H 15.921509 18.273889 12.268209

O 13.270907 19.178717 14.419044

H 12.390890 18.762091 14.176916

H 13.726588 19.393163 13.548401

O 16.334137 12.289266 16.231581

H 15.563571 11.845949 16.702094

H 16.279140 11.964576 15.294676

O 16.215402 14.069822 19.479202

H 16.237804 13.406826 20.198888

H 16.195798 14.973901 19.966544

O 12.163232 14.059620 10.395879

H 13.098620 14.400174 10.459351

H 11.633664 14.844275 10.070272

O 17.083899 16.051738 10.266767

H 17.299336 16.566272 9.464580

H 17.120726 16.701856 11.026639

XYZ Coordinates from $Ir(H_2O)_6^{3+}$

Ir 14.333139 15.389412 14.891628

O 15.784279 14.823900 16.231771

H 16.705806 15.307107 16.173133

H 15.989674 13.820662 16.206884

O 13.008133 15.281256 16.479129

H 12.166687 14.751583 16.224445

H 13.424133 14.734718 17.268999

O 14.745706 17.347988 15.494956

H 14.564149 17.527312 16.510731

H 14.119401 18.045084 15.025122

O 12.788588 15.931869 13.613430

H 12.188567 15.120496 13.374310

H 12.094306 16.638303 13.984562

O 13.917991 13.504846 14.168566

H 14.745787 12.950593 13.905592

H 13.344831 12.888406 14.754239

O 15.667186 15.450330 13.295722

H 16.016786 16.399245 12.995849

H 16.509698 14.915686 13.480778

O 9.554342 16.500769 12.490173

H 9.953722 15.592550 12.629139

H 9.902672 16.690455 11.576390

O 17.077979 18.643068 14.882710

H 16.251036 18.156805 15.177778

H 16.781486 19.573114 14.803426

O 11.828193 14.813021 19.838142

H 12.568190 14.280839 19.380127

H 11.637024 14.347389 20.676984

O 12.395668 11.852799 15.439237

H 12.171219 11.315007 14.605107

H 11.623302 12.447939 15.643543

O 18.594563 18.118274 17.121376

H 17.939853 18.326104 17.851078

H 18.138954 18.464622 16.307691

O 14.684410 15.058394 10.724790

H 15.527351 15.410716 10.309357

H 14.962740 14.944608 11.672532

O 18.316911 13.579868 17.762296

H 17.625651 13.786350 18.454086

H 17.817252 12.952003 17.181894

O 13.190036 17.186971 11.248254

H 12.997957 16.787346 12.149571

H 13.826564 16.480175 10.900670

O 9.633216 12.121533 13.247272

H 10.362123 11.432970 13.189801

H 9.402999 12.126708 14.197829

O 15.685454 11.730123 10.986291

H 16.385227 12.457050 10.791865

H 16.064556 10.916370 10.597793

O 10.917063 16.443204 10.092872

H 10.719174 16.913984 9.259518

H 11.764395 16.866960 10.442937

O 17.962731 14.070299 13.457640

H 18.268064 13.604903 14.264891

H 18.543072 14.945860 13.488088

O 14.069569 11.442712 17.388421

H 13.932471 10.627739 17.910093

H 13.371238 11.440487 16.654343

O 18.159518 15.737808 16.263921

H 18.326596 16.634512 16.753221

H 18.401950 14.960848 16.878528

O 11.134663 17.377162 16.866341

H 10.517360 16.782045 17.381783

H 11.958560 16.826364 16.827882

O 15.992370 16.405551 20.586956

H 16.344506 17.012975 19.862289

H 15.000521 16.533700 20.491873

O 13.393603 16.938270 20.052569

H 13.003408 17.613613 20.642654

H 12.732496 16.172522 20.041169

O 12.462385 19.788331 17.025993

H 12.747603 19.815151 16.076660

H 11.800171 19.045135 17.030977

O 11.075585 17.657385 14.227509

H 10.826350 17.498923 15.189103

H 10.363879 17.290133 13.570398

O 14.562029 19.213088 12.104863

H 13.985961 18.500719 11.650120

H 14.603855 19.967931 11.484478

O 12.942992 11.489493 10.978359

H 13.922506 11.667889 10.957112

H 12.529612 12.324296 10.641252

O 17.671191 13.416069 10.744410

H 17.966077 13.506624 11.684709

H 17.518388 14.362446 10.480976

O 14.305419 18.154929 17.863540

H 13.625012 18.886521 17.619792

H 13.867454 17.623961 18.599696

O 9.904659 15.284966 18.072319

H 10.554444 15.059218 18.815977

H 9.010733 15.212465 18.460916

O 15.982044 12.098465 13.544912

H 16.665955 12.795308 13.395770

H 15.832892 11.749939 12.599748

O 16.362011 19.009332 21.575943

H 15.440465 19.322006 21.677101

H 16.254806 18.023156 21.579560

O 10.788660 13.984522 15.973361

H 10.291027 14.358256 16.776457

H 10.397848 14.437191 15.197522

O 11.872549 10.677085 13.166248

H 11.876831 9.703313 13.086791

H 12.359182 11.032789 12.319730

O 19.234773 16.203884 13.899187

H 20.198519 16.040578 13.925165

H 18.933103 16.072743 14.859353

O 13.796669 13.841254 18.445840

H 13.823498 12.882991 18.119178

H 14.728850 13.985444 18.830125

O 11.069313 14.259833 12.759287

H 11.484507 14.095359 11.851831

H 10.554478 13.411688 12.997041

O 16.702193 18.434251 18.971485

H 16.623313 18.918189 19.846782

H 15.798298 18.499281 18.534390

O 16.657581 17.622182 12.444062

H 17.088270 17.994270 13.272712

H 15.913904 18.285949 12.258714

O 13.269546 19.151815 14.436573

H 12.392289 18.727483 14.198242

H 13.723649 19.360833 13.562726

O 16.352978 12.262221 16.253076

H 15.564031 11.840330 16.711234

H 16.296312 11.942999 15.315013

O 16.226581 14.061240 19.492843

H 16.244591 13.407571 20.221172

H 16.210316 14.970483 19.969981

O 12.168085 14.049860 10.333618

H 13.100842 14.396814 10.392905

H 11.632500 14.834791 10.018792

O 17.102301 16.075705 10.237097

H 17.356282 16.580548 9.440037

H 17.132960 16.728060 10.994131

XYZ Coordinates from $Ni(H_2O)_6^{3+}$

Ni 14.396900 15.347977 14.901096

O 15.771662 14.837400 16.020347

H 17.239543 15.534072 16.279524

H 15.881091 13.823494 16.145623

O 12.973567 15.262480 16.439079

H 12.169671 14.707383 16.154257

H 13.380849 14.730151 17.219029

O 14.785254 17.398646 15.416835

H 14.610157 17.549805 16.417022

H 14.131302 18.065196 14.977479

O 12.803365 15.962076 13.687994

H 12.213389 15.163539 13.471795

H 12.123183 16.680071 14.014576

O 13.935602 13.473641 14.139351

H 14.757693 12.921675 13.917227

H 13.375395 12.867259 14.725103

O 15.679148 15.453157 13.215974

H 16.044923 16.385188 12.960470

H 16.501583 14.911909 13.422094

O 9.511317 16.499307 12.538703

H 9.916952 15.595457 12.697602

H 9.864650 16.680054 11.625233

O 17.056775 18.655002 14.918262

H 16.186396 18.165610 15.129602

H 16.794924 19.596743 14.853172

O 11.820748 14.810817 19.762724

H 12.569892 14.277683 19.317519

H 11.616901 14.343638 20.597709

O 12.393169 11.783537 15.449977

H 12.182537 11.265653 14.603750

H 11.628603 12.395864 15.633295

O 18.409754 18.115874 17.053280

H 17.732403 18.319623 17.843266

H 17.927825 18.428609 16.193729

O 14.649426 15.084633 10.741636

H 15.477856 15.421927 10.291147

H 14.961636 15.005837 11.688980

O 18.269142 13.482242 17.835788

H 17.587460 13.710117 18.532152

H 17.729550 12.912061 17.226164

O 13.173399 17.206392 11.321989

H 12.999907 16.797939 12.226694

H 13.798329 16.500624 10.950881

O 9.603043 12.056554 13.251193

H 10.359871 11.399002 13.181317

H 9.378604 12.039023 14.202948

O 15.680910 11.739830 11.016525

H 16.375263 12.473121 10.824204

H 16.044101 10.943084 10.580679

O 10.899912 16.475064 10.161385

H 10.717510 16.952237 9.328239

H 11.745229 16.892065 10.527000

O 18.009165 14.059722 13.467381

H 18.317776 13.573572 14.260742

H 18.540664 14.950243 13.548714

O 14.043277 11.402991 17.401735

H 13.898503 10.589981 17.923927

H 13.353563 11.401823 16.654532

O 18.218671 15.675765 16.485262

H 18.382247 17.065057 16.943093

H 18.399082 14.823322 17.050990

O 11.169906 17.350071 16.842240

H 10.531417 16.756901 17.332165

H 11.970229 16.762119 16.767483

O 15.986777 16.410874 20.573031

H 16.334105 16.954282 19.806418

H 14.993295 16.541508 20.481378

O 13.392516 16.928252 20.033028

H 12.988665 17.600257 20.617662

H 12.729913 16.164242 19.998575

O 12.464048 19.774990 17.014354

H 12.749438 19.811747 16.063655

H 11.825286 19.010776 17.016735

O 11.054195 17.731686 14.216847

H 10.831206 17.550595 15.180298

H 10.351172 17.320417 13.581282

O 14.592899 19.239431 12.107376

H 14.004460 18.515344 11.689389

H 14.617067 19.970626 11.458318

O 12.940388 11.516716 10.970215

H 13.920308 11.697404 10.965686

H 12.524152 12.364732 10.669433

O 17.656596 13.432742 10.760030

H 17.961546 13.531058 11.696797

H 17.495799 14.376225 10.488596

O 14.323028 18.167445 17.852254

H 13.641293 18.896410 17.603210

H 13.878364 17.630676 18.577810

O 9.892959 15.254021 17.984083

H 10.545440 15.036985 18.727090

H 9.002066 15.197247 18.381925

O 16.060040 12.023796 13.587478

H 16.718652 12.742388 13.439334

H 15.887978 11.702462 12.640634

O 16.565110 19.021719 21.415707

H 15.685797 19.422672 21.569864

H 16.373442 18.049165 21.472136

O 10.768973 13.901725 15.897953

H 10.266022 14.314364 16.674146

H 10.432745 14.342390 15.089975

O 11.872969 10.659705 13.139775

H 11.871234 9.688272 13.035815

H 12.359179 11.031777 12.299574

O 19.200035 16.236364 14.036518

H 20.170835 16.118310 14.026434

H 18.942288 16.058696 14.992970

O 13.813087 13.815684 18.430006

H 13.830339 12.853742 18.114456

H 14.730197 13.952374 18.845196

O 10.995319 14.256592 12.860471

H 11.433308 14.124889 11.959379

H 10.481868 13.394984 13.037895

O 16.705533 18.401037 18.849678

H 16.720528 18.885213 19.736707

H 15.766681 18.479861 18.463034

O 16.725014 17.668047 12.393719

H 17.165537 18.008711 13.220624

H 15.974356 18.328316 12.235290

O 13.234459 19.232819 14.416003

H 12.367332 18.794799 14.166803

H 13.704558 19.434790 13.552394

O 16.308528 12.264090 16.287798

H 15.527505 11.821965 16.741683

H 16.281917 11.923753 15.354850

O 16.208943 14.022810 19.559592

H 16.199203 13.372984 20.291436

H 16.194760 14.935533 20.024975

O 12.135044 14.083278 10.439948

H 13.071068 14.427827 10.478203

H 11.601353 14.862213 10.108568

O 17.074846 16.075912 10.209202

H 17.356068 16.560188 9.408754

H 17.127535 16.735122 10.959594

XYZ Coordinates from $Fe(H_2O)_5OH^+$

Fe 14.774924 15.791238 15.280126

O 15.979786 15.682928 16.726575

H 16.743548 16.296451 16.688352

O 13.000848 15.504435 16.600786

H 12.299425 14.910886 16.175189

H 13.360797 14.920805 17.338968

O 14.783793 18.031658 15.458424

H 14.587142 18.171774 16.443424

H 14.038888 18.538495 14.990337

O 13.124352 16.206132 13.745610

H 13.026874 15.233688 13.618617

H 12.216360 16.616763 14.016041

O 14.246156 13.867348 14.377797

H 15.049455 13.361290 14.034939

H 13.661374 13.156490 14.778704

O 16.029161 15.870971 13.475987

H 16.154879 16.769823 13.040368

H 16.977173 15.609475 13.724110

O 9.327966 16.493877 12.584219

H 9.530677 15.543362 12.830573

H 9.836347 16.569417 11.730563

O 17.171828 18.795412 14.759803

H 16.214652 18.553595 15.045298

H 17.242863 19.756029 14.929204

O 11.663673 14.873317 19.626654

H 12.423107 14.282591 19.261723

H 11.430264 14.499435 20.499553

O 12.611302 11.938314 15.285063

H 12.383226 11.435755 14.438073

H 11.850316 12.559476 15.505224

O 18.317383 17.560166 16.912087

H 17.754489 17.974956 17.625361

H 17.982804 17.981530 16.071853

O 14.797753 15.036361 11.244226

H 15.553696 15.337175 10.665082

H 15.210605 15.160457 12.154379

O 17.984864 13.106499 17.738458

H 17.321563 13.437579 18.398327

H 17.436234 12.523097 17.125436

O 13.300407 17.264200 11.363029

H 13.180238 16.940037 12.323270

H 13.951201 16.549441 11.083002

O 9.501152 11.586385 13.277335

H 10.403380 11.162036 13.134988

H 9.366455 11.482144 14.240459

O 15.656883 11.787082 11.063870

H 16.310741 12.509274 10.734615

H 16.066747 10.950911 10.764489

O 11.026897 16.313622 10.430787

H 10.863839 16.561282 9.499732

H 11.867294 16.813031 10.700868

O 18.657558 13.088108 12.846179

H 19.437788 12.562649 13.108306

H 18.745444 13.991937 13.318513

O 14.004349 11.386983 17.355158

H 13.706277 10.588938 17.833425

H 13.403257 11.463831 16.531721

O 19.195025 15.079746 16.484048

H 18.844262 15.962776 16.803990

H 18.657956 14.355474 16.964307

O 11.083058 17.369722 16.874047

H 10.414799 16.744423 17.276765

H 11.910056 16.794470 16.867752

O 15.827612 16.467535 20.218750

H 16.168673 17.049877 19.475498

H 14.831551 16.602620 20.157645

O 13.219627 17.024499 19.845648

H 12.868153 17.589725 20.562124

H 12.570955 16.249302 19.780916

O 12.060202 19.928565 16.922167

H 12.346348 19.970338 15.972601

H 11.557146 19.067968 16.959454

O 11.030566 17.558977 14.227459

H 10.828606 17.440241 15.207078

H 10.277696 17.188261 13.631885

O 14.372056 19.529286 12.040069

H 13.921954 18.705491 11.642138

H 14.301552 20.222416 11.354154

O 12.950804 11.531415 10.809922

H 13.938814 11.674062 10.862487

H 12.584984 12.461869 10.813553

O 17.583764 13.422275 10.396431

H 18.108484 13.284559 11.235856

H 17.453315 14.403422 10.369601

O 14.143200 18.704414 17.926839

H 13.374049 19.300358 17.615994

H 13.718955 18.030015 18.531217

O 9.770966 15.200326 17.773755

H 10.404169 15.002130 18.535841

H 8.872873 15.103509 18.145462

O 16.226223 12.289216 13.631067

H 17.163065 12.598751 13.436158

H 15.931230 11.951303 12.731152

O 16.562944 18.821877 21.433827

H 15.701562 19.255332 21.598612

H 16.297965 17.873505 21.279618

O 11.027057 13.966961 15.735549

H 10.357806 14.297995 16.399022

H 10.696383 14.142494 14.791023

O 12.011868 10.699825 13.034040

H 12.136422 9.730691 13.046066

H 12.448046 11.028741 12.140695

O 18.540074 15.329146 14.091603

H 19.100155 16.063248 13.770502

H 18.806509 15.191468 15.130626

O 13.675332 13.738149 18.517357

H 13.717428 12.813006 18.110921

H 14.569994 13.829795 18.982233

O 10.319680 14.084796 13.219622

H 11.116360 14.061213 12.610978

H 9.932183 13.143239 13.172635

O 16.661327 18.604955 18.747911

H 16.725061 18.913954 19.699501

H 15.730430 18.831028 18.449270

O 16.698632 18.137063 12.214578

H 17.076082 18.455038 13.090507

H 15.888258 18.717161 12.097175

O 12.879350 19.478152 14.308349

H 12.134694 18.839802 14.094679

H 13.352999 19.668316 13.447694

O 16.518013 11.446174 16.225546

H 15.615623 11.387059 16.638633

H 16.346026 11.667960 15.271664

O 16.079554 13.934263 19.625119

H 16.139702 13.461339 20.478713

H 16.074150 14.922861 19.876059

O 12.232181 14.071027 11.282107

H 13.183262 14.375808 11.292027

H 11.751634 14.793218 10.776944

O 17.046594 16.186643 10.316184

H 17.033002 16.642018 9.451938

H 17.042717 16.923669 10.994434

XYZ Coordinates from $Co(H_2O)_5OH^+$

Co 14.703210 15.795133 15.196563

O 15.902686 15.650111 16.656451

H 16.651508 16.276939 16.575789

O 13.017099 15.490841 16.547502

H 12.305706 14.894847 16.143927

H 13.379844 14.926015 17.297700

O 14.774717 17.972589 15.406761

H 14.591104 18.098629 16.397110

H 14.032804 18.503495 14.959630

O 13.054498 16.205382 13.728609

H 12.863963 15.264311 13.525037

H 12.166629 16.660600 14.004317

O 14.322987 13.871931 14.370338

H 15.138601 13.350828 14.090795

H 13.704250 13.178574 14.747441

O 16.027030 15.916844 13.497325

H 16.152532 16.803803 13.040255

H 16.974006 15.660476 13.758665

O 9.321822 16.501742 12.575514

H 9.506820 15.550769 12.830156

H 9.840255 16.566305 11.727080

O 17.168078 18.791363 14.752614

H 16.214888 18.521464 15.017879

H 17.202287 19.754642 14.919153

O 11.668525 14.866747 19.621346

H 12.426605 14.278818 19.246671

H 11.444885 14.490328 20.495690

O 12.639104 11.966007 15.253760

H 12.396668 11.452140 14.417231

H 11.871452 12.571810 15.490287

O 18.328442 17.563373 16.904137

H 17.757416 17.948889 17.626101

H 17.993333 18.005699 16.075964

O 14.800173 15.056948 11.284486

H 15.558473 15.344116 10.700967

H 15.211125 15.187825 12.195176

O 17.962081 13.120001 17.728112

H 17.288018 13.449967 18.377638

H 17.434786 12.497004 17.134381

O 13.312294 17.295611 11.362280

H 13.154708 16.969158 12.315581

H 13.971232 16.579682 11.104598

O 9.470151 11.567787 13.328147

H 10.373319 11.148814 13.173853

H 9.350688 11.466712 14.293635

O 15.663257 11.780914 11.134994

H 16.308407 12.502700 10.787533

H 16.056925 10.946310 10.810445

O 11.048156 16.302102 10.445908

H 10.886479 16.490161 9.500714

H 11.886883 16.817520 10.686968

O 18.699657 13.119983 12.842238

H 19.510274 12.631385 13.082593

H 18.762895 14.031336 13.306952

O 14.017927 11.390611 17.327619

H 13.702066 10.595114 17.798751

H 13.425451 11.480403 16.498466

O 19.167380 15.085739 16.465992

H 18.819151 15.972824 16.784274

H 18.627334 14.361600 16.943706

O 11.097783 17.364135 16.855373

H 10.431578 16.741943 17.265849

H 11.920590 16.784143 16.831004

O 15.812162 16.456484 20.256212

H 16.160666 17.038445 19.513465

H 14.816949 16.587844 20.177763

O 13.214079 17.020599 19.833000

H 12.849571 17.595341 20.535181

H 12.565607 16.244996 19.765280

O 12.095114 19.919593 16.919578

H 12.372283 19.966934 15.967736

H 11.584234 19.063746 16.953573

O 10.998743 17.612361 14.215566

H 10.808248 17.477281 15.195313

H 10.254555 17.218597 13.623774

O 14.399385 19.553615 12.041399

H 13.942845 18.731602 11.647346

H 14.323194 20.247251 11.356504

O 12.955313 11.557900 10.841195

H 13.943975 11.684510 10.921888

H 12.602774 12.493088 10.866216

O 17.571567 13.414616 10.414139

H 18.114052 13.295356 11.245449

H 17.440213 14.394852 10.370075

O 14.165124 18.643300 17.881733

H 13.407391 19.261009 17.586221

H 13.733364 17.986694 18.500925

O 9.776748 15.207543 17.778629

H 10.408722 15.005215 18.540982

H 8.877448 15.107382 18.146447

O 16.313790 12.259118 13.690516

H 17.242103 12.578838 13.472517

H 15.993473 11.929768 12.796437

O 16.542906 18.830060 21.438460

H 15.684704 19.271863 21.597300

H 16.272025 17.881064 21.299321

O 11.012920 13.956659 15.744963

H 10.357750 14.293459 16.420352

H 10.662010 14.131655 14.808450

O 11.979976 10.698081 13.037537

H 12.116734 9.730730 13.055913

H 12.423253 11.031978 12.148794

O 18.533385 15.358109 14.079429

H 19.086516 16.101432 13.767657

H 18.798588 15.208361 15.123432

O 13.669390 13.740003 18.491982

H 13.713453 12.817534 18.080965

H 14.563653 13.831281 18.957751

O 10.260071 14.070792 13.241899

H 11.071444 14.033514 12.657042

H 9.876876 13.126191 13.206564

O 16.655800 18.549138 18.758291

H 16.712075 18.892046 19.698581

H 15.730396 18.774465 18.438285

O 16.725174 18.157719 12.193533

H 17.098541 18.465418 13.074004

H 15.917084 18.741177 12.078089

O 12.897052 19.485348 14.298700

H 12.132970 18.874793 14.077107

H 13.374801 19.680583 13.441204

O 16.560945 11.362265 16.263177

H 15.644661 11.340489 16.649333

H 16.421694 11.599571 15.306722

O 16.064998 13.937864 19.615529

H 16.126401 13.443613 20.456729

H 16.060138 14.921226 19.888631

O 12.256916 14.093630 11.381868

H 13.212458 14.388531 11.362881

H 11.774751 14.804838 10.861042

O 17.045641 16.181511 10.317821

H 16.996290 16.630988 9.451693

H 17.049137 16.923429 10.990933

XYZ Coordinates from $Cu(H_2O)_5OH^+$

Cu 14.531985 15.697414 14.984914

O 15.912196 15.304823 16.225906

H 16.533243 16.063333 16.254517

O 12.861087 15.395806 16.652342

H 12.183166 14.777918 16.278741

H 13.287748 14.846604 17.381218

O 14.692772 17.760059 15.412417

H 14.521084 17.878661 16.410155

H 13.989767 18.362804 14.979312

O 12.885110 16.070603 13.776897

H 12.376218 15.227568 13.570773

H 12.147947 16.709468 14.103417

O 14.374342 13.772485 14.194927

H 15.260056 13.301064 14.183711

H 13.756086 13.149362 14.678584

O 15.953304 15.879404 13.145183

H 16.118149 16.789987 12.764898

H 16.857842 15.621819 13.504242

O 9.536934 16.373986 12.553369

H 10.025938 15.511522 12.712496

H 9.889415 16.593081 11.648870

O 17.043664 18.704895 14.675692

H 16.123561 18.367185 14.959764

H 17.018508 19.660784 14.883138

O 11.671543 14.800044 19.756682

H 12.442343 14.237017 19.368139

H 11.451351 14.398608 20.620728

O 12.672605 12.058843 15.351886

H 12.421678 11.484994 14.552833

H 11.878668 12.624451 15.556163

O 18.197705 17.464563 16.825357

H 17.650351 17.847121 17.566738

H 17.869390 17.924103 16.006076

O 14.749843 15.180247 10.917752

H 15.524148 15.521058 10.384680

H 15.153374 15.209516 11.847013

O 17.989144 13.143911 17.796105

H 17.331417 13.523979 18.433272

H 17.447913 12.472715 17.270655

O 13.182313 17.246283 11.420801

H 13.034779 16.851296 12.344272

H 13.848931 16.565878 11.073312

O 9.677213 12.151431 13.571281

H 10.404577 11.471251 13.458774

H 9.722613 12.385814 14.524760

O 15.664662 11.848188 11.377995

H 16.286354 12.573837 10.993495

H 16.024941 11.020826 11.000038

O 10.982811 16.435150 10.212789

H 10.831244 16.906972 9.370679

H 11.802366 16.872989 10.620161

O 18.807187 13.093213 12.863242

H 19.687185 12.715261 13.052875

H 18.799651 14.035507 13.268259

O 14.008867 11.390004 17.442128

H 13.649216 10.597552 17.886517

H 13.433094 11.528169 16.610743

O 19.123491 15.042048 16.348797

H 18.767910 15.924076 16.673704

H 18.608227 14.331267 16.865360

O 11.015453 17.327179 16.910490

H 10.349788 16.734753 17.356565

H 11.822242 16.723138 16.892840

O 15.795407 16.477725 20.452416

H 16.140421 17.025937 19.679566

H 14.800760 16.600303 20.368464

O 13.189085 16.972402 19.945319

H 12.785797 17.603638 20.573841

H 12.553218 16.185026 19.913477

O 12.135731 19.841649 17.008808

H 12.415821 19.907580 16.058936

H 11.577590 19.015578 17.018931

O 10.961944 17.702967 14.285340

H 10.741830 17.505774 15.247632

H 10.304729 17.245555 13.642253

O 14.299035 19.502173 12.050055

H 13.826048 18.661970 11.708659

H 14.133934 20.185659 11.370609

O 12.984503 11.510966 11.022144

H 13.955546 11.714824 11.152188

H 12.601507 12.372093 10.709725

O 17.523955 13.464759 10.528681

H 18.127420 13.321026 11.312430

H 17.419664 14.448640 10.483374

O 14.143374 18.422527 17.881120

H 13.410790 19.087932 17.621684

H 13.708743 17.808861 18.543613

O 9.773080 15.144057 17.935698

H 10.425922 14.953197 18.686994

H 8.883234 15.004456 18.314739

O 16.508649 12.194579 13.907622

H 17.426554 12.503827 13.642409

H 16.128060 11.908793 13.022916

O 16.567763 18.966239 21.402974

H 15.712256 19.421514 21.537005

H 16.296985 18.009191 21.378435

O 10.638133 13.827006 15.804223

H 10.196315 14.246818 16.613355

H 10.508888 14.468248 15.074483

O 11.924216 10.737867 13.219890

H 11.995475 9.763867 13.210889

H 12.367630 11.067736 12.334955

O 18.456130 15.376323 13.960603

H 19.023303 16.120855 13.679068

H 18.699843 15.201577 14.992261

O 13.687983 13.740555 18.593441

H 13.739903 12.806255 18.208646

H 14.594828 13.872331 19.027081

O 11.163676 14.243367 12.914834

H 11.605733 14.105845 12.016955

H 10.620175 13.406692 13.101192

O 16.615226 18.408650 18.765835

H 16.703669 18.861963 19.655337

H 15.682698 18.608290 18.441988

O 16.703309 18.245919 12.046006

H 17.027298 18.472075 12.968078

H 15.857989 18.782304 11.978676

O 12.951424 19.440560 14.385778

H 12.144874 18.884181 14.167228

H 13.400964 19.643853 13.512546

O 16.594638 11.276284 16.468702

H 15.664076 11.286671 16.817377

H 16.504548 11.530681 15.509725

O 16.091823 14.017363 19.658409

H 16.165178 13.443940 20.446984

H 16.053538 14.970250 20.028748

O 12.301346 14.093204 10.504121

H 13.227568 14.459843 10.603209

H 11.756418 14.867863 10.178609

O 17.103970 16.242201 10.223473

H 17.318594 16.647188 9.360995

H 17.072811 17.005345 10.871850

XYZ Coordinates from $Ir(H_2O)_4(OH)_2^+$

Ir 14.506020 15.568721 14.734687

O 16.412042 16.111376 15.144673

H 16.898336 15.252093 15.125130

O 13.941759 17.432175 15.555713

H 13.092788 17.327729 16.131713

H 14.630777 17.760825 16.244505

O 14.735533 16.508545 12.894159

H 15.009374 17.505488 12.963983

H 13.892887 16.533562 12.286414

O 12.477889 15.061920 14.268959

H 12.004691 14.552625 14.989524

H 11.854877 15.856355 13.969327

O 14.464376 14.616151 16.571253

H 14.576028 13.596176 16.621359

H 13.763627 14.888746 17.253728

O 15.001531 13.759882 13.904944

H 15.127116 13.548734 12.392754

H 15.897446 13.533892 14.270292

O 8.763060 15.900813 14.173596

H 9.064518 15.846805 15.126736

H 8.877029 14.952308 13.886791

O 16.750485 15.573526 11.329232

H 16.005894 15.884053 11.925111

H 17.102561 16.444562 11.000396

O 14.242563 20.606214 16.902815

H 14.660082 19.773793 17.327076

H 14.465638 21.347426 17.501099

O 12.963256 15.454446 18.528722

H 12.276744 14.865149 18.995856

H 12.474645 16.256917 18.176526

O 18.872864 14.870999 12.897691

H 18.887323 15.770977 13.336416

H 18.096999 15.012309 12.278690

O 13.062734 12.070638 14.264895

H 12.586702 12.242918 13.404071

O 18.587509 13.697568 17.203446

H 18.195287 14.513737 17.706191

H 19.553148 13.745138 17.343296

O 11.689827 13.311421 12.319605

H 12.059470 14.029806 12.908099

H 13.852893 12.732015 14.204131

O 9.150330 15.659496 19.057121

H 9.838405 15.013250 19.414708

H 9.505764 16.528244 19.332404

O 13.255131 10.888440 18.778444

H 13.292258 9.985332 18.406706

H 13.895183 11.395953 18.182768

O 9.453264 13.297741 13.694122

H 8.851834 12.552546 13.499360

H 10.177062 13.256298 12.989399

O 17.349530 11.306833 17.310823

H 17.870631 12.160931 17.356310

H 17.476899 10.997278 16.384766

O 15.215145 16.442654 19.365085

H 15.156263 16.727667 20.297899

H 14.331942 15.979388 19.165193

O 17.603114 13.583704 14.761953

H 18.185187 14.025131 14.038527

H 18.087666 13.653271 15.644355

O 12.215103 19.056769 14.116939

H 11.878232 19.556825 14.917993

H 13.004599 18.575653 14.487767

O 18.394196 19.521352 15.349842

H 18.412434 18.719780 14.728790

H 17.501189 19.927095 15.176081

O 15.885384 20.556868 14.770760

H 15.978895 21.473835 14.442858

H 15.236759 20.627621 15.541526

O 13.071777 19.466789 11.569878

H 12.921962 18.539028 11.252873

H 12.623077 19.463823 12.460903

O 10.995602 16.840135 13.302002

H 11.257298 17.733602 13.686857

H 10.045488 16.564721 13.607569

O 13.070041 14.245070 10.282471

H 12.055928 13.553944 11.414632

H 13.027046 13.985251 9.341811

O 10.897161 11.954977 18.211308

H 11.791325 11.503701 18.371323

H 10.976790 12.344130 17.297404

O 14.644681 10.190651 15.242276

H 15.534842 10.466461 14.856329

H 13.985269 10.708382 14.701316

O 15.383119 18.982298 12.673747

H 14.525030 19.284317 12.199986

H 15.506031 19.572123 13.481383

O 11.679801 19.982263 16.570678

H 12.613063 20.316001 16.775263

H 11.067908 20.679832 16.875653

O 14.839685 12.088950 17.017205

H 15.815984 11.884756 17.228471

H 14.665114 11.353210 16.317986

O 17.496992 18.148951 11.355951

H 16.695782 18.628854 11.741099

H 18.050343 18.833327 10.933915

O 11.858972 17.367687 17.085990

H 11.608835 18.337616 17.058428

H 11.070497 16.808570 16.772959

O 11.117095 13.999484 19.673922

H 11.306428 13.712449 20.588430

H 11.005627 13.118063 19.095424

O 17.075260 10.973556 14.398820

H 17.057489 10.919530 13.393897

H 17.263872 11.948252 14.535857

O 15.343844 18.364859 17.534417

H 15.180519 17.723535 18.297637

H 16.365006 18.398478 17.493436

O 9.949249 15.648260 16.579075

H 10.471252 14.814216 16.476875

H 9.555422 15.612114 17.526730

O 18.274523 17.343993 13.861418

H 18.014862 17.652125 12.946914

H 17.427837 16.920987 14.286149

O 15.264454 13.347149 11.379183

H 15.911227 14.058653 11.118043

H 13.955864 13.870587 10.647556

O 12.730889 16.694950 11.298470

H 11.925969 16.633096 11.894971

H 12.792229 15.835720 10.778634

O 17.726026 15.752316 18.474310

H 17.899747 16.626304 18.028759

H 16.796319 15.845860 18.815556

O 17.953144 18.304047 17.526945

H 18.277566 18.876319 18.249955

H 18.198928 18.812606 16.638900

O 11.191489 13.198190 15.765772

H 11.964684 12.699003 15.319036

H 10.453944 13.106014 15.084659

O 16.939392 11.202019 11.716421

H 17.791242 11.636128 11.511872

H 16.277575 11.939767 11.560184

XYZ Coordinates from $Ni(H_2O)_5OH^+$

Ni 14.410400 15.495401 14.869708

O 15.777887 14.924936 16.280381

H 16.711355 15.307307 16.195017

O 13.017883 15.316704 16.446719

H 12.258424 14.724275 16.149939

H 13.436336 14.793509 17.206460

O 14.806626 17.485372 15.403162

H 14.632172 17.631402 16.394387

H 14.162622 18.165314 14.960009

O 12.783109 16.038741 13.651999

H 12.223245 15.234306 13.494835

H 12.102803 16.739002 13.981326

O 14.089123 13.589907 14.127763

H 14.981788 13.144299 13.973692

H 13.563107 12.928856 14.668092

O 15.716921 15.586050 13.277813

H 16.037280 16.502422 12.996188

H 15.938881 13.933942 16.307990

O 9.458297 16.532005 12.573623

H 9.824018 15.618543 12.767153

H 9.849574 16.683352 11.668987

O 17.108130 18.698491 14.838185

H 16.247295 18.219134 15.089736

H 16.848424 19.641830 14.828939

O 11.713127 14.796056 19.599107

H 12.497832 14.251767 19.221074

H 11.469355 14.363518 20.441736

O 12.562447 11.770704 15.382861

H 12.316954 11.266648 14.540451

H 11.805465 12.390475 15.585827

O 18.629647 18.094626 17.041944

H 17.972361 18.256740 17.778542

H 18.140056 18.415142 16.233544

O 14.511730 15.260019 10.894507

H 15.362545 15.494783 10.427309

H 15.210129 15.299629 12.457846

O 18.292744 13.572759 17.902097

H 17.603719 13.827090 18.576983

H 17.752038 12.983332 17.308701

O 13.087207 17.390173 11.318967

H 12.939292 16.939155 12.199401

H 14.040375 16.162876 10.972195

O 9.537601 11.938260 13.358818

H 10.343400 11.346619 13.237698

H 9.437839 11.974845 14.331528

O 15.604502 11.816494 11.185570

H 16.246349 12.548982 10.836885

H 15.943353 10.998829 10.769099

O 10.903841 16.516638 10.235559

H 10.684034 16.923739 9.375430

H 11.735361 17.007685 10.569050

O 18.555485 13.469308 12.931966

H 19.346317 12.936483 13.144352

H 18.776703 14.427448 13.254075

O 14.110654 11.367019 17.392788

H 13.936952 10.542613 17.886629

H 13.464826 11.379614 16.604224

O 18.299074 15.641458 16.262374

H 18.455500 16.557490 16.696791

H 18.450360 14.899256 16.941801

O 11.229216 17.365370 16.822550

H 10.551607 16.760747 17.238523

H 12.016481 16.751609 16.745280

O 15.824262 16.440682 20.597586

H 16.222963 16.993803 19.852703

H 14.837975 16.561569 20.447000

O 13.247153 16.935820 19.938020

H 12.806640 17.580981 20.526073

H 12.598469 16.162792 19.853513

O 12.478839 19.830489 16.909383

H 12.794427 19.828992 15.962179

H 11.862809 19.049673 16.931130

O 11.026767 17.830644 14.207892

H 10.857958 17.619320 15.175436

H 10.321008 17.387057 13.608642

O 14.497791 19.264611 12.151079

H 13.656146 18.264102 11.585358

H 14.535413 20.070304 11.601879

O 12.897803 11.546968 10.931318

H 13.875634 11.741356 11.010517

H 12.487141 12.420560 10.696375

O 17.459046 13.479533 10.472792

H 17.982405 13.448645 11.325434

H 17.298178 14.453966 10.358202

O 14.328519 18.257795 17.860463

H 13.661417 18.979917 17.573238

H 13.838051 17.711210 18.543732

O 9.850069 15.235463 17.744338

H 10.474716 14.998649 18.504130

H 8.947715 15.183266 18.115520

O 16.333819 12.234975 13.713689

H 17.158187 12.773139 13.494886

H 16.047112 11.881993 12.816870

O 16.348047 19.024299 21.516042

H 15.451301 19.415084 21.535181

H 16.154206 18.049875 21.519853

O 10.896811 13.820257 15.757466

H 10.304606 14.289856 16.421501

H 10.729020 14.186892 14.854732

O 11.866330 10.667705 13.098366

H 11.880686 9.695540 13.002037

H 12.324896 11.042341 12.238033

O 19.074642 15.837492 13.787514

H 20.045149 15.949036 13.799529

H 18.828871 15.732065 14.771006

O 13.783055 13.762043 18.472242

H 13.833385 12.812633 18.132660

H 14.685954 13.913703 18.903883

O 10.800462 14.215676 12.984763

H 11.335791 14.119527 12.128064

H 10.279908 13.343139 13.073805

O 16.727807 18.339090 18.930402

H 16.687526 18.853193 19.789619

H 15.824019 18.474789 18.499042

O 16.621652 17.878843 12.276954

H 17.065943 18.194547 13.109728

H 15.773786 18.495740 12.194446

O 13.288204 19.301509 14.355424

H 12.420698 18.864319 14.132344

H 13.793347 19.414124 13.431903

O 16.419204 12.287653 16.370421

H 15.629923 11.824232 16.773609

H 16.393713 12.065812 15.396467

O 16.179530 14.055447 19.622896

H 16.218439 13.415038 20.361327

H 16.101281 14.969494 20.078435

O 12.096251 14.130273 10.662356

H 13.045366 14.458773 10.700591

H 11.589932 14.911589 10.289391

O 16.959499 16.180528 10.271037

H 17.129825 16.686947 9.454002

H 16.970589 16.860793 11.020416

XYZ Coordinates from $Fe(H_2O)_5OH^{2+}$

Fe 14.455523 15.416897 15.029363

O 15.802230 15.066344 16.172892

H 16.765575 15.399106 16.169199

O 12.984708 15.241667 16.505052

H 12.162757 14.715211 16.220476

H 13.364644 14.714389 17.304864

O 14.691079 17.503369 15.378462

H 14.581359 17.683803 16.388928

H 14.048650 18.181815 14.944440

O 12.811183 16.012274 13.746324

H 12.240490 15.207089 13.537453

H 12.117212 16.722936 14.036105

O 13.976459 13.567297 14.224882

H 14.787268 12.994643 13.993121

H 13.360163 12.936832 14.724228

O 15.730381 15.481796 13.307128

H 16.074867 16.401825 12.986481

H 16.559605 14.945499 13.483663

O 9.497910 16.486829 12.560225

H 9.913173 15.590012 12.737090

H 9.858017 16.657881 11.647898

O 17.090855 18.628524 14.874003

H 16.209173 18.193259 15.105352

H 16.868663 19.580503 14.821353

O 11.760381 14.800051 19.750073

H 12.510113 14.238415 19.339108

H 11.552275 14.386055 20.611362

O 12.401850 11.824833 15.406059

H 12.181536 11.308152 14.561577

H 11.629217 12.414874 15.622041

O 18.637886 18.101268 17.116324

H 17.984220 18.288526 17.849725

H 18.143621 18.393938 16.303516

O 14.659593 15.042842 10.859941

H 15.482449 15.379459 10.397735

H 14.974657 14.987503 11.808582

O 18.183244 13.502009 17.637232

H 17.523529 13.747915 18.340505

H 17.654789 12.859241 17.071910

O 13.177928 17.182932 11.359666

H 13.003388 16.799818 12.277813

H 13.807957 16.469278 11.014920

O 9.575716 12.047851 13.269355

H 10.337124 11.398295 13.176463

H 9.368568 12.011791 14.224567

O 15.684035 11.752345 11.064940

H 16.378551 12.479323 10.845947

H 16.063430 10.936335 10.681648

O 10.914242 16.444872 10.195388

H 10.746984 16.909045 9.351897

H 11.756312 16.863620 10.568385

O 18.063635 14.036959 13.426098

H 18.433315 13.546334 14.189640

H 18.612123 14.927960 13.460339

O 14.124982 11.438474 17.324834

H 13.915227 10.639122 17.846892

H 13.442311 11.460284 16.576334

O 18.329808 15.686449 16.228970

H 18.472126 16.549562 16.759373

H 18.420974 14.854222 16.822994

O 11.180388 17.367120 16.853082

H 10.532332 16.779446 17.337553

H 11.978261 16.775907 16.791555

O 15.892911 16.415169 20.555112

H 16.279341 17.007373 19.834880

H 14.906129 16.540565 20.413113

O 13.317320 16.943476 19.912538

H 12.915079 17.613818 20.500184

H 12.663711 16.171174 19.900505

O 12.419713 19.824945 17.003695

H 12.685485 19.881606 16.049220

H 11.808967 19.037903 17.010888

O 11.016694 17.780717 14.221915

H 10.814384 17.586118 15.185768

H 10.324624 17.339718 13.597352

O 14.555042 19.258617 12.085578

H 13.981764 18.509537 11.689960

H 14.582262 19.959795 11.404468

O 12.948249 11.493140 10.947479

H 13.927036 11.680293 10.971077

H 12.536201 12.352075 10.668729

O 17.666230 13.419914 10.744413

H 17.971518 13.512827 11.683865

H 17.502889 14.365523 10.484412

O 14.340615 18.264536 17.816350

H 13.646301 18.982092 17.580462

H 13.881767 17.686948 18.499095

O 9.849400 15.294369 17.971200

H 10.491671 15.048124 18.713981

H 8.954800 15.252872 18.362330

O 16.044551 12.098568 13.642386

H 16.736349 12.789795 13.507656

H 15.881986 11.778905 12.693668

O 16.316622 18.987922 21.566494

H 15.404524 19.338094 21.618149

H 16.166789 18.006453 21.544176

O 10.764853 13.920637 15.915128

H 10.234343 14.346259 16.663600

H 10.477627 14.355577 15.084352

O 11.856040 10.672171 13.115136

H 11.865605 9.698182 13.039644

H 12.350332 11.025611 12.269553

O 19.309902 16.197664 13.863602

H 20.273373 16.032453 13.856272

H 19.035027 16.036280 14.831568

O 13.765770 13.763867 18.493498

H 13.833547 12.829239 18.103665

H 14.691517 13.922591 18.881720

O 10.981135 14.256700 12.941183

H 11.436320 14.127995 12.047213

H 10.456965 13.396991 13.090174

O 16.721384 18.412571 18.965093

H 16.646419 18.893445 19.841723

H 15.827735 18.529000 18.515083

O 16.707713 17.696083 12.390838

H 17.121593 18.044459 13.237082

H 15.946763 18.340835 12.228804

O 13.151158 19.343965 14.369482

H 12.295063 18.876367 14.134222

H 13.631491 19.510601 13.504402

O 16.713148 11.624495 16.315147

H 15.805360 11.555943 16.714031

H 16.519975 11.669070 15.346470

O 16.196295 14.046601 19.519734

H 16.262415 13.400018 20.250879

H 16.153880 14.958212 19.984896

O 12.144943 14.065896 10.539867

H 13.083450 14.403571 10.593348

H 11.623009 14.840339 10.179094

O 17.053281 16.069409 10.242253

H 17.264921 16.561630 9.425406

H 17.105960 16.739341 10.984465

XYZ Coordinates from $Co(H_2O)_5OH^{2+}$

Co 14.313228 15.373288 14.846346

O 15.615986 14.687447 15.934752

H 16.358042 15.346310 15.975593

O 13.059410 15.270392 16.321419

H 12.218134 14.742333 16.092838

H 13.494281 14.741933 17.100316

O 14.778794 17.199690 15.386747

H 14.630281 17.342673 16.401466

H 14.162221 17.918471 14.963657

O 12.801469 15.999427 13.648594

H 12.204044 15.203856 13.434758

H 12.129161 16.703320 14.001553

O 13.883834 13.617563 14.180872

H 14.743986 13.074730 14.027956

H 13.333480 13.008493 14.781815

O 15.594280 15.434537 13.377499

H 15.971120 16.364924 13.091136

H 16.398274 14.907867 13.660041

O 9.534867 16.554470 12.516121

H 9.927179 15.645474 12.677616

H 9.890711 16.725159 11.602128

O 17.072210 18.659651 14.916766

H 16.284637 18.098241 15.176448

H 16.686876 19.554615 14.819459

O 11.840393 14.785677 19.758611

H 12.590046 14.280314 19.269861

H 11.655095 14.278926 20.574313

O 12.430397 11.867091 15.463163

H 12.222531 11.349218 14.615003

H 11.643355 12.441822 15.666997

O 18.658933 18.277199 17.171003

H 18.009308 18.421639 17.915917

H 18.149141 18.579385 16.373899

O 14.656186 15.031363 10.820727

H 15.510676 15.361919 10.409948

H 14.915200 14.933347 11.777408

O 18.097453 13.641037 17.463152

H 17.469801 13.859517 18.201706

H 17.586972 12.937340 16.956591

O 13.195178 17.202833 11.283987

H 13.003488 16.816643 12.196607

H 13.822355 16.483281 10.948990

O 9.612276 12.096733 13.292747

H 10.372559 11.442736 13.223955

H 9.398015 12.092178 14.246955

O 15.669433 11.759505 11.132711

H 16.381077 12.467912 10.901292

H 16.046700 10.923289 10.793371

O 10.921130 16.474891 10.130127

H 10.742709 16.940867 9.289861

H 11.770822 16.889146 10.489062

O 18.003760 14.018992 13.456739

H 18.475656 13.532136 14.164028

H 18.483886 14.954170 13.477262

O 14.159194 11.464092 17.379814

H 13.908934 10.712319 17.952293

H 13.472546 11.477215 16.636826

O 18.073566 15.922570 16.150139

H 18.324750 16.718886 16.722049

H 18.196304 15.053599 16.669635

O 11.226400 17.364131 16.842933

H 10.602516 16.768448 17.350424

H 12.024974 16.783146 16.736217

O 15.984926 16.421311 20.712937

H 16.371736 17.019609 19.998115

H 14.999326 16.533525 20.558956

O 13.408598 16.894900 20.027557

H 12.982743 17.610816 20.539621

H 12.741315 16.133668 20.010122

O 12.633904 19.711858 17.051111

H 12.897819 19.743629 16.094194

H 11.950844 18.987008 17.058052

O 11.061242 17.785786 14.219124

H 10.839337 17.587423 15.177439

H 10.366377 17.376104 13.576288

O 14.598167 19.224613 12.101183

H 14.008834 18.516283 11.657205

H 14.656693 19.969617 11.470789

O 12.944976 11.512762 10.979018

H 13.923730 11.703606 11.002812

H 12.529585 12.356069 10.664681

O 17.677150 13.382613 10.789438

H 17.953974 13.492431 11.738128

H 17.521728 14.324821 10.514693

O 14.427391 17.976380 17.805151

H 13.781108 18.742047 17.577220

H 13.965035 17.477238 18.547105

O 9.930341 15.302660 18.006830

H 10.575636 15.054698 18.749119

H 9.035276 15.261857 18.396958

O 15.967888 12.169724 13.713988

H 16.693037 12.834416 13.597094

H 15.827168 11.837449 12.766288

O 16.077168 19.113370 21.562848

H 15.124308 19.332910 21.527307

H 16.063143 18.124897 21.632324

O 10.792851 13.970572 15.908230

H 10.302891 14.381849 16.694788

H 10.428449 14.400361 15.106835

O 11.889274 10.714334 13.172750

H 11.892029 9.740016 13.100438

H 12.365295 11.062326 12.314520

O 19.094170 16.269904 13.813118

H 20.061472 16.143301 13.871826

H 18.766042 16.180985 14.786548

O 13.803719 13.872585 18.371374

H 13.880045 12.913889 18.045596

H 14.722361 14.048591 18.770063

O 10.990126 14.294752 12.842976

H 11.427927 14.141556 11.944734

H 10.481971 13.435503 13.045202

O 16.735951 18.392044 19.043897

H 16.544559 18.929554 19.867258

H 15.866423 18.368877 18.533256

O 16.651107 17.586561 12.478193

H 17.118863 17.962799 13.280212

H 15.925908 18.266468 12.286205

O 13.334909 19.133036 14.444349

H 12.434375 18.764977 14.197348

H 13.785391 19.367630 13.576938

O 16.747972 11.548753 16.322734

H 15.855336 11.513168 16.755844

H 16.507591 11.654995 15.368810

O 16.206914 14.143434 19.454185

H 16.264128 13.427534 20.118493

H 16.198054 15.005669 20.005206

O 12.135670 14.075308 10.434706

H 13.073787 14.409931 10.497508

H 11.616498 14.859690 10.092596

O 17.073227 16.033740 10.299363

H 17.283491 16.560494 9.503899

H 17.100762 16.677611 11.067139

XYZ Coordinates from $Ir(H_2O)_5OH^{2+}$

Ir 14.388098 15.367174 14.882646

O 15.810947 14.818808 16.262974

H 16.722808 15.314316 16.211897

H 16.032317 13.819621 16.245548

O 13.043531 15.276927 16.469803

H 12.208923 14.743788 16.204134

H 13.448310 14.734128 17.262073

O 14.808416 17.322275 15.470159

H 14.627193 17.489268 16.474181

H 14.171368 18.063058 14.985961

O 12.888416 15.897258 13.547885

H 12.276659 15.095078 13.399644

H 12.202012 16.631401 13.895768

O 13.961886 13.474137 14.173613

H 14.799987 12.924977 13.933714

H 13.393842 12.864895 14.765254

O 15.706627 15.408803 13.296901

H 16.012389 16.379820 12.979917

H 16.546346 14.906402 13.496982

O 9.499081 16.485072 12.614134

H 9.898397 15.576289 12.754663

H 9.825247 16.667991 11.687430

O 17.097195 18.665347 14.897867

H 16.287447 18.137527 15.174487

H 16.748409 19.574723 14.798463

O 11.761932 14.823163 19.740542

H 12.521262 14.286240 19.320838

H 11.536197 14.363720 20.574183

O 12.440502 11.811024 15.453291

H 12.222915 11.290651 14.606697

H 11.673071 12.415965 15.649684

O 18.592360 18.159212 17.142506

H 17.945899 18.340201 17.885872

H 18.117574 18.505032 16.337623

O 14.297489 15.450089 9.834273

H 15.254897 15.492664 10.096502

O 18.333340 13.627055 17.830300

H 17.627837 13.842713 18.503929

H 17.848593 12.988680 17.248519

O 13.081313 17.379897 11.204662

H 13.087195 16.786782 12.002047

H 13.929807 16.292193 10.236564

O 9.625453 12.022261 13.262562

H 10.388505 11.371953 13.195518

H 9.412269 12.015640 14.217168

O 15.650893 11.750019 11.072148

H 16.377754 12.456963 10.861800

H 15.973912 10.932634 10.642821

O 10.800524 16.553079 10.221823

H 10.532582 17.010758 9.401526

H 11.634265 17.035759 10.540273

O 18.082034 13.970262 13.455725

H 18.458903 13.476260 14.213346

H 18.597378 14.877666 13.507690

O 14.112251 11.431640 17.407278

H 13.973828 10.619774 17.933247

H 13.422627 11.418706 16.664547

O 18.177406 15.766728 16.298654

H 18.342413 16.670272 16.774377

H 18.424216 14.999173 16.921381

O 11.228206 17.388877 16.829616

H 10.568041 16.802711 17.299980

H 12.036389 16.812094 16.817194

O 15.912705 16.404849 20.650307

H 16.296903 16.995758 19.927771

H 14.925650 16.533308 20.516280

O 13.332597 16.941225 20.024491

H 12.921863 17.618786 20.597685

H 12.667235 16.180670 19.982439

O 12.569025 19.801498 16.938341

H 12.887242 19.772870 15.994652

H 11.900080 19.066050 16.957165

O 11.190745 17.629818 14.184259

H 10.978594 17.486536 15.158307

H 10.427881 17.264298 13.583138

O 14.581783 19.153195 12.223579

H 13.641405 18.199809 11.520385

H 14.755862 20.002572 11.775869

O 12.915501 11.609493 10.982736

H 13.898209 11.776904 10.991079

H 12.509697 12.471662 10.701032

O 17.630549 13.393342 10.769776

H 17.985812 13.436875 11.693621

H 17.399064 14.351311 10.597202

O 14.349707 18.141289 17.862699

H 13.698059 18.886664 17.589643

H 13.874209 17.627186 18.585067

O 9.879778 15.317585 17.926962

H 10.508192 15.076728 18.683259

H 8.977132 15.273230 18.298942

O 16.056085 12.089572 13.614490

H 16.734802 12.795072 13.463301

H 15.886297 11.746829 12.670219

O 16.282228 19.020328 21.608520

H 15.361351 19.346882 21.659223

H 16.159867 18.035755 21.618124

O 10.853236 13.961374 15.899019

H 10.296138 14.376928 16.636522

H 10.553538 14.366363 15.056703

O 11.924815 10.682437 13.153470

H 11.965348 9.712344 13.045225

H 12.376553 11.084103 12.303682

O 19.224610 16.187412 13.915380

H 20.194091 16.067264 13.954320

H 18.919772 16.062802 14.875677

O 13.794725 13.830854 18.457804

H 13.833964 12.874637 18.128537

H 14.719211 13.981083 18.855748

O 10.999522 14.238654 12.896811

H 11.441302 14.148977 11.985738

H 10.500930 13.364116 13.053254

O 16.712123 18.387442 19.024234

H 16.614511 18.892915 19.884502

H 15.817047 18.456141 18.564452

O 16.520945 17.616676 12.445936

H 17.027186 17.982493 13.222264

H 15.704338 18.326301 12.313049

O 13.425685 19.109729 14.413673

H 12.543190 18.705800 14.180167

H 13.930661 19.251583 13.464366

O 16.412104 12.266598 16.307298

H 15.619570 11.844447 16.757788

H 16.359384 11.950536 15.366994

O 16.209785 14.071682 19.541190

H 16.239102 13.397702 20.250258

H 16.161209 14.966274 20.041453

O 12.112658 14.173470 10.505210

H 13.045372 14.555136 10.350783

H 11.521549 14.903281 10.166991

O 16.944480 15.981354 10.406229

H 17.272625 16.445389 9.611877

H 16.931923 16.675489 11.137437

XYZ Coordinates from $Cu(H_2O)_6^+$

Cu 14.479819 15.346527 14.853349

O 15.654547 14.924353 16.422979

H 16.580868 15.307259 16.350692

H 15.836543 13.937437 16.413230

O 12.730484 15.392992 16.856713

H 12.190231 14.727077 16.370998

H 13.251970 14.835723 17.502627

O 14.984650 18.006587 15.452924

H 14.740771 18.015892 16.425776

H 14.239048 18.540045 15.037536

O 12.849737 16.008862 13.747655

H 12.288659 15.209735 13.545853

H 12.167874 16.682518 14.106231

O 14.173753 13.396032 13.689473

H 15.093928 13.031650 13.745551

H 13.665395 12.840684 14.340541

O 15.986960 15.666790 13.077440

H 16.135680 16.621118 12.804081

H 16.911908 15.391046 13.342427

O 9.522387 16.509643 12.577888

H 9.928470 15.606224 12.742585

H 9.895677 16.690971 11.673857

O 17.301124 18.827697 14.716172

H 16.361610 18.541165 15.053554

H 17.267540 19.805300 14.729336

O 11.629833 14.781315 19.724506

H 12.407123 14.196442 19.398418

H 11.359118 14.406961 20.586513

O 12.642804 11.858686 15.342571

H 12.334781 11.291386 14.564225

H 11.889683 12.479840 15.548823

O 18.631616 18.049600 16.998719

H 17.930807 18.244957 17.686182

H 18.221854 18.389534 16.153513

O 14.722991 15.159724 10.847128

H 15.491866 15.501485 10.311319

H 15.153429 15.143226 11.768340

O 18.216020 13.508837 17.982865

H 17.534444 13.739913 18.674513

H 17.668026 12.949112 17.365922

O 13.192585 17.228520 11.442172

H 13.027808 16.801117 12.353677

H 13.846719 16.547345 11.072336

O 9.558211 12.093751 13.552275

H 10.300328 11.436268 13.397804

H 9.662198 12.330462 14.501707

O 15.649369 11.709808 11.213678

H 16.260434 12.471636 10.896247

H 15.982269 10.932877 10.721659

O 10.980513 16.460345 10.236179

H 10.844267 16.924881 9.387486

H 11.812767 16.880825 10.639000

O 18.846180 12.964593 12.805417

H 19.711945 12.538590 12.953708

H 18.888180 13.877695 13.250530

O 14.068442 11.349945 17.412727

H 13.900631 10.477073 17.817156

H 13.453922 11.419080 16.598647

O 18.220489 15.576737 16.365717

H 18.441961 16.514431 16.725072

H 18.364435 14.845676 17.062141

O 10.984018 17.364118 16.939329

H 10.276482 16.783451 17.327407

H 11.764533 16.721207 16.970872

O 15.793881 16.452694 20.490093

H 16.162346 16.995733 19.724350

H 14.800398 16.571502 20.385322

O 13.185455 16.930833 19.978112

H 12.782145 17.546553 20.621729

H 12.539193 16.154642 19.912537

O 12.187193 19.852572 17.039386

H 12.477736 19.922964 16.092558

H 11.612160 19.039211 17.042350

O 11.035871 17.752814 14.315593

H 10.798182 17.558532 15.275064

H 10.357124 17.336316 13.671131

O 14.381508 19.447191 12.084877

H 13.879333 18.619059 11.753445

H 14.278735 20.110516 11.374014

O 12.920832 11.476963 11.007619

H 13.900395 11.647175 11.109587

H 12.551554 12.356350 10.732183

O 17.495216 13.425484 10.509473

H 18.111566 13.260639 11.276536

H 17.397099 14.411142 10.481703

O 14.191876 18.444353 17.971283

H 13.462784 19.095877 17.681819

H 13.739654 17.818952 18.606723

O 9.761283 15.102645 17.826554

H 10.416835 14.926187 18.575772

H 8.881037 14.905648 18.203008

O 16.544772 11.940096 13.739370

H 17.439449 12.306222 13.473235

H 16.156618 11.657097 12.860293

O 16.528725 18.942214 21.465670

H 15.666362 19.393699 21.563879

H 16.262942 17.984205 21.427035

O 10.673387 13.691586 15.750878

H 10.223627 14.181491 16.512481

H 10.657165 14.294961 14.975475

O 11.790143 10.654337 13.163391

H 11.778155 9.681038 13.082033

H 12.272633 11.002660 12.309473

O 18.543516 15.290437 13.865008

H 19.047225 16.060485 13.536272

H 18.496456 15.392809 14.896366

O 13.699708 13.642435 18.707128

H 13.756325 12.731121 18.283425

H 14.612017 13.784533 19.111729

O 10.950068 14.248581 12.929497

H 11.467248 14.117941 12.071635

H 10.412343 13.397002 13.068964

O 16.673212 18.378195 18.826938

H 16.727797 18.834338 19.718129

H 15.746318 18.586429 18.485969

O 16.728126 18.096119 12.182710

H 17.108645 18.373800 13.071525

H 15.906441 18.666224 12.095663

O 13.011569 19.528569 14.415135

H 12.239472 18.920701 14.214721

H 13.466748 19.689414 13.539329

O 16.345846 12.310812 16.383206

H 15.572130 11.819069 16.785341

H 16.367322 12.055225 15.416228

O 16.153082 13.984993 19.752684

H 16.236001 13.435427 20.557325

H 16.074951 14.944479 20.101150

O 12.236584 14.090613 10.586521

H 13.172088 14.441188 10.646397

H 11.708410 14.862723 10.229947

O 17.116613 16.205835 10.236805

H 17.402088 16.638423 9.409206

H 17.093499 16.935083 10.922979

4.4 Molecular structure of the hexaaqua metal complexes, for protocol (ii) calculations using R2SCAN

XYZ Coordinates from $Fe(H_2O)_6^{2+}$

Fe 14.380314 15.440098 14.871484

O 15.870579 14.864930 16.207607

H 16.774049 15.285788 16.162290

H 16.030932 13.886624 16.272053

O 12.980480 15.218155 16.470354

H 12.173674 14.705871 16.189806

H 13.367170 14.696468 17.237226

O 14.745504 17.469793 15.450110

H 14.611415 17.640004 16.434689

H 14.137421 18.145606 15.012318

O 12.679260 16.026776 13.691328

H 12.080378 15.249765 13.515600

H 12.046986 16.762345 13.973028

O 14.006420 13.513025 14.059971

H 14.812833 12.963318 13.868045

H 13.417638 12.910297 14.587932

O 15.793996 15.491112 13.268582

H 16.132495 16.387026 12.948466

H 16.608588 14.958743 13.475141

O 9.463576 16.530121 12.501847

H 9.832879 15.632439 12.692564

H 9.825725 16.684273 11.599849

O 17.116367 18.663292 14.868435

H 16.263685 18.214640 15.125405

H 16.875850 19.602068 14.807158

O 11.776994 14.737871 19.762001

H 12.526024 14.207982 19.347956

H 11.556539 14.285023 20.591343

O 12.381509 11.821389 15.360146

H 12.147859 11.290512 14.547896

H 11.627133 12.426175 15.548784

O 18.645547 18.190728 17.136227

H 17.993995 18.361894 17.858586

H 18.166768 18.481634 16.326907

O 14.692113 14.976421 10.835633

H 15.481798 15.323564 10.355286

H 15.022426 14.954297 11.768858

O 18.329225 13.572226 17.753978

H 17.660438 13.808436 18.439388

H 17.814100 12.956708 17.189848

O 13.183466 17.154000 11.302380

H 12.963669 16.783908 12.202470

H 13.805935 16.448276 10.975479

O 9.525646 12.005476 13.423119

H 10.255481 11.350785 13.278528

H 9.359712 11.967800 14.377659

O 15.656414 11.670567 10.987263

H 16.326570 12.402959 10.825273

H 16.031216 10.897104 10.537271

O 10.890155 16.455175 10.124453

H 10.716007 16.907587 9.285144

H 11.729287 16.861695 10.476340

O 18.083067 14.051925 13.581391

H 18.296567 13.710609 14.466338

H 18.659381 14.888872 13.520571

O 14.031451 11.332453 17.329531

H 13.852252 10.501867 17.795717

H 13.362909 11.389839 16.586648

O 18.377180 15.759299 16.225670

H 18.493096 16.621121 16.736361

H 18.544389 14.991363 16.843868

O 11.195128 17.349700 16.815677

H 10.561073 16.772151 17.305833

H 11.983362 16.764129 16.733700

O 15.923798 16.408362 20.602125

H 16.293107 17.002768 19.896103

H 14.945221 16.525004 20.487201

O 13.306322 16.905898 20.040312

H 12.895150 17.541408 20.647091

H 12.674821 16.134481 19.996119

O 12.404826 19.824697 16.985297

H 12.707848 19.878821 16.053904

H 11.808129 19.041089 16.974890

O 10.935342 17.901696 14.190182

H 10.754101 17.661007 15.133059

H 10.274898 17.439182 13.582284

O 14.566027 19.263645 12.035751

H 14.007659 18.522612 11.649181

H 14.571883 19.965629 11.366306

O 12.893638 11.459896 10.908585

H 13.863366 11.629815 10.924497

H 12.495485 12.312933 10.632372

O 17.640764 13.411335 10.820849

H 18.006197 13.482504 11.722985

H 17.490263 14.350297 10.565204

O 14.295681 18.244934 17.911682

H 13.624340 18.947555 17.648681

H 13.839560 17.695523 18.600182

O 9.859341 15.276494 17.977453

H 10.499642 15.027773 18.701478

H 8.978413 15.247043 18.381115

O 16.170112 12.003481 13.560588

H 16.848566 12.683431 13.382090

H 15.947005 11.661893 12.651390

O 16.393263 18.981387 21.658178

H 15.508178 19.352686 21.795636

H 16.225742 18.015318 21.633350

O 10.694376 13.902473 15.858806

H 10.216441 14.316111 16.629771

H 10.392494 14.380968 15.068499

O 11.789210 10.597096 13.086372

H 11.809156 9.631199 13.010636

H 12.263589 10.950649 12.257803

O 19.388922 16.252051 13.823992

H 20.350228 16.128159 13.810712

H 19.135668 16.135167 14.782319

O 13.812336 13.717926 18.492283

H 13.839109 12.784196 18.145217

H 14.723033 13.870586 18.873929

O 10.876372 14.227937 12.924171

H 11.337606 14.078915 12.053258

H 10.378488 13.381100 13.126988

O 16.715398 18.460574 19.013436

H 16.656116 18.919141 19.888053

H 15.817189 18.554660 18.597955

O 16.771331 17.746922 12.348639

H 17.163662 18.076580 13.196573

H 16.008596 18.366926 12.188513

O 13.206203 19.319944 14.365910

H 12.346378 18.894150 14.124897

H 13.674790 19.489406 13.509470

O 16.377678 12.198770 16.294400

H 15.585654 11.780293 16.715845

H 16.324377 11.929524 15.351439

O 16.265029 14.027855 19.554088

H 16.338107 13.388024 20.280055

H 16.202837 14.924519 20.010828

O 12.114901 14.065341 10.538339

H 13.047545 14.381311 10.596532

H 11.614125 14.828427 10.158852

O 17.101628 16.054607 10.244197

H 17.405875 16.498547 9.438511

H 17.152707 16.733182 10.961714

XYZ Coordinates from $Co(H_2O)_6^{2+}$

Co 14.380444 15.428726 14.898697

O 15.838971 14.873199 16.209000

H 16.743987 15.293761 16.142306

H 15.996802 13.892347 16.270032

O 12.974084 15.221718 16.475608

H 12.171062 14.711330 16.185352

H 13.360531 14.680316 17.226877

O 14.772325 17.456289 15.384578

H 14.616324 17.616253 16.367982

H 14.151575 18.125137 14.954081

O 12.735822 16.024307 13.723433

H 12.144231 15.241981 13.538605

H 12.092012 16.745115 14.023597

O 14.016637 13.517723 14.219704

H 14.805740 12.965830 13.969973

H 13.404074 12.899625 14.701114

O 15.771791 15.514302 13.318954

H 16.112641 16.403384 12.988145

H 16.581510 14.973807 13.509680

O 9.490878 16.513184 12.534874

H 9.877247 15.620844 12.717757

H 9.847823 16.679466 11.633064

O 17.124628 18.687198 14.857901

H 16.273702 18.222970 15.097296

H 16.870053 19.622198 14.796892

O 11.779294 14.707866 19.757577

H 12.527409 14.178573 19.340503

H 11.573233 14.264661 20.595707

O 12.339616 11.804518 15.397677

H 12.118428 11.290688 14.571428

H 11.591813 12.420628 15.576861

O 18.641993 18.193311 17.120918

H 17.987582 18.365078 17.840838

H 18.171111 18.496823 16.311230

O 14.709228 14.999234 10.862648

H 15.506177 15.339194 10.387869

H 15.029126 14.979552 11.799577

O 18.329101 13.588897 17.726700

H 17.669644 13.810865 18.425952

H 17.810573 12.969876 17.169353

O 13.201022 17.174355 11.327266

H 12.991626 16.808052 12.230320

H 13.823303 16.469102 10.998143

O 9.537654 12.040403 13.407259

H 10.256248 11.370349 13.276143

H 9.351574 12.004677 14.358208

O 15.641798 11.679381 10.996694

H 16.320561 12.403507 10.828280

H 16.017848 10.892793 10.571163

O 10.909128 16.466682 10.153500

H 10.738320 16.915649 9.311643

H 11.747866 16.873523 10.506592

O 18.079091 14.045431 13.553103

H 18.342441 13.667293 14.409182

H 18.639185 14.894168 13.500760

O 14.034536 11.298804 17.328312

H 13.875314 10.466010 17.797695

H 13.347730 11.352756 16.603514

O 18.332072 15.770509 16.198542

H 18.459848 16.631591 16.708510

H 18.512559 15.002986 16.814994

O 11.212127 17.341148 16.858691

H 10.566241 16.769619 17.339696

H 11.993522 16.743932 16.778082

O 15.909103 16.397849 20.572704

H 16.287779 16.995720 19.874972

H 14.932136 16.513638 20.444056

O 13.297453 16.888203 19.974115

H 12.886647 17.532590 20.571756

H 12.672222 16.110608 19.952231

O 12.423123 19.822734 16.965977

H 12.723192 19.870623 16.032889

H 11.820210 19.044520 16.965446

O 10.970671 17.864604 14.230575

H 10.787282 17.639897 15.177683

H 10.303675 17.410476 13.623654

O 14.581729 19.297198 12.020998

H 14.023137 18.551192 11.645748

H 14.584853 19.990955 11.343130

O 12.881551 11.463950 10.921190

H 13.852093 11.629928 10.933398

H 12.486686 12.321358 10.653426

O 17.639571 13.396153 10.814194

H 18.003514 13.462694 11.718311

H 17.496735 14.337507 10.563344

O 14.308095 18.212624 17.851288

H 13.641253 18.924906 17.603180

H 13.849933 17.658490 18.534757

O 9.853229 15.268121 17.992527

H 10.493603 15.010806 18.713262

H 8.972136 15.234154 18.395400

O 16.143760 12.034656 13.570094

H 16.803938 12.730351 13.381376

H 15.912400 11.690767 12.663719

O 16.315040 18.989411 21.623452

H 15.418604 19.342370 21.732978

H 16.168838 18.019999 21.603037

O 10.676651 13.910207 15.859596

H 10.205843 14.321234 16.636463

H 10.375690 14.399736 15.076122

O 11.781410 10.602065 13.100685

H 11.802643 9.636314 13.024078

H 12.254782 10.957779 12.272399

O 19.355485 16.261771 13.802710

H 20.317400 16.142057 13.794646

H 19.098038 16.148450 14.760563

O 13.810096 13.688203 18.482826

H 13.835539 12.753813 18.138862

H 14.721279 13.843714 18.861410

O 10.942501 14.233753 12.932532

H 11.386515 14.088876 12.052125

H 10.429951 13.395057 13.132623

O 16.708254 18.454251 18.990055

H 16.622982 18.918957 19.859131

H 15.816681 18.530344 18.556104

O 16.767360 17.751042 12.350235

H 17.170873 18.090956 13.188447

H 16.014868 18.380219 12.180550

O 13.213004 19.318269 14.348213

H 12.353871 18.886666 14.114465

H 13.668475 19.509357 13.490003

O 16.363593 12.217891 16.295616

H 15.576320 11.782297 16.708809

H 16.325525 11.948675 15.351727

O 16.267676 14.014970 19.533326

H 16.343353 13.382952 20.265872

H 16.198979 14.915089 19.981864

O 12.144177 14.076972 10.526885

H 13.075861 14.395067 10.590149

H 11.636205 14.845525 10.167751

O 17.126172 16.050626 10.266190

H 17.411632 16.505235 9.459535

H 17.165230 16.728993 10.985869

XYZ Coordinates from $Cu(H_2O)_6^{2+}$

Cu 14.436910 15.512399 14.894349

O 15.717688 14.967782 16.301364

H 16.653833 15.337353 16.266671

H 15.845378 13.976197 16.352329

O 12.863718 15.272068 16.592967

H 12.120387 14.713990 16.273017

H 13.276888 14.737525 17.324586

O 14.744986 17.422661 15.425621

H 14.571765 17.564825 16.415238

H 14.123768 18.093233 14.982888

O 12.885659 16.008254 13.715418

H 12.347693 15.192875 13.484630

H 12.187502 16.657286 14.076535

O 14.270435 13.682946 14.147698

H 15.152586 13.230703 14.022036

H 13.685571 13.045699 14.650679

O 15.908389 15.725656 13.137649

H 16.021863 16.651178 12.796875

H 16.846581 15.493305 13.366677

O 9.563815 16.395252 12.567274

H 10.038384 15.535142 12.684579

H 9.866693 16.638550 11.663837

O 17.113382 18.613030 14.789560

H 16.282486 18.135018 15.057798

H 16.871499 19.550273 14.872407

O 11.682401 14.722952 19.794894

H 12.441940 14.182062 19.409449

H 11.463341 14.308895 20.644592

O 12.568279 12.044872 15.334669

H 12.311069 11.459323 14.562105

H 11.790790 12.623335 15.511922

O 18.667665 18.078523 17.044718

H 18.003903 18.255419 17.756523

H 18.201304 18.366221 16.227486

O 14.749232 15.124122 10.781104

H 15.522978 15.467067 10.273305

H 15.126021 15.108353 11.700824

O 18.222781 13.538528 17.976206

H 17.566433 13.772622 18.675079

H 17.675311 12.967671 17.391415

O 13.204673 17.246440 11.325300

H 13.045935 16.858428 12.227059

H 13.843505 16.571812 10.962650

O 9.635937 12.178771 13.537300

H 10.307760 11.458900 13.452745

H 9.632762 12.394971 14.486029

O 15.600960 11.779962 11.238422

H 16.222665 12.507278 10.909590

H 15.939926 10.975915 10.813807

O 10.923927 16.482475 10.161302

H 10.752278 16.950505 9.329993

H 11.748081 16.902182 10.534009

O 18.831461 13.025461 12.872810

H 19.659992 12.574405 13.092789

H 18.898058 13.950644 13.244036

O 13.980912 11.313152 17.404713

H 13.773039 10.448148 17.788212

H 13.361918 11.446188 16.629477

O 18.241296 15.609224 16.359977

H 18.464481 16.516456 16.748583

H 18.389241 14.879387 17.037929

O 11.096361 17.309103 16.956969

H 10.427643 16.732558 17.396116

H 11.878663 16.701055 16.908602

O 15.836432 16.460638 20.631987

H 16.222122 16.988444 19.883638

H 14.860512 16.577258 20.499853

O 13.218880 16.893533 19.995594

H 12.780816 17.565858 20.540837

H 12.585597 16.122109 19.967990

O 12.356014 19.769348 17.029724

H 12.633013 19.813704 16.089190

H 11.734642 19.005829 17.046005

O 11.029276 17.684221 14.314119

H 10.801251 17.503342 15.264095

H 10.355911 17.262818 13.689891

O 14.375468 19.499417 12.017811

H 13.890329 18.706603 11.640241

H 14.230891 20.223164 11.388749

O 12.878169 11.502869 10.996929

H 13.844375 11.689520 11.077268

H 12.496308 12.345902 10.673391

O 17.496603 13.437817 10.532874

H 18.107005 13.263262 11.285721

H 17.423147 14.415881 10.499243

O 14.259931 18.163341 17.859974

H 13.583296 18.873736 17.625666

H 13.815896 17.609891 18.554587

O 9.776084 15.151202 17.976542

H 10.418845 14.930305 18.708332

H 8.892757 15.058171 18.365311

O 16.429491 12.190346 13.748978

H 17.343849 12.501973 13.515737

H 16.080741 11.848349 12.883373

O 16.425118 19.082194 21.493164

H 15.556246 19.500199 21.596108

H 16.215900 18.125361 21.543429

O 10.544886 13.886649 15.769537

H 10.137273 14.266363 16.600107

H 10.368905 14.550471 15.081863

O 11.841070 10.666228 13.213581

H 11.924853 9.701074 13.197843

H 12.262244 11.002074 12.348164

O 18.520473 15.437502 13.803262

H 19.022844 16.205820 13.490756

H 18.513128 15.494922 14.813303

O 13.736030 13.684619 18.602923

H 13.763628 12.754534 18.252865

H 14.633235 13.829867 19.014835

O 11.198638 14.212314 12.832409

H 11.583450 14.072754 11.924653

H 10.648625 13.403962 13.050818

O 16.714178 18.356711 18.893981

H 16.682084 18.876291 19.735922

H 15.814665 18.460078 18.484123

O 16.703855 18.114278 12.131734

H 17.045841 18.334529 13.033260

H 15.919590 18.710170 12.021394

O 13.177649 19.254583 14.417266

H 12.323148 18.803547 14.199097

H 13.579262 19.524736 13.551198

O 16.266471 12.351901 16.426562

H 15.506857 11.842021 16.804811

H 16.322998 12.086753 15.477329

O 16.153433 14.017673 19.737654

H 16.206753 13.410187 20.492479

H 16.088344 14.935438 20.149813

O 12.239402 14.104526 10.347760

H 13.158346 14.454491 10.434468

H 11.693347 14.885539 10.083038

O 17.152194 16.197140 10.236136

H 17.515045 16.598405 9.432545

H 17.109495 16.928591 10.900704

XYZ Coordinates from $Ni(H_2O)_6^{2+}$

Ni 14.332032 15.441701 14.911227

O 15.762615 14.868178 16.223891

H 16.652348 15.314927 16.143443

H 15.957266 13.893113 16.207957

O 12.975061 15.285056 16.465389

H 12.174681 14.762351 16.184821

H 13.376727 14.748566 17.213914

O 14.759497 17.400932 15.424478

H 14.598569 17.557553 16.406996

H 14.154515 18.080935 14.986860

O 12.774926 16.022513 13.687714

H 12.200699 15.228249 13.502387

H 12.116878 16.724571 14.003118

O 13.923077 13.561791 14.241307

H 14.706589 13.027178 13.938736

H 13.369538 12.934263 14.774845

O 15.700640 15.472510 13.349568

H 16.049688 16.363118 13.030015

H 16.510525 14.945127 13.566253

O 9.505906 16.509065 12.521791

H 9.892922 15.616303 12.702534

H 9.865079 16.681789 11.622395

O 17.098317 18.677909 14.882543

H 16.261784 18.198327 15.134734

H 16.818493 19.603758 14.796947

O 11.782751 14.727136 19.760907

H 12.535957 14.212121 19.334815

H 11.569995 14.260826 20.584653

O 12.329392 11.784533 15.469019

H 12.138989 11.292689 14.622543

H 11.587481 12.413178 15.627856

O 18.633121 18.212431 17.136565

H 17.983293 18.385601 17.859898

H 18.159723 18.522221 16.331121

O 14.718077 14.972103 10.862334

H 15.532325 15.300213 10.408384

H 15.008137 14.949819 11.809092

O 18.305541 13.592944 17.653956

H 17.656074 13.802389 18.366256

H 17.782157 12.985233 17.088311

O 13.232648 17.171887 11.299177

H 13.024130 16.804834 12.202800

H 13.851174 16.463050 10.971737

O 9.580493 12.018449 13.389584

H 10.309685 11.357062 13.276423

H 9.370458 11.979391 14.335267

O 15.671056 11.663780 10.906463

H 16.370379 12.373022 10.761782

H 16.061861 10.856795 10.536495

O 10.930550 16.470238 10.140636

H 10.760282 16.912654 9.295188

H 11.773327 16.874658 10.486151

O 18.035068 14.022584 13.537150

H 18.340952 13.607982 14.361300

H 18.578916 14.883478 13.491002

O 14.074898 11.337655 17.355489

H 13.934166 10.518197 17.853383

H 13.365794 11.367536 16.650480

O 18.248414 15.809300 16.177985

H 18.405078 16.656523 16.700607

H 18.449393 15.027451 16.769600

O 11.189471 17.370829 16.857467

H 10.548140 16.794077 17.337927

H 11.980396 16.784742 16.785896

O 15.948491 16.398731 20.605498

H 16.314261 17.003757 19.906345

H 14.969820 16.510815 20.490694

O 13.322806 16.884689 20.044204

H 12.914523 17.530957 20.641418

H 12.685483 16.117737 20.006004

O 12.452626 19.817874 16.985331

H 12.758547 19.854674 16.053456

H 11.826354 19.058065 16.979674

O 10.995193 17.839250 14.221938

H 10.795335 17.627295 15.169019

H 10.322977 17.396387 13.611731

O 14.595402 19.290724 12.037422

H 14.041025 18.553729 11.639117

H 14.616176 19.996693 11.372650

O 12.908358 11.463141 10.927059

H 13.878340 11.627554 10.900804

H 12.502505 12.315099 10.659443

O 17.692934 13.358101 10.798740

H 18.031737 13.417307 11.713890

H 17.541769 14.301577 10.561667

O 14.301953 18.177397 17.884886

H 13.641276 18.894028 17.629949

H 13.849914 17.641230 18.586489

O 9.854637 15.280864 17.989878

H 10.497424 15.028766 18.710208

H 8.973247 15.230604 18.390334

O 16.031760 12.126500 13.486432

H 16.716565 12.804546 13.316370

H 15.846057 11.750132 12.582735

O 16.318196 19.002376 21.644005

H 15.415307 19.336373 21.759606

H 16.193818 18.029885 21.631850

O 10.709279 13.935523 15.862327

H 10.223441 14.346694 16.630151

H 10.402923 14.401923 15.066734

O 11.854857 10.612827 13.135235

H 11.881557 9.647323 13.056001

H 12.316725 10.969475 12.301404

O 19.279661 16.253462 13.785295

H 20.241914 16.136865 13.792108

H 19.008091 16.156821 14.742695

O 13.819795 13.749606 18.465660

H 13.854389 12.812888 18.128789

H 14.733740 13.906754 18.836824

O 10.957925 14.232554 12.912151

H 11.399890 14.084896 12.031727

H 10.460194 13.386533 13.115838

O 16.701152 18.458897 19.010686

H 16.611704 18.928833 19.876402

H 15.807552 18.516606 18.576978

O 16.736731 17.694966 12.389784

H 17.150936 18.038813 13.220624

H 16.003536 18.343509 12.208844

O 13.240691 19.281353 14.369598

H 12.377569 18.858930 14.132594

H 13.696185 19.479892 13.512649

O 16.358977 12.219116 16.199978

H 15.585833 11.791874 16.647307

H 16.276785 11.963050 15.254915

O 16.285859 14.036062 19.508663

H 16.347393 13.385676 20.226362

H 16.229526 14.925458 19.978818

O 12.154361 14.068110 10.499240

H 13.087405 14.380971 10.569519

H 11.648815 14.844574 10.154496

O 17.146976 16.016748 10.301451

H 17.417787 16.488827 9.499812

H 17.166848 16.687271 11.029867

XYZ Coordinates from $Fe(H_2O)_6^{3+}$

Fe 14.352454 15.413669 14.952655

O 15.791657 14.880976 16.221129

H 16.714259 15.345425 16.206822

H 15.968419 13.883276 16.245388

O 13.023681 15.182855 16.497354

H 12.160630 14.728999 16.233525

H 13.400402 14.640157 17.281884

O 14.691275 17.362520 15.439574

H 14.566265 17.576616 16.444499

H 14.105123 18.075473 14.975997

O 12.786083 15.952208 13.795296

H 12.203685 15.164926 13.490390

H 12.100279 16.687749 14.067836

O 14.049355 13.534954 14.375134

H 14.788052 12.984284 13.939940

H 13.379432 12.891383 14.789330

O 15.703685 15.474518 13.399912

H 16.045075 16.377103 13.034543

H 16.530644 14.930784 13.563826

O 9.554060 16.473301 12.532420

H 9.974631 15.592179 12.681491

H 9.867546 16.664890 11.619122

O 17.096932 18.673017 14.872902

H 16.273391 18.197462 15.139985

H 16.814009 19.598672 14.791049

O 11.803581 14.748164 19.834755

H 12.529342 14.206510 19.404680

H 11.610002 14.315306 20.681391

O 12.336535 11.881601 15.358981

H 12.128847 11.331479 14.545696

H 11.566936 12.467338 15.544578

O 18.618800 18.164479 17.135265

H 17.983896 18.375415 17.863407

H 18.165513 18.505643 16.331836

O 14.711622 14.997243 10.805256

H 15.537196 15.349363 10.383416

H 14.985337 14.887830 11.742331

O 18.318968 13.593187 17.736480

H 17.644939 13.784555 18.432502

H 17.828096 12.973948 17.158313

O 13.190327 17.142067 11.318448

H 12.993311 16.801611 12.224059

H 13.814671 16.433624 10.994015

O 9.668574 12.148014 13.343309

H 10.322103 11.411624 13.241053

H 9.364443 12.076915 14.260847

O 15.668111 11.669518 10.889356

H 16.362376 12.380835 10.731564

H 16.060112 10.859537 10.526676

O 10.898988 16.441969 10.116023

H 10.716643 16.918131 9.291503

H 11.732690 16.848153 10.476241

O 17.980412 14.069730 13.532239

H 18.268445 13.647190 14.359752

H 18.547888 14.920142 13.500472

O 14.035326 11.354736 17.316397

H 13.904145 10.513736 17.780762

H 13.341437 11.395521 16.602629

O 18.168251 15.776527 16.260372

H 18.340146 16.651060 16.750555

H 18.421916 15.009640 16.858301

O 11.165346 17.371629 16.883920

H 10.544413 16.787921 17.385566

H 11.978305 16.826409 16.840834

O 15.961184 16.396594 20.563133

H 16.340096 17.004067 19.874366

H 14.985216 16.522099 20.439531

O 13.349910 16.915118 19.969889

H 12.958564 17.580373 20.558051

H 12.714308 16.145806 19.980163

O 12.459253 19.829309 16.993470

H 12.740599 19.882760 16.056365

H 11.809717 19.090945 16.989151

O 11.060074 17.732380 14.232223

H 10.811722 17.584779 15.181509

H 10.363725 17.334069 13.607254

O 14.587034 19.246558 12.032522

H 14.013145 18.530052 11.627296

H 14.625161 19.964421 11.381091

O 12.904528 11.447223 10.922296

H 13.873734 11.612328 10.889268

H 12.498858 12.283000 10.610765

O 17.673201 13.383326 10.758266

H 18.019787 13.457566 11.667917

H 17.528454 14.322163 10.502072

O 14.318779 18.182125 17.818297

H 13.649805 18.905232 17.588017

H 13.878414 17.634657 18.524032

O 9.870616 15.290808 18.075675

H 10.508364 15.040436 18.801585

H 8.984944 15.236193 18.466473

O 15.993302 12.141247 13.450413

H 16.705367 12.790808 13.280640

H 15.812056 11.760636 12.542062

O 16.310140 18.994605 21.632843

H 15.405527 19.320758 21.757494

H 16.194039 18.021451 21.621648

O 10.720139 14.030684 15.915711

H 10.242604 14.376784 16.724686

H 10.330152 14.515871 15.169477

O 11.838767 10.598112 13.122989

H 11.876640 9.631761 13.062364

H 12.301061 10.952359 12.288230

O 19.229652 16.275345 13.845457

H 20.195049 16.185166 13.850367

H 18.963124 16.164913 14.797553

O 13.808073 13.733603 18.477847

H 13.826241 12.796133 18.137275

H 14.725613 13.880890 18.856942

O 11.153844 14.253628 12.836890

H 11.530720 14.089114 11.925854

H 10.638936 13.428497 13.093100

O 16.715231 18.488084 19.004534

H 16.610810 18.939303 19.879749

H 15.832020 18.548975 18.558942

O 16.682409 17.625390 12.414346

H 17.129518 18.003003 13.213770

H 15.955740 18.282924 12.220685

O 13.281904 19.225504 14.375570

H 12.407254 18.828306 14.138410

H 13.734704 19.427373 13.514373

O 16.310536 12.302940 16.228672

H 15.542685 11.847747 16.664162

H 16.282529 11.997778 15.296980

O 16.249032 14.014329 19.520600

H 16.310730 13.379564 20.252424

H 16.210341 14.918065 19.970003

O 12.165030 14.048068 10.366733

H 13.093452 14.372643 10.427464

H 11.641814 14.827040 10.054145

O 17.122767 16.041649 10.238532

H 17.354686 16.532385 9.435607

H 17.157494 16.696412 10.977927

XYZ Coordinates from $Co(H_2O)_6^{3+}$

Co 14.335743 15.386929 14.926668

O 15.671025 14.844261 16.139130

H 16.571780 15.341815 16.089702

H 15.869717 13.849654 16.132207

O 13.084023 15.272689 16.356500

H 12.220350 14.807302 16.096447

H 13.460605 14.722119 17.142989

O 14.744770 17.191567 15.410794

H 14.574999 17.376674 16.410431

H 14.165975 17.907499 14.945685

O 12.932652 15.947583 13.759155

H 12.361184 15.148008 13.476494

H 12.224476 16.631349 14.100220

O 13.968769 13.625799 14.353126

H 14.738144 13.082160 13.966452

H 13.387963 12.992622 14.891606

O 15.576200 15.452165 13.470949

H 15.898887 16.379114 13.147249

H 16.421361 14.942068 13.650818

O 9.600338 16.452895 12.558119

H 10.041813 15.577275 12.685117

H 9.889898 16.663075 11.641332

O 17.104261 18.671510 14.930082

H 16.324336 18.135324 15.203730

H 16.750684 19.573721 14.859786

O 11.823623 14.741644 19.821711

H 12.554097 14.250206 19.340863

H 11.657779 14.247871 20.640369

O 12.372621 11.922034 15.449218

H 12.203756 11.378616 14.622635

H 11.589378 12.499894 15.597077

O 18.615193 18.129143 17.175998

H 17.984568 18.338135 17.908265

H 18.182086 18.522648 16.386485

O 14.702475 14.982585 10.806718

H 15.558243 15.321392 10.436462

H 14.914211 14.861983 11.757167

O 18.272626 13.624288 17.595596

H 17.621717 13.832529 18.308745

H 17.767402 12.983514 17.053508

O 13.211981 17.168680 11.272355

H 13.045504 16.837163 12.186701

H 13.828196 16.456792 10.943361

O 9.759047 12.151099 13.371253

H 10.420582 11.418319 13.304203

H 9.419813 12.093411 14.277222

O 15.657169 11.678556 10.888707

H 16.370473 12.373144 10.737792

H 16.053861 10.847428 10.583346

O 10.897804 16.445714 10.115973

H 10.703278 16.918680 9.292345

H 11.735185 16.855216 10.462807

O 17.887840 14.096583 13.555548

H 18.214244 13.697845 14.380798

H 18.443985 14.949930 13.472926

O 14.029119 11.366057 17.405208

H 13.875206 10.565293 17.929333

H 13.325542 11.392616 16.699691

O 18.029417 15.816353 16.154230

H 18.234352 16.655064 16.688517

H 18.308615 15.025868 16.711718

O 11.187313 17.397139 16.921957

H 10.577132 16.793170 17.413699

H 12.009024 16.868335 16.859854

O 15.977659 16.382515 20.625802

H 16.346262 16.996725 19.937298

H 15.000951 16.503212 20.507153

O 13.369260 16.899725 20.023264

H 12.968572 17.586561 20.579306

H 12.721867 16.140626 20.025711

O 12.572536 19.797699 16.991733

H 12.844561 19.810007 16.049504

H 11.871146 19.110144 17.011189

O 11.142871 17.645290 14.266706

H 10.871940 17.530579 15.214988

H 10.438739 17.269901 13.634819

O 14.588385 19.279599 12.020884

H 14.022024 18.577020 11.585059

H 14.646972 20.017183 11.393786

O 12.904016 11.453638 10.937219

H 13.871800 11.623795 10.889304

H 12.491469 12.284091 10.620312

O 17.689439 13.356376 10.789504

H 18.009021 13.407956 11.710204

H 17.549147 14.302469 10.557481

O 14.335136 18.023601 17.789789

H 13.706669 18.782815 17.564131

H 13.890878 17.531641 18.533095

O 9.903235 15.291334 18.059788

H 10.528147 15.031262 18.793772

H 9.008730 15.222714 18.427639

O 15.906737 12.187918 13.454646

H 16.643656 12.813458 13.295680

H 15.724224 11.816604 12.542292

O 16.199129 19.044205 21.618302

H 15.279189 19.333121 21.720971

H 16.129956 18.068093 21.653170

O 10.784401 14.114721 15.867744

H 10.308788 14.448542 16.684485

H 10.357135 14.566973 15.121185

O 11.953265 10.626335 13.199541

H 11.988536 9.659374 13.147537

H 12.369666 10.972022 12.337760

O 19.104014 16.336078 13.749175

H 20.070727 16.260967 13.757258

H 18.836171 16.248631 14.703115

O 13.808921 13.833432 18.361157

H 13.822040 12.874830 18.082207

H 14.726447 13.984086 18.740420

O 11.229745 14.269412 12.841612

H 11.578437 14.103052 11.920104

H 10.727840 13.441012 13.108030

O 16.692788 18.456700 19.022610

H 16.542222 18.939388 19.873699

H 15.821584 18.456088 18.548684

O 16.587210 17.585666 12.505259

H 17.085453 17.970623 13.268696

H 15.908557 18.282098 12.275143

O 13.366312 19.108339 14.397772

H 12.475435 18.742732 14.166977

H 13.789907 19.368564 13.537745

O 16.248239 12.280720 16.185835

H 15.508933 11.825747 16.667334

H 16.195316 11.949157 15.263165

O 16.246508 14.070579 19.421640

H 16.292905 13.380917 20.103554

H 16.217262 14.936935 19.939569

O 12.162206 14.047063 10.340785

H 13.090701 14.373733 10.386766

H 11.632808 14.827324 10.041799

O 17.140299 16.021809 10.351835

H 17.365873 16.540725 9.564841

H 17.124553 16.663569 11.105246

XYZ Coordinates from $Cu(H_2O)_6^{3+}$

Cu 14.339531 15.401009 14.927153

O 15.725690 14.861017 16.212363

H 16.634632 15.341193 16.106570

H 15.914950 13.863545 16.172522

O 13.030075 15.274138 16.422816

H 12.185983 14.785536 16.142541

H 13.427869 14.708841 17.188628

O 14.762799 17.293733 15.422044

H 14.578360 17.457086 16.422211

H 14.149006 17.983102 14.961432

O 12.852016 15.977651 13.730576

H 12.282891 15.167188 13.483220

H 12.159125 16.665226 14.081713

O 13.954380 13.578229 14.276671

H 14.765605 13.047335 13.964178

H 13.385559 12.950479 14.835789

O 15.641667 15.450907 13.407367

H 15.974115 16.381264 13.103129

H 16.477332 14.937696 13.616496

O 9.567993 16.472465 12.544912

H 9.992298 15.590826 12.686551

H 9.879909 16.672118 11.632910

O 17.115873 18.690543 14.902589

H 16.314351 18.179232 15.170926

H 16.794765 19.604066 14.825568

O 11.811807 14.726764 19.819922

H 12.542912 14.223586 19.352866

H 11.634374 14.244991 20.643275

O 12.373282 11.889817 15.421157

H 12.178749 11.351786 14.596159

H 11.600312 12.475458 15.592373

O 18.609053 18.124112 17.152148

H 17.971263 18.331487 17.879097

H 18.178656 18.511411 16.357404

O 14.715276 14.992854 10.792529

H 15.563432 15.327176 10.403384

H 14.949661 14.881147 11.739810

O 18.292722 13.622525 17.643729

H 17.633287 13.823280 18.351127

H 17.794683 12.982792 17.093645

O 13.215593 17.169241 11.273612

H 13.031196 16.823686 12.181688

H 13.836020 16.462242 10.942883

O 9.699479 12.139756 13.362109

H 10.366970 11.413623 13.277117

H 9.394677 12.081186 14.280320

O 15.655742 11.656240 10.938216

H 16.360750 12.354297 10.768591

H 16.038473 10.831681 10.599260

O 10.902015 16.454878 10.121709

H 10.707026 16.924638 9.296429

H 11.742640 16.862421 10.464373

O 17.947474 14.079999 13.556660

H 18.260956 13.698126 14.394612

H 18.497802 14.935333 13.470922

O 14.049385 11.361353 17.375845

H 13.904666 10.551639 17.888938

H 13.351486 11.381051 16.664994

O 18.089282 15.791917 16.162356

H 18.274959 16.645202 16.685122

H 18.352345 15.011657 16.742021

O 11.157613 17.396953 16.911006

H 10.553682 16.788084 17.403673

H 11.982164 16.871710 16.843857

O 15.970132 16.383490 20.600934

H 16.338043 16.992919 19.907602

H 14.992827 16.502588 20.484463

O 13.354853 16.889337 20.014350

H 12.956068 17.566717 20.583193

H 12.711140 16.127288 20.014579

O 12.516458 19.813719 17.000344

H 12.791364 19.837339 16.059430

H 11.827079 19.113786 17.012669

O 11.081365 17.697000 14.260149

H 10.821631 17.559773 15.208287

H 10.388095 17.304349 13.627312

O 14.589093 19.270308 12.043721

H 14.025189 18.565317 11.606679

H 14.656288 20.000423 11.408676

O 12.897691 11.457266 10.942065

H 13.867545 11.621113 10.914545

H 12.494772 12.293895 10.629088

O 17.681542 13.340764 10.787879

H 18.039549 13.387657 11.694078

H 17.540097 14.288318 10.562575

O 14.311517 18.079685 17.810389

H 13.665735 18.822488 17.579489

H 13.869249 17.564518 18.538457

O 9.890184 15.278250 18.059314

H 10.520706 15.022243 18.790270

H 8.999249 15.214269 18.436468

O 15.969826 12.180101 13.489756

H 16.690086 12.820064 13.310921

H 15.780408 11.787385 12.587924

O 16.268005 19.020713 21.616875

H 15.356903 19.329603 21.738070

H 16.174993 18.045772 21.635985

O 10.764065 14.067272 15.885256

H 10.282841 14.408147 16.695579

H 10.352740 14.526026 15.133651

O 11.892977 10.618281 13.173371

H 11.932077 9.651674 13.117167

H 12.330289 10.969063 12.323075

O 19.152774 16.327880 13.750046

H 20.120308 16.263729 13.749453

H 18.896161 16.231050 14.705019

O 13.805963 13.803316 18.382772

H 13.825920 12.851511 18.081401

H 14.721903 13.954327 18.765180

O 11.163019 14.265252 12.846568

H 11.536398 14.103034 11.934587

H 10.656664 13.435098 13.100394

O 16.691936 18.455161 19.003497

H 16.570854 18.929191 19.864104

H 15.810425 18.480524 18.549126

O 16.636120 17.609262 12.478045

H 17.110693 17.992294 13.258795

H 15.930368 18.284085 12.266530

O 13.314451 19.152976 14.397467

H 12.431198 18.770377 14.165309

H 13.749367 19.389798 13.536321

O 16.284382 12.294938 16.204300

H 15.537429 11.835744 16.670188

H 16.241510 11.972497 15.277132

O 16.241985 14.047772 19.449416

H 16.287110 13.376316 20.149132

H 16.210056 14.927704 19.943929

O 12.163687 14.057670 10.366754

H 13.093663 14.379841 10.411943

H 11.639250 14.836695 10.056664

O 17.162330 16.013590 10.335417

H 17.415820 16.510800 9.543028

H 17.156877 16.668058 11.076967

XYZ Coordinates from $Ni(H_2O)_6^{3+}$

Ni 14.397423 15.355609 14.905167

O 15.725829 14.855919 16.062644

H 17.230792 15.604814 16.264455

H 15.828382 13.856424 16.174522

O 12.998675 15.251324 16.384999

H 12.172902 14.755604 16.098390

H 13.376828 14.716096 17.158137

O 14.768806 17.326019 15.396978

H 14.608465 17.478681 16.384020

H 14.149581 18.006364 14.961783

O 12.851988 15.936707 13.728822

H 12.271937 15.150059 13.473675

H 12.176922 16.640956 14.040547

O 14.002713 13.495015 14.226972

H 14.792912 12.935646 13.975290

H 13.394018 12.895909 14.747177

O 15.632079 15.464053 13.286744

H 15.983048 16.376689 13.010503

H 16.456209 14.935223 13.489440

O 9.537759 16.438092 12.535472

H 9.970837 15.559602 12.674405

H 9.858344 16.650382 11.629503

O 17.059360 18.697332 14.953437

H 16.219777 18.186329 15.132336

H 16.771901 19.622633 14.879132

O 11.838521 14.740420 19.800637

H 12.574598 14.241279 19.333908

H 11.654658 14.247030 20.615722

O 12.312160 11.835406 15.401046

H 12.123765 11.301816 14.577421

H 11.558644 12.454269 15.539149

O 18.420599 18.152033 17.068019

H 17.752301 18.332201 17.857243

H 17.940670 18.479274 16.225353

O 14.692115 15.022239 10.731818

H 15.517214 15.372754 10.312674

H 14.959354 14.943469 11.677872

O 18.257211 13.507654 17.759702

H 17.614858 13.726644 18.477959

H 17.708785 12.933242 17.181627

O 13.211023 17.185603 11.314146

H 13.041001 16.799203 12.211404

H 13.818610 16.489974 10.939108

O 9.650480 12.083563 13.331727

H 10.330643 11.369764 13.238743

H 9.368932 12.029529 14.257575

O 15.642706 11.705567 10.983819

H 16.318928 12.435082 10.830777

H 16.021387 10.928354 10.543456

O 10.902716 16.485828 10.134103

H 10.722414 16.969746 9.313706

H 11.737003 16.887617 10.499238

O 17.935890 14.109039 13.627514

H 18.164804 13.769892 14.510172

H 18.478161 14.965071 13.569713

O 13.941816 11.343724 17.389657

H 13.760074 10.541234 17.901783

H 13.261017 11.382400 16.658837

O 18.206589 15.728429 16.419955

H 18.406009 17.121389 16.931585

H 18.402339 14.889963 16.964563

O 11.186451 17.359648 16.882173

H 10.569118 16.762692 17.371474

H 11.990323 16.800668 16.793419

O 15.998329 16.399160 20.619364

H 16.349118 16.931787 19.865528

H 15.018512 16.528286 20.527860

O 13.381019 16.897758 20.066339

H 12.967424 17.566649 20.634528

H 12.739605 16.133886 20.041385

O 12.500401 19.785510 17.009018

H 12.780000 19.816145 16.068360

H 11.841500 19.055028 17.017355

O 11.062854 17.702870 14.232124

H 10.827052 17.544205 15.182127

H 10.372033 17.292331 13.614866

O 14.609777 19.284455 12.074918

H 14.046064 18.563039 11.663589

H 14.630052 20.008063 11.429051

O 12.894362 11.478221 10.918450

H 13.862812 11.657818 10.918834

H 12.487407 12.313431 10.607042

O 17.634489 13.432141 10.839837

H 17.969645 13.511882 11.752194

H 17.506868 14.369294 10.565644

O 14.321611 18.103640 17.851202

H 13.669119 18.837268 17.609384

H 13.881923 17.589144 18.579407

O 9.901261 15.249575 18.030463

H 10.541819 15.008117 18.756261

H 9.017282 15.194693 18.424940

O 16.090621 11.990017 13.569823

H 16.784028 12.658244 13.407562

H 15.871293 11.670496 12.650082

O 16.593587 19.062445 21.424420

H 15.746605 19.490829 21.624121

H 16.394030 18.108479 21.527056

O 10.712057 14.014500 15.828468

H 10.255038 14.363582 16.645984

H 10.309700 14.493911 15.085540

O 11.856032 10.583150 13.116393

H 11.888387 9.618078 13.035793

H 12.306335 10.949599 12.280678

O 19.115932 16.382162 13.910583

H 20.083068 16.366608 13.839238

H 18.936751 16.234740 14.870890

O 13.835917 13.799089 18.387543

H 13.827219 12.843055 18.099434

H 14.737263 13.930170 18.804420

O 11.118270 14.222699 12.830359

H 11.501873 14.075359 11.922185

H 10.606812 13.392391 13.064409

O 16.719270 18.397593 18.861441

H 16.727227 18.891569 19.728354

H 15.792852 18.450740 18.480110

O 16.711087 17.660109 12.401852

H 17.216558 17.981589 13.180417

H 16.007130 18.350341 12.247459

O 13.258237 19.204546 14.399142

H 12.393111 18.791118 14.152837

H 13.711748 19.433303 13.547690

O 16.230605 12.246859 16.295617

H 15.466848 11.794417 16.737419

H 16.223027 11.904684 15.374953

O 16.232454 14.007216 19.553669

H 16.236295 13.349009 20.267367

H 16.211531 14.896409 20.026770

O 12.145560 14.075011 10.345871

H 13.073385 14.405219 10.391343

H 11.616831 14.857163 10.052436

O 17.122731 16.070414 10.239263

H 17.427456 16.543703 9.450471

H 17.141458 16.729735 10.977058

XYZ Coordinates from $Fe(H_2O)_5OH^+$

Fe 14.682792 15.727054 15.341499

O 15.902825 15.826715 16.801812

H 16.644135 16.444330 16.711240

O 12.961171 15.465132 16.763887

H 12.256203 14.903235 16.353596

H 13.330949 14.885066 17.485209

O 14.657037 17.979674 15.396333

H 14.512243 18.120197 16.376581

H 13.959487 18.551295 14.959955

O 12.970655 15.986378 13.943119

H 12.478969 15.151682 13.733834

H 12.236009 16.629356 14.201433

O 14.504054 13.679474 14.889026

H 15.170017 13.203995 14.327937

H 13.724055 13.079123 14.978691

O 15.915204 15.790930 13.523354

H 16.000256 16.666781 13.062701

H 16.862511 15.583971 13.772194

O 9.588666 16.263979 12.601247

H 10.089409 15.426917 12.773491

H 9.924071 16.493917 11.705737

O 17.100819 18.723963 14.742388

H 16.161188 18.473227 15.010602

H 17.144718 19.682687 14.886946

O 11.673523 14.827628 19.782871

H 12.403632 14.237339 19.414656

H 11.470148 14.482101 20.666228

O 12.425704 11.988285 15.232009

H 12.266649 11.502450 14.377916

H 11.679362 12.610143 15.393807

O 18.397262 17.647328 16.918845

H 17.816771 18.044868 17.611681

H 18.023854 18.003119 16.079133

O 14.834753 14.946455 11.175948

H 15.605259 15.312050 10.672518

H 15.162867 14.992908 12.109680

O 17.888722 13.080839 17.684224

H 17.257743 13.383939 18.372228

H 17.355026 12.478243 17.101357

O 13.263852 17.108240 11.538843

H 13.113796 16.759196 12.464613

H 13.919190 16.432440 11.219057

O 9.690602 11.982691 13.331144

H 10.412314 11.327568 13.154297

H 9.482552 11.857190 14.269545

O 15.694885 11.744491 10.994895

H 16.317813 12.469759 10.668980

H 16.124804 10.924090 10.705400

O 11.040249 16.403335 10.254627

H 10.927619 16.896705 9.428073

H 11.847981 16.797013 10.689430

O 18.722628 13.164604 12.786210

H 19.528266 12.690897 13.041423

H 18.759730 14.052367 13.253367

O 13.899448 11.375280 17.295448

H 13.625045 10.568214 17.756946

H 13.272682 11.475658 16.522410

O 19.142281 15.104857 16.505214

H 18.813312 15.981080 16.828736

H 18.607031 14.393951 16.965730

O 11.057413 17.413035 16.988985

H 10.396902 16.821229 17.420908

H 11.870666 16.846910 16.991935

O 15.844269 16.480976 20.187921

H 16.173586 17.027508 19.432349

H 14.859020 16.602064 20.132777

O 13.208274 17.021845 19.865915

H 12.885041 17.587423 20.584883

H 12.581292 16.245961 19.844774

O 12.040421 19.995518 16.934909

H 12.311876 20.049539 15.994012

H 11.527153 19.156377 16.976690

O 11.007217 17.657374 14.315000

H 10.772746 17.516479 15.268022

H 10.354968 17.185747 13.707406

O 14.273830 19.488681 11.980957

H 13.840935 18.624513 11.710904

H 14.152927 20.086045 11.226661

O 12.986837 11.406694 10.679891

H 13.958470 11.568387 10.748596

H 12.618389 12.298295 10.490911

O 17.604705 13.419553 10.321798

H 18.124960 13.310073 11.153342

H 17.462178 14.386731 10.268313

O 14.114411 18.680177 17.900359

H 13.371235 19.293308 17.618430

H 13.696668 18.022632 18.508188

O 9.740706 15.255754 17.997281

H 10.380387 15.030493 18.729356

H 8.854687 15.129364 18.369181

O 16.315235 12.224475 13.581489

H 17.226555 12.566961 13.392658

H 16.000925 11.929922 12.687991

O 16.586708 18.837211 21.425400

H 15.749093 19.268104 21.654694

H 16.319646 17.903610 21.263690

O 10.780100 14.061532 15.852291

H 10.242923 14.401024 16.618622

H 10.501934 14.581916 15.081494

O 11.991231 10.669818 12.951099

H 12.097678 9.707310 12.985203

H 12.416187 10.964701 12.071303

O 18.476715 15.401752 14.094865

H 18.978893 16.180420 13.807236

H 18.742392 15.250066 15.094872

O 13.686460 13.676082 18.630936

H 13.711143 12.793114 18.168398

H 14.578698 13.755115 19.072626

O 11.242105 14.127237 13.038181

H 11.635761 14.024199 12.127346

H 10.670436 13.318706 13.178246

O 16.666748 18.669954 18.716072

H 16.710671 18.957067 19.661750

H 15.737429 18.846427 18.424049

O 16.587180 18.060614 12.200134

H 16.976583 18.375304 13.057122

H 15.815233 18.665103 12.058624

O 12.838976 19.588115 14.286643

H 12.103532 18.949503 14.105614

H 13.298434 19.735322 13.423664

O 16.462404 11.326017 16.196326

H 15.547334 11.334580 16.554211

H 16.360420 11.549656 15.245141

O 16.117639 13.910200 19.693421

H 16.228278 13.471840 20.551250

H 16.106635 14.892867 19.900791

O 12.327081 14.041481 10.588727

H 13.248086 14.369128 10.733884

H 11.823059 14.817475 10.239060

O 17.062558 16.190145 10.288248

H 16.985654 16.657517 9.442431

H 17.018537 16.902595 10.976296

XYZ Coordinates from $Co(H_2O)_5OH^+$

Co 14.399658 15.474498 15.118145

O 15.617213 15.179535 16.524826

H 16.475679 15.570991 16.289956

O 12.834149 15.233922 16.611923

H 12.101356 14.636230 16.322676

H 13.290614 14.738369 17.347532

O 14.777036 17.613155 15.327048

H 14.655914 17.717664 16.316139

H 14.119299 18.262850 14.942005

O 12.733970 16.123583 13.849822

H 12.172263 15.327932 13.703391

H 12.074435 16.848034 14.088121

O 13.885490 13.660604 14.205733

H 14.684478 13.138708 13.918508

H 13.307649 13.002962 14.666716

O 15.882028 15.487062 13.520527

H 16.199955 16.362791 13.150312

H 16.687665 14.997272 13.781487

O 9.498502 16.575860 12.527001

H 9.843523 15.677524 12.762155

H 9.916876 16.706432 11.646419

O 17.172005 18.750797 14.860641

H 16.294429 18.316659 15.070386

H 16.944992 19.688117 14.751295

O 11.642122 14.711287 19.705675

H 12.407429 14.155534 19.358108

H 11.408576 14.335436 20.568850

O 12.361995 11.760125 15.398131

H 12.133655 11.272767 14.559382

H 11.597369 12.339292 15.623808

O 18.693351 18.262071 17.124736

H 18.031143 18.361314 17.849376

H 18.194392 18.561223 16.330776

O 14.801425 14.836636 11.127334

H 15.588428 15.166761 10.628178

H 15.126617 14.860962 12.063234

O 18.134716 13.666780 17.428185

H 17.468548 13.911607 18.106928

H 17.620024 13.031099 16.867533

O 13.342718 17.096521 11.454738

H 13.089131 16.771242 12.368069

H 13.964237 16.371064 11.179103

O 9.452332 12.003764 13.426305

H 10.209396 11.384785 13.263613

H 9.342044 11.986714 14.390035

O 15.719806 11.632818 10.921319

H 16.420201 12.330202 10.724554

H 16.133463 10.795751 10.658476

O 11.065396 16.432813 10.238404

H 10.952873 16.839266 9.365763

H 11.906334 16.823220 10.606363

O 18.250442 13.837501 13.381049

H 18.761721 13.390902 14.075072

H 18.671615 14.762106 13.312162

O 14.177167 11.401201 17.248607

H 14.028367 10.565589 17.717103

H 13.479894 11.431672 16.532764

O 18.413152 15.883644 15.960146

H 18.543511 16.670632 16.556172

H 18.383592 15.055120 16.521652

O 11.146151 17.368273 16.852167

H 10.462634 16.818525 17.303035

H 11.902778 16.730482 16.799940

O 15.820742 16.379891 20.569320

H 16.221734 16.948629 19.859494

H 14.848372 16.482142 20.403905

O 13.224714 16.857164 19.879150

H 12.815475 17.520486 20.456843

H 12.575432 16.100018 19.850103

O 12.388627 19.831419 16.992864

H 12.672172 19.913945 16.057401

H 11.796377 19.044034 16.980124

O 10.921355 17.970398 14.237050

H 10.742397 17.718901 15.178575

H 10.282546 17.490220 13.619937

O 14.614240 19.300134 12.081477

H 14.093305 18.514946 11.731948

H 14.630359 19.945623 11.357707

O 12.944611 11.425123 10.912802

H 13.919978 11.555481 10.911847

H 12.580090 12.314939 10.709237

O 17.754577 13.289785 10.688209

H 18.104021 13.301767 11.604824

H 17.604063 14.246033 10.513205

O 14.326290 18.269399 17.848531

H 13.642467 18.966970 17.608477

H 13.857302 17.667289 18.479711

O 9.739855 15.282608 17.899498

H 10.374461 15.014529 18.619863

H 8.857784 15.237490 18.299133

O 16.023336 12.265281 13.495474

H 16.757399 12.907159 13.367287

H 15.879062 11.876945 12.590633

O 16.291043 18.938497 21.619254

H 15.407097 19.330428 21.690294

H 16.102245 17.975931 21.573899

O 10.614774 13.766142 15.880719

H 10.113180 14.274167 16.572680

H 10.512092 14.244005 15.036413

O 11.748938 10.626501 13.071168

H 11.739747 9.659053 13.012130

H 12.271079 10.943189 12.256956

O 19.182906 16.229098 13.510118

H 20.151173 16.231367 13.474004

H 18.966328 16.164120 14.492559

O 13.736678 13.651858 18.611423

H 13.831042 12.770743 18.156497

H 14.656352 13.834434 18.949634

O 10.806849 14.260620 13.091260

H 11.367719 14.120479 12.276611

H 10.278738 13.412851 13.185950

O 16.721656 18.386288 18.990853

H 16.647479 18.852378 19.860020

H 15.836571 18.508810 18.552249

O 16.792210 17.715120 12.391497

H 17.246343 18.065490 13.196313

H 16.044471 18.352619 12.244956

O 13.128807 19.474817 14.334966

H 12.284320 19.014912 14.103866

H 13.619709 19.606679 13.486607

O 16.707410 11.730236 16.119156

H 15.832032 11.617253 16.551730

H 16.480042 11.791080 15.167125

O 16.254613 13.983155 19.553221

H 16.430599 13.349852 20.265805

H 16.161161 14.869166 20.012430

O 12.214915 14.049181 10.824808

H 13.157406 14.334778 10.909390

H 11.755203 14.805748 10.383981

O 17.135724 15.965063 10.361681

H 17.280046 16.443280 9.531405

H 17.170496 16.652884 11.075182

XYZ Coordinates from $Cu(H_2O)_5OH^+$

Cu 14.229406 15.440102 14.774109

O 15.883136 14.773358 15.363230

H 16.432657 15.466558 15.783518

O 12.892365 15.248814 16.576584

H 12.140055 14.680692 16.289397

H 13.323170 14.739555 17.320142

O 14.721865 17.342142 15.334642

H 14.592170 17.445986 16.329811

H 14.100299 18.040188 14.953090

O 12.619076 16.057160 13.754817

H 12.025250 15.281473 13.522680

H 11.989259 16.783212 14.076707

O 13.968167 13.675193 13.799942

H 14.863905 13.222615 13.821122

H 13.400361 13.077171 14.357400

O 16.250092 15.176377 12.815234

H 16.557487 16.104433 12.728918

H 16.250487 14.974295 13.809759

O 9.455599 16.594729 12.530747

H 9.823616 15.692768 12.699368

H 9.845246 16.780322 11.646276

O 17.140319 18.592037 14.846295

H 16.311135 18.092092 15.058673

H 16.818591 19.491928 14.676183

O 11.780853 14.641243 19.803010

H 12.543575 14.130433 19.383949

H 11.575857 14.188311 20.635967

O 12.517837 11.991745 15.293329

H 12.220723 11.393336 14.549784

H 11.739034 12.539378 15.544980

O 18.672873 18.471468 17.178950

H 17.998811 18.579689 17.890636

H 18.159199 18.640095 16.356534

O 14.659955 15.041842 10.790864

H 15.389669 15.373333 10.217982

H 15.146272 14.957960 11.673826

O 18.064252 13.694205 17.692794

H 17.446440 13.844901 18.441746

H 17.595329 13.008657 17.149823

O 13.242475 17.195295 11.382475

H 12.984943 16.840948 12.275049

H 13.836659 16.451463 11.061503

O 9.531129 12.128822 13.588024

H 10.230756 11.445902 13.433011

H 9.520679 12.243891 14.552716

O 15.644153 11.752690 11.161735

H 16.312721 12.467245 10.891598

H 15.987080 10.944095 10.750587

O 10.941095 16.559929 10.189228

H 10.787198 17.025186 9.353038

H 11.791817 16.937340 10.548958

O 18.550973 13.478714 13.226406

H 19.370764 13.031481 13.484235

H 18.683783 14.449357 13.462401

O 14.146195 11.408662 17.281023

H 13.854323 10.605579 17.738951

H 13.512268 11.526777 16.516172

O 18.065533 16.010725 16.370536

H 18.377515 16.814204 16.864046

H 18.158317 15.180906 16.921692

O 11.132867 17.320840 16.892868

H 10.491181 16.756403 17.384378

H 11.908625 16.710104 16.809764

O 15.890066 16.486368 20.729394

H 16.285452 17.045401 20.008922

H 14.917299 16.565987 20.552586

O 13.286500 16.829667 20.006449

H 12.830517 17.518663 20.514827

H 12.662408 16.050713 19.995118

O 12.475669 19.724287 17.053476

H 12.747689 19.791009 16.113144

H 11.833289 18.977507 17.047645

O 10.902234 17.916443 14.285690

H 10.709549 17.670658 15.226572

H 10.242314 17.473834 13.663806

O 14.710104 19.227790 12.170107

H 14.142315 18.502084 11.767130

H 14.708071 19.945083 11.516963

O 12.899710 11.526913 11.012898

H 13.868746 11.706577 11.068336

H 12.505489 12.367687 10.699195

O 17.598898 13.402426 10.654292

H 17.992087 13.444868 11.559918

H 17.409140 14.345289 10.451330

O 14.335585 18.031146 17.826417

H 13.679783 18.764874 17.607270

H 13.880389 17.494223 18.525367

O 9.848355 15.195267 18.043227

H 10.503245 14.940812 18.752325

H 8.973655 15.118716 18.454339

O 16.200825 12.217834 13.754627

H 17.016573 12.767655 13.630999

H 16.031428 11.845474 12.852002

O 16.089807 19.172796 21.585197

H 15.167555 19.470347 21.615382

H 16.008833 18.197288 21.639546

O 10.567020 13.837381 15.872442

H 10.167019 14.256260 16.686236

H 10.348519 14.441787 15.143694

O 11.741697 10.651022 13.156133

H 11.786310 9.684872 13.098190

H 12.222278 11.004714 12.330607

O 18.905875 16.009401 13.884481

H 19.850385 16.220366 13.926841

H 18.613379 16.020619 14.846921

O 13.850829 13.722011 18.544135

H 13.926689 12.812550 18.137829

H 14.726737 13.876513 19.000085

O 10.891183 14.294290 12.893352

H 11.357225 14.126390 12.026887

H 10.403673 13.450634 13.132358

O 16.681836 18.452110 19.028494

H 16.512883 18.979900 19.847393

H 15.811458 18.433869 18.545675

O 17.045912 17.774553 12.178021

H 17.436041 17.914261 13.068123

H 16.235295 18.341580 12.184919

O 13.208863 19.286317 14.405012

H 12.334304 18.903620 14.150556

H 13.708782 19.458325 13.565907

O 16.793112 11.611530 16.405719

H 15.892037 11.514520 16.783757

H 16.602263 11.734941 15.449775

O 16.210353 14.072370 19.741245

H 16.312588 13.435460 20.465593

H 16.160028 14.972849 20.188221

O 12.122230 14.123159 10.517293

H 13.057814 14.440329 10.586104

H 11.620680 14.902341 10.175272

O 17.047938 16.072399 10.054927

H 17.406991 16.506319 9.267365

H 17.169012 16.717671 10.795779

XYZ Coordinates from $Ir(H_2O)_5(OH)_2^+$

Ir 14.160813 15.532552 14.968714

O 15.468189 14.790374 16.261221

H 16.270086 15.347793 16.231102

O 12.744315 15.572102 16.406737

H 12.156638 14.808394 16.287243

O 14.684868 17.454019 15.585474

H 14.490736 17.623006 16.575519

H 14.102118 18.141549 15.106401

O 12.637147 16.148197 13.561128

H 12.040764 15.362100 13.370414

H 11.990212 16.871354 13.867034

O 13.710688 13.661102 14.236550

H 14.556498 13.169084 13.971149

H 13.230713 13.033882 14.859595

O 15.712873 15.453236 13.522943

H 16.050853 16.346107 13.151187

H 16.500479 15.006233 13.908737

O 9.542707 16.692943 12.223920

H 9.868990 15.788079 12.453793

H 9.965044 16.816820 11.344701

O 17.095588 18.707457 14.936631

H 16.304216 18.203422 15.250419

H 16.759373 19.611071 14.820483

O 11.951923 14.629918 19.569890

H 12.737905 14.012136 19.339695

H 11.612778 14.284550 20.411222

O 12.362871 11.919485 15.635135

H 12.139349 11.391743 14.815975

H 11.574416 12.493671 15.806246

O 18.672140 18.277036 17.183831

H 17.997629 18.454919 17.881254

H 18.227329 18.611387 16.374536

O 14.803760 14.881344 10.959465

H 15.646020 15.203540 10.549574

H 15.048018 14.814435 11.909643

O 18.198250 13.643331 17.229866

H 17.644823 13.831179 18.022053

H 17.660488 12.960317 16.750430

O 13.334112 17.152649 11.194052

H 13.027540 16.837468 12.090560

H 13.969423 16.422036 10.967956

O 9.479407 12.252087 13.531781

H 10.214396 11.594214 13.461053

H 9.491133 12.509665 14.473046

O 15.695438 11.655005 11.040054

H 16.422071 12.330061 10.857543

H 16.098291 10.799643 10.823289

O 11.088303 16.456769 9.921264

H 10.968655 16.855861 9.046100

H 11.926100 16.855826 10.282686

O 18.101814 13.890120 13.576047

H 18.578710 13.444659 14.295368

H 18.558041 14.796141 13.490271

O 14.308059 11.245433 17.307733

H 14.158233 10.345102 17.634907

H 13.544943 11.428902 16.695664

O 18.020302 15.982542 15.997727

H 18.289116 16.722934 16.608662

H 18.198648 15.107003 16.457484

O 10.967333 17.394085 16.594424

H 10.351539 16.871596 17.151309

H 11.747866 16.725364 16.492737

O 16.064823 16.407958 20.438202

H 16.362567 17.058827 19.749996

H 15.076533 16.462316 20.369181

O 13.398449 16.781633 20.043451

H 12.964524 17.312431 20.729353

H 12.802934 15.986670 19.895899

O 12.257822 19.737622 16.993499

H 12.621118 19.849450 16.092199

H 11.672945 18.940722 16.900035

O 10.911548 18.004502 14.049172

H 10.654450 17.742329 14.977576

H 10.287207 17.586156 13.377078

O 14.621178 19.263968 12.059897

H 14.090778 18.544619 11.601016

H 14.704773 19.989265 11.421461

O 12.920423 11.474765 11.119098

H 13.894078 11.602774 11.068969

H 12.546211 12.300157 10.745162

O 17.769575 13.263547 10.868795

H 18.059205 13.306519 11.804989

H 17.647978 14.213003 10.643887

O 14.189336 18.266695 17.966000

H 13.468385 18.919517 17.691813

H 13.786624 17.691111 18.670249

O 9.913464 15.224664 17.942030

H 10.667710 14.982568 18.550977

H 9.109107 15.072035 18.461181

O 15.851274 12.352670 13.595573

H 16.603884 12.979076 13.493525

H 15.754828 11.943680 12.689573

O 16.349521 18.920178 21.646622

H 15.436237 19.199747 21.813543

H 16.267134 17.946796 21.546801

O 10.357328 13.740143 15.792316

H 10.080075 14.253103 16.606344

H 10.462579 14.414780 15.100237

O 11.749472 10.764585 13.331102

H 11.698311 9.799438 13.257425

H 12.255700 11.069797 12.506756

O 19.076077 16.273531 13.633105

H 20.040923 16.262608 13.723577

H 18.735254 16.265795 14.582223

O 13.979968 13.127504 19.227233

H 14.056191 12.415739 18.546455

H 14.891008 13.504808 19.286782

O 10.873448 14.372378 12.766450

H 11.372858 14.150864 11.933033

H 10.354573 13.548672 13.013857

O 16.646801 18.614782 18.961257

H 16.567291 18.992032 19.872218

H 15.733512 18.657573 18.574165

O 16.672034 17.608239 12.494165

H 17.143320 17.978964 13.280730

H 15.961138 18.282170 12.302156

O 13.257053 19.259080 14.388683

H 12.374067 18.900107 14.119069

H 13.739800 19.440265 13.541866

O 16.800256 11.567165 16.095737

H 15.943668 11.468266 16.569187

H 16.507575 11.712035 15.171510

O 16.570783 14.002792 19.468671

H 16.922599 13.478692 20.205119

H 16.449015 14.921601 19.853723

O 12.228674 14.050223 10.464968

H 13.159600 14.352404 10.583654

H 11.762888 14.818799 10.052865

O 17.223560 15.942403 10.416108

H 17.428176 16.430959 9.604536

H 17.202853 16.619258 11.138333

XYZ Coordinates from $Ni(H_2O)_5OH^+$

Ni 14.416255 15.406726 14.641543

O 15.976823 14.633797 15.740141

H 16.637839 15.283328 16.118964

O 13.164011 15.216388 16.311424

H 12.349311 14.691752 16.088462

H 13.564889 14.725757 17.088124

O 14.843508 17.334683 15.303720

H 14.677573 17.451392 16.288097

H 14.230740 18.024380 14.894964

O 12.709246 16.030923 13.624501

H 12.112560 15.254149 13.458350

H 12.088949 16.738264 13.984403

O 14.003586 13.501749 13.961556

H 14.830305 12.954162 13.862601

H 13.442240 12.930865 14.546904

O 15.574412 15.584359 13.012966

H 15.913746 16.481902 12.823859

H 16.782379 14.789328 13.225162

O 9.446370 16.556686 12.587430

H 9.810423 15.652167 12.753407

H 9.784735 16.722659 11.677838

O 17.211466 18.530330 14.796690

H 16.360377 18.063945 15.030552

H 16.948225 19.463739 14.745485

O 11.861789 14.657788 19.685063

H 12.629781 14.175595 19.239036

H 11.673784 14.167567 20.500798

O 12.502566 11.775792 15.397870

H 12.250262 11.258052 14.583831

H 11.754293 12.384698 15.597971

O 18.689424 18.307388 17.184115

H 18.020877 18.499094 17.885274

H 18.209973 18.493202 16.345618

O 14.532746 15.131641 10.771875

H 15.292067 15.399203 10.211110

H 14.937986 15.238930 11.736193

O 18.207890 13.560270 17.873498

H 17.538113 13.733314 18.573087

H 17.751081 12.914516 17.275585

O 13.108392 17.278705 11.250398

H 12.916552 16.899382 12.149947

H 13.708399 16.547496 10.902390

O 9.544743 11.999299 13.479436

H 10.288809 11.360133 13.330215

H 9.452311 12.025124 14.444624

O 15.623013 11.763426 11.121655

H 16.262568 12.516926 10.900596

H 15.959168 11.011317 10.609693

O 10.791855 16.528668 10.169577

H 10.581708 16.978629 9.337328

H 11.639219 16.948079 10.491249

O 17.582841 14.140729 13.432843

H 16.547076 14.174834 15.087514

H 18.352635 14.719241 13.698823

O 14.265148 11.388498 17.302767

H 14.026934 10.612625 17.833062

H 13.592904 11.422871 16.563596

O 18.127911 15.826577 16.551444

H 18.391612 16.689266 16.986781

H 18.299281 15.030718 17.142051

O 11.357693 17.292727 16.834734

H 10.719593 16.704690 17.307412

H 12.135091 16.700321 16.696957

O 15.921264 16.497942 20.704704

H 16.323838 17.059923 19.990813

H 14.949954 16.585930 20.522891

O 13.325513 16.864395 19.968449

H 12.852461 17.535896 20.484437

H 12.714239 16.075873 19.931376

O 12.573166 19.761777 16.957476

H 12.855863 19.806920 16.018196

H 11.957455 18.993806 16.963120

O 10.986835 17.898670 14.241269

H 10.846865 17.644463 15.187756

H 10.303330 17.445819 13.652888

O 14.551771 19.359805 11.960855

H 13.989305 18.616288 11.583277

H 14.465331 20.095545 11.335056

O 12.887621 11.557525 10.953411

H 13.855836 11.744200 11.003438

H 12.477617 12.406395 10.681235

O 17.511535 13.541658 10.782789

H 17.690051 13.712547 11.741823

H 17.342726 14.450448 10.442910

O 14.401740 18.059326 17.794868

H 13.765366 18.800606 17.551576

H 13.925960 17.541873 18.494645

O 9.950422 15.238698 17.925493

H 10.579884 14.962530 18.649718

H 9.064535 15.225591 18.318461

O 16.198920 11.937552 13.749436

H 16.868130 12.663269 13.628767

H 16.009310 11.651311 12.819528

O 16.113940 19.180055 21.580772

H 15.190614 19.474919 21.604968

H 16.034829 18.204087 21.628640

O 10.843197 13.876649 15.821542

H 10.342574 14.310236 16.565519

H 10.562106 14.314389 14.999435

O 11.817071 10.621146 13.109812

H 11.838306 9.659124 12.994872

H 12.272324 11.008559 12.283252

O 19.484050 15.810351 14.271067

H 20.298018 15.311748 14.436740

H 19.041681 15.845181 15.166181

O 13.937215 13.775299 18.406027

H 14.014838 12.843162 18.054085

H 14.791761 13.927216 18.901796

O 10.849153 14.233556 12.917202

H 11.307942 14.113818 12.037695

H 10.365561 13.374403 13.104425

O 16.724003 18.479392 19.025152

H 16.550796 18.999265 19.848244

H 15.856312 18.460403 18.535737

O 16.902151 17.969709 12.103467

H 17.211324 18.120162 13.027307

H 16.104908 18.548956 12.021575

O 13.302173 19.244739 14.333158

H 12.420954 18.851648 14.114845

H 13.729516 19.468916 13.465929

O 16.906178 11.606097 16.433129

H 15.992213 11.535644 16.787237

H 16.752152 11.601770 15.466337

O 16.227583 14.070447 19.748398

H 16.227882 13.441446 20.486969

H 16.184822 14.979685 20.179469

O 12.057066 14.139296 10.528166

H 12.986205 14.492103 10.573692

H 11.524452 14.890789 10.172783

O 16.959884 16.155197 10.088640

H 17.377220 16.587969 9.329638

H 17.032390 16.804399 10.835329

XYZ Coordinates from $Fe(H_2O)_5OH^{2+}$

Fe 14.582802 15.381816 14.810292

O 16.087757 14.909465 15.730790

H 16.886651 15.406598 16.084964

O 13.227735 15.170529 16.367191

H 12.364050 14.724548 16.112658

H 13.571579 14.655051 17.174485

O 14.763214 17.407622 15.287812

H 14.641038 17.574575 16.287457

H 14.131370 18.084449 14.857247

O 12.869681 15.936533 13.695457

H 12.302057 15.146272 13.434552

H 12.187802 16.611476 14.041134

O 14.210946 13.520869 14.066324

H 15.018811 12.916803 13.982793

H 13.510172 12.945812 14.508109

O 15.655214 15.686992 13.009807

H 15.894519 16.636007 12.726054

H 16.535218 15.229325 13.126293

O 9.468017 16.358193 12.664793

H 9.959117 15.503702 12.747475

H 9.698513 16.603462 11.740696

O 17.170668 18.624064 14.767308

H 16.334094 18.151323 15.014749

H 16.927678 19.562065 14.839541

O 11.873522 14.680095 19.768827

H 12.607993 14.166974 19.309350

H 11.727603 14.241102 20.621534

O 12.493420 11.924414 15.215447

H 12.220485 11.344280 14.446035

H 11.733805 12.511248 15.437187

O 18.716125 18.237293 17.129014

H 18.056430 18.418685 17.842373

H 18.227394 18.451209 16.304786

O 14.513857 15.148652 10.575352

H 15.296391 15.532634 10.111134

H 14.845829 15.090125 11.502700

O 18.126164 13.559249 17.961215

H 17.460860 13.750900 18.660265

H 17.652054 12.920857 17.370796

O 12.987852 17.247680 11.266605

H 12.901615 16.859415 12.173975

H 13.600153 16.579903 10.850578

O 9.619998 12.094205 13.379079

H 10.287241 11.374076 13.249920

H 9.413000 12.059876 14.325602

O 15.598672 11.861743 11.158323

H 16.194792 12.648543 10.964647

H 15.966702 11.148614 10.612648

O 10.660731 16.459880 10.178550

H 10.442324 16.946767 9.369258

H 11.493173 16.884011 10.522485

O 17.722134 14.168441 13.737275

H 17.210382 14.308020 14.582285

H 18.666035 14.472342 13.951970

O 14.084791 11.370118 17.263845

H 13.785886 10.587005 17.751133

H 13.464341 11.459404 16.487903

O 18.422993 15.717197 16.503203

H 18.573955 16.616932 16.924614

H 18.405078 14.968910 17.178011

O 11.324859 17.284506 16.925623

H 10.708058 16.678069 17.405766

H 12.133751 16.739463 16.822673

O 15.871727 16.521771 20.682823

H 16.296075 17.051160 19.957308

H 14.905439 16.636625 20.489242

O 13.306348 16.928608 19.872718

H 12.825146 17.607525 20.371611

H 12.712303 16.126487 19.876780

O 12.506240 19.791048 16.971421

H 12.745434 19.835215 16.020234

H 11.891282 19.024686 17.007451

O 11.051255 17.660473 14.284817

H 10.874851 17.485299 15.243995

H 10.339929 17.234839 13.704181

O 14.293691 19.448120 11.894212

H 13.757471 18.684042 11.527325

H 14.214838 20.170311 11.251847

O 12.874235 11.527039 10.898497

H 13.827719 11.764818 10.983520

H 12.436875 12.343192 10.579630

O 17.447446 13.726691 10.908546

H 17.669831 13.846505 11.851151

H 17.327018 14.646341 10.578456

O 14.402666 18.146968 17.733747

H 13.742771 18.880578 17.518382

H 13.943186 17.599377 18.424092

O 9.958133 15.206481 18.012883

H 10.572715 14.939139 18.752829

H 9.059859 15.168852 18.375263

O 16.261122 11.914663 13.755934

H 16.986079 12.580472 13.669409

H 16.045154 11.685218 12.812487

O 16.230422 19.196938 21.524258

H 15.329005 19.552619 21.557135

H 16.088034 18.229054 21.587970

O 10.878837 14.036114 15.826194

H 10.388218 14.361543 16.634183

H 10.491041 14.519234 15.078072

O 11.800993 10.584125 13.054536

H 11.871675 9.619644 12.994376

H 12.248452 10.956076 12.218343

O 20.030342 15.085570 14.507970

H 20.560579 14.342017 14.833477

H 19.544959 15.400246 15.324213

O 13.894622 13.737156 18.421271

H 13.938509 12.802768 18.065030

H 14.748920 13.875130 18.929160

O 11.140163 14.187174 12.838846

H 11.462888 14.052839 11.904777

H 10.605941 13.373174 13.082911

O 16.762262 18.442594 18.975858

H 16.630527 18.976064 19.799236

H 15.897594 18.488910 18.489885

O 16.513770 17.988657 12.197885

H 16.953574 18.287528 13.034167

H 15.762724 18.628613 12.057032

O 13.191155 19.239813 14.330440

H 12.329180 18.790015 14.138759

H 13.561415 19.512488 13.451521

O 16.766162 11.615047 16.536023

H 15.840852 11.509925 16.848351

H 16.662623 11.596551 15.565425

O 16.129612 14.064102 19.809746

H 16.135812 13.454487 20.564375

H 16.088214 14.987220 20.214198

O 12.000859 14.089346 10.294343

H 12.912933 14.463987 10.303006

H 11.423734 14.853150 10.046987

O 16.896347 16.303503 10.071383

H 17.287823 16.696316 9.276943

H 16.923500 17.006605 10.764002

XYZ Coordinates from $Co(H_2O)_5OH^{2+}$

Co 14.429979 15.335151 14.680512

O 15.849145 14.675686 15.656140

H 16.379065 15.423709 16.018530

O 13.280490 15.169226 16.202876

H 12.390940 14.738416 16.001983

H 13.679829 14.635058 16.977036

O 14.851140 17.128716 15.230444

H 14.701584 17.272518 16.233112

H 14.261667 17.855097 14.807677

O 12.887036 15.923166 13.634344

H 12.298083 15.138839 13.379097

H 12.224493 16.596282 14.031488

O 14.070580 13.576591 14.047222

H 14.898474 12.988211 14.004512

H 13.436545 13.022587 14.606565

O 15.522024 15.539763 13.110980

H 15.831511 16.490281 12.899402

H 16.388030 15.027269 13.262797

O 9.524084 16.456930 12.642446

H 9.975167 15.581030 12.725596

H 9.760099 16.687642 11.715722

O 17.229558 18.598215 14.880651

H 16.492359 17.997781 15.140189

H 16.812026 19.475944 14.882724

O 11.967972 14.625551 19.742810

H 12.691008 14.157479 19.217779

H 11.846559 14.112095 20.556864

O 12.483847 11.949477 15.317429

H 12.233225 11.384120 14.528860

H 11.707449 12.515259 15.533036

O 18.807378 18.435153 17.252319

H 18.131146 18.593187 17.953817

H 18.325796 18.650110 16.425932

O 14.529321 15.099131 10.569182

H 15.354796 15.468730 10.169063

H 14.788934 15.009934 11.515462

O 18.091908 13.683437 17.709187

H 17.466369 13.844540 18.450635

H 17.632164 12.977625 17.187935

O 13.052582 17.251580 11.203207

H 12.937355 16.869643 12.108091

H 13.647694 16.560205 10.801256

O 9.638388 12.133998 13.376195

H 10.317428 11.419122 13.280641

H 9.418995 12.128820 14.320522

O 15.558156 11.818251 11.191570

H 16.195413 12.580248 11.023656

H 15.932408 11.080946 10.683718

O 10.694010 16.505395 10.141202

H 10.463326 16.990713 9.334296

H 11.539052 16.920006 10.462916

O 17.553029 14.121925 13.849193

H 16.964949 14.264454 14.691007

H 18.381936 14.682064 13.987260

O 14.126294 11.329945 17.309321

H 13.787776 10.602718 17.853859

H 13.483713 11.425637 16.553961

O 18.004104 16.028569 16.476892

H 18.352110 16.816979 16.974751

H 18.144245 15.182687 16.999054

O 11.385259 17.303961 16.920709

H 10.780895 16.702927 17.423738

H 12.188421 16.758445 16.795984

O 15.957042 16.504538 20.734170

H 16.388394 17.063076 20.035740

H 14.994160 16.618181 20.526114

O 13.398152 16.863702 19.882020

H 12.890055 17.574456 20.303774

H 12.808776 16.058428 19.912057

O 12.755046 19.713642 16.973825

H 12.985691 19.735837 16.019740

H 12.061719 19.018880 17.015650

O 11.140991 17.673603 14.285167

H 10.938729 17.508863 15.241177

H 10.416272 17.288712 13.689980

O 14.463114 19.366551 11.908000

H 13.894072 18.649464 11.500148

H 14.447498 20.116880 11.294051

O 12.839253 11.542644 10.918179

H 13.798374 11.764244 10.983404

H 12.410267 12.356673 10.584014

O 17.475760 13.608488 11.029052

H 17.630807 13.749331 11.983534

H 17.373728 14.523363 10.678404

O 14.517688 17.888657 17.660895

H 13.904870 18.668064 17.464026

H 14.051897 17.398671 18.390512

O 10.035335 15.246190 18.050194

H 10.657849 14.948109 18.772882

H 9.140810 15.207139 18.421627

O 16.076048 11.918431 13.815970

H 16.838979 12.540483 13.747967

H 15.894287 11.671440 12.869143

O 15.960923 19.244230 21.495088

H 15.015817 19.459552 21.467237

H 15.960744 18.273150 21.618602

O 10.889854 14.092662 15.835641

H 10.430798 14.427370 16.660388

H 10.457674 14.553125 15.097457

O 11.839953 10.636865 13.124238

H 11.896421 9.671559 13.061576

H 12.259351 11.002745 12.270716

O 19.503278 15.791010 14.329761

H 20.304658 15.331467 14.622964

H 19.008028 15.971328 15.183771

O 13.945796 13.777235 18.276428

H 13.998389 12.819418 17.987300

H 14.790416 13.940791 18.792510

O 11.127808 14.233932 12.791142

H 11.446971 14.090599 11.857125

H 10.603851 13.416629 13.049231

O 16.773901 18.461677 19.025818

H 16.501849 19.006053 19.806153

H 15.954942 18.369997 18.472224

O 16.557525 17.780459 12.363540

H 17.025223 18.113462 13.170138

H 15.858352 18.463630 12.165547

O 13.405119 19.076297 14.342588

H 12.502402 18.711260 14.157406

H 13.763395 19.381725 13.468265

O 16.805619 11.529166 16.535154

H 15.895323 11.440674 16.892146

H 16.641003 11.525903 15.572870

O 16.181637 14.110561 19.670023

H 16.208554 13.449901 20.380122

H 16.157542 15.000857 20.141321

O 11.991043 14.107510 10.247885

H 12.909018 14.466036 10.253828

H 11.425388 14.880160 10.002480

O 16.961376 16.181355 10.191987

H 17.378770 16.603007 9.425896

H 16.986571 16.850001 10.919708

XYZ Coordinates from $Ir(H_2O)_5OH^{2+}$

Ir 14.412809 15.363102 14.683905

O 15.964350 14.638349 15.735867

H 16.504008 15.360906 16.129502

O 13.209202 15.182644 16.355437

H 12.341003 14.715719 16.139978

H 13.627194 14.641762 17.121624

O 14.840276 17.296087 15.324698

H 14.666008 17.436250 16.325077

H 14.233540 17.999961 14.883429

O 12.748434 15.957454 13.518580

H 12.142794 15.169344 13.321242

H 12.108470 16.652786 13.920754

O 14.053617 13.460449 13.990662

H 14.895166 12.884825 13.949455

H 13.420437 12.913126 14.558819

O 15.603175 15.554318 13.003108

H 15.907126 16.508867 12.787106

H 16.470948 15.042143 13.197794

O 9.469103 16.500098 12.581279

H 9.857302 15.600340 12.703537

H 9.772325 16.698042 11.665622

O 17.247889 18.626237 14.843071

H 16.449686 18.120790 15.130894

H 16.938060 19.547203 14.829619

O 11.923768 14.652116 19.755176

H 12.657715 14.156966 19.277306

H 11.769577 14.174385 20.585430

O 12.468361 11.892628 15.322173

H 12.189766 11.327445 14.543194

H 11.709244 12.477913 15.548936

O 18.799035 18.432261 17.242102

H 18.103530 18.593450 17.923835

H 18.332553 18.619345 16.400409

O 14.578875 15.108469 10.482530

H 15.383279 15.481872 10.050453

H 14.882631 15.024375 11.416097

O 18.164221 13.627687 17.746980

H 17.513691 13.805705 18.462604

H 17.696669 12.953145 17.193660

O 13.083962 17.255728 11.129702

H 12.926054 16.847598 12.019676

H 13.691477 16.574350 10.731430

O 9.571892 12.086343 13.412585

H 10.262471 11.384869 13.299641

H 9.394605 12.092292 14.365810

O 15.596508 11.832371 11.163594

H 16.217763 12.604304 10.991471

H 15.958341 11.110778 10.625262

O 10.716169 16.502773 10.110706

H 10.460475 16.959998 9.294958

H 11.569129 16.928902 10.395222

O 17.556630 14.143012 13.843961

H 16.991554 14.278150 14.720137

H 18.433378 14.618137 13.997705

O 14.156621 11.345307 17.303342

H 13.855892 10.596171 17.840732

H 13.503958 11.416202 16.554438

O 18.163135 15.969097 16.498574

H 18.459921 16.779706 16.993324

H 18.284262 15.138171 17.046075

O 11.300687 17.301927 16.894088

H 10.686982 16.707937 17.393271

H 12.116555 16.763849 16.822697

O 15.969213 16.493580 20.672343

H 16.370811 17.061263 19.963278

H 14.998060 16.593512 20.496880

O 13.379716 16.876347 19.930460

H 12.912369 17.557471 20.439410

H 12.776370 16.081092 19.930409

O 12.584517 19.758713 16.974198

H 12.843693 19.795474 16.028865

H 11.926787 19.028533 16.994942

O 11.059595 17.713506 14.260063

H 10.877107 17.512291 15.214052

H 10.334825 17.333939 13.662905

O 14.439108 19.365705 11.932984

H 13.889469 18.643969 11.503902

H 14.418814 20.122788 11.327205

O 12.863635 11.550439 10.969097

H 13.822741 11.773376 11.024763

H 12.433170 12.356790 10.619302

O 17.492483 13.652349 10.994079

H 17.677712 13.764445 11.946017

H 17.401510 14.575488 10.661868

O 14.416565 18.040154 17.741126

H 13.769255 18.785223 17.525911

H 13.968369 17.519035 18.460137

O 9.974534 15.231985 18.044206

H 10.609878 14.954780 18.762989

H 9.086067 15.177028 18.428062

O 16.128844 11.910145 13.784515

H 16.854552 12.581520 13.737633

H 15.971977 11.665537 12.833057

O 16.117281 19.178053 21.556605

H 15.188268 19.452950 21.594499

H 16.061551 18.201469 21.614261

O 10.875878 14.037578 15.868638

H 10.382047 14.379417 16.669095

H 10.488296 14.499799 15.106662

O 11.785479 10.611674 13.124501

H 11.842940 9.647893 13.041533

H 12.235854 10.994603 12.295074

O 19.679374 15.578338 14.386108

H 20.405054 15.012442 14.690452

H 19.193698 15.811827 15.232375

O 13.940733 13.753255 18.356718

H 13.995705 12.807391 18.029595

H 14.804203 13.912476 18.843542

O 10.994334 14.216599 12.789647

H 11.361508 14.066793 11.875258

H 10.494009 13.384632 13.050435

O 16.751278 18.498608 19.005674

H 16.552109 19.007706 19.830568

H 15.898355 18.471999 18.499223

O 16.583412 17.825155 12.318802

H 17.027928 18.141545 13.146564

H 15.859828 18.489240 12.143841

O 13.324977 19.133292 14.352016

H 12.436763 18.738665 14.160669

H 13.700191 19.403782 13.472461

O 16.846709 11.513023 16.510300

H 15.934156 11.454093 16.866239

H 16.683776 11.505977 15.547883

O 16.219044 14.082175 19.670277

H 16.256207 13.441178 20.397670

H 16.194239 14.985852 20.116286

O 12.004953 14.109797 10.294555

H 12.925278 14.459502 10.281399

H 11.446098 14.877009 10.018320

O 17.010894 16.228086 10.130089

H 17.493292 16.615019 9.384259

H 17.028556 16.907366 10.845952

XYZ Coordinates from $Cu(H_2O)_6^+$

Cu 14.547172 15.262136 14.778896

O 15.640289 14.881227 16.389572

H 16.545572 15.286294 16.314553

H 15.836612 13.907647 16.403967

O 12.677959 15.501727 16.985030

H 12.252469 14.886744 16.361195

H 13.237314 14.914143 17.547887

O 14.998681 17.939148 15.439204

H 14.740122 17.939047 16.395380

H 14.269770 18.467861 15.018694

O 12.883222 15.923264 13.800557

H 12.327162 15.141125 13.545546

H 12.218366 16.565923 14.198552

O 14.238122 13.407543 13.499785

H 15.133365 13.023773 13.616445

H 13.682599 12.909998 14.144333

O 16.045047 15.654703 13.110035

H 16.143258 16.601412 12.834257

H 16.977272 15.405638 13.329798

O 9.559671 16.424194 12.618614

H 10.010820 15.548333 12.718248

H 9.874102 16.674194 11.722138

O 17.314627 18.870641 14.742375

H 16.408625 18.541498 15.066709

H 17.235946 19.837691 14.761808

O 11.640723 14.706362 19.768210

H 12.414630 14.158545 19.425805

H 11.392202 14.314078 20.619891

O 12.608635 12.041857 15.226924

H 12.272907 11.425342 14.516829

H 11.869575 12.663711 15.429678

O 18.656319 18.084865 17.032087

H 17.963511 18.280689 17.709269

H 18.263675 18.438992 16.199293

O 14.756287 15.081402 10.839164

H 15.520382 15.480357 10.357634

H 15.128201 15.028045 11.760599

O 18.201556 13.528146 17.946731

H 17.548169 13.753795 18.650696

H 17.662515 12.952628 17.357665

O 13.195750 17.162659 11.473959

H 13.042673 16.760859 12.380474

H 13.838218 16.497055 11.099927

O 9.548324 12.157494 13.590692

H 10.225907 11.448419 13.472736

H 9.659970 12.446995 14.515424

O 15.643146 11.667225 11.193002

H 16.226132 12.445030 10.924960

H 15.975466 10.933535 10.652348

O 10.955937 16.461518 10.222436

H 10.836528 16.961798 9.401000

H 11.780066 16.841548 10.641175

O 18.939110 12.905989 12.819009

H 19.805448 12.489945 12.937907

H 18.993487 13.808758 13.245074

O 13.980152 11.342265 17.316726

H 13.734870 10.475967 17.674117

H 13.388522 11.502422 16.519022

O 18.197030 15.614779 16.330711

H 18.424182 16.522732 16.711179

H 18.348727 14.893325 17.012260

O 10.877855 17.440897 17.092175

H 10.184819 16.846777 17.454706

H 11.665693 16.833130 17.125223

O 15.780793 16.484100 20.517042

H 16.146646 17.020945 19.765392

H 14.799306 16.589037 20.412214

O 13.142158 16.910867 19.995376

H 12.723611 17.518667 20.624791

H 12.527372 16.126711 19.940203

O 12.184780 19.897019 17.021079

H 12.455436 19.925271 16.077455

H 11.558539 19.140853 17.064955

O 11.043862 17.646993 14.427499

H 10.756577 17.525447 15.370539

H 10.372062 17.253335 13.791217

O 14.328412 19.461243 12.037939

H 13.862875 18.625726 11.729091

H 14.213726 20.101954 11.319187

O 12.900576 11.454365 11.014669

H 13.867266 11.625997 11.121306

H 12.539874 12.304969 10.687155

O 17.477654 13.450253 10.584420

H 18.121067 13.257932 11.305080

H 17.409994 14.427738 10.561470

O 14.160680 18.407782 17.961768

H 13.455445 19.060401 17.672981

H 13.708588 17.798049 18.594355

O 9.735956 15.089391 17.909372

H 10.414105 14.901549 18.616000

H 8.886364 14.824187 18.293768

O 16.593485 11.888139 13.708710

H 17.485431 12.234554 13.453907

H 16.207342 11.596334 12.845841

O 16.520027 19.004302 21.494722

H 15.680741 19.466367 21.643609

H 16.250800 18.060133 21.474644

O 10.589870 13.849138 15.702434

H 10.198745 14.268456 16.521428

H 10.513017 14.526625 15.010621

O 11.730722 10.622427 13.172952

H 11.802511 9.656526 13.154308

H 12.201655 10.955933 12.333835

O 18.651135 15.290901 13.822979

H 19.168292 16.047207 13.505884

H 18.564148 15.411610 14.827051

O 13.741444 13.636483 18.689389

H 13.784681 12.747361 18.249467

H 14.641277 13.768688 19.091442

O 11.100085 14.184164 12.837760

H 11.536860 14.041850 11.955182

H 10.538478 13.378328 13.027819

O 16.659349 18.435022 18.839835

H 16.698973 18.893353 19.715894

H 15.740615 18.604927 18.496719

O 16.701152 18.130950 12.207177

H 17.088836 18.409192 13.077583

H 15.889068 18.691257 12.114831

O 13.004743 19.468483 14.406372

H 12.248709 18.851839 14.238434

H 13.412696 19.651530 13.525389

O 16.325017 12.261662 16.365966

H 15.541762 11.782892 16.734493

H 16.361691 12.022837 15.407274

O 16.190030 14.001327 19.776892

H 16.283027 13.451478 20.570594

H 16.093123 14.941852 20.123572

O 12.253961 14.069989 10.403221

H 13.170080 14.426696 10.502487

H 11.711059 14.848308 10.125970

O 17.131694 16.232733 10.291457

H 17.447639 16.649345 9.475948

H 17.086775 16.962113 10.959225

4.5 Molecular structure of initial Ir_3O_{14} configurations for protocol (iii) calculations using PBE

XYZ Coordinates from $Ir_3(O\mu_2)_4(OH\mu_1)_5(H_2O)_5^+$

Ir 4.569383 6.604139 5.802784

O 5.455313 4.966650 5.246513

Ir 7.216449 4.409009 5.504088

O 7.842623 5.880534 4.355802

Ir 7.972390 7.334689 5.694408

O 6.736093 2.966386 6.803500

H 5.793191 3.371529 7.929992

O 9.216049 3.820859 5.732538

H 9.616985 2.990135 5.270898

O 2.860164 5.396370 5.882726

H 1.967708 5.752686 5.454288

O 3.392517 8.177208 6.351048

H 3.767950 8.718828 7.103963

O 8.274514 8.727820 7.226531

H 7.457947 9.315068 7.482575

O 8.203085 9.022654 4.432657

H 6.282825 2.212044 6.343646

H 2.988157 4.443370 5.506045

H 8.670067 8.271294 8.041088

H 7.441586 9.678019 4.516117

O 6.101233 7.710165 5.752008

O 7.673567 5.798599 6.803691

H 8.386901 8.725408 3.451393

O 9.903746 7.149665 5.591372

H 10.381776 7.880884 6.073090

O 6.882725 2.908370 4.047374

H 7.530053 3.054043 3.276732

O 4.830769 6.104504 7.688898

H 4.524777 6.746150 8.392932

O 4.103617 6.974800 3.885833

H 4.446496 6.241111 3.285866

H 5.838183 2.899486 3.623336

O 11.261940 8.777038 7.313880

H 12.063451 9.243454 7.666949

H 10.865102 8.311307 8.098242

O 8.417192 8.958947 10.949608

H 8.799687 9.176275 11.821771

H 8.288416 9.875407 10.432569

O 4.109510 9.547049 8.561329

H 4.113750 8.769422 9.220462

H 3.413164 10.165927 8.854196

O 6.475120 10.591907 1.727533

H 7.101777 10.223118 1.034102

H 7.049651 10.839216 2.476450

O 1.819926 9.783211 4.891749

H 2.478379 10.416124 4.502615

H 2.420901 9.199548 5.445385

O 8.191846 11.120461 9.655686

H 7.477706 11.005871 8.972666

H 9.027691 11.170352 9.080169

O 4.508635 9.002096 2.288441

H 5.416049 9.437205 2.135879

H 4.558737 8.283440 3.003345

O 6.767621 7.126517 -0.493030

H 7.065653 6.604013 0.293723

H 5.788517 7.236028 -0.321186

O 1.009501 7.565171 7.255903

H 1.952300 7.827568 6.950295

H 0.588832 8.398243 7.543449

O 8.707121 3.131265 2.148399

H 9.497921 3.459790 2.624779

H 8.843448 2.136497 2.147990

O 9.080410 0.561028 2.813074

H 8.385993 0.232328 3.453885

H 9.413158 -0.226471 2.340658

O 4.619506 2.868659 3.094864

H 3.956352 2.937415 3.845512

H 4.565282 3.750769 2.606998

O 9.152950 0.739307 7.030457

H 9.340428 -0.145085 7.400580

H 8.428110 0.585617 6.347350

O 4.835770 5.242323 1.983909

H 5.830270 5.368870 1.901080

H 4.453210 5.843238 1.277571

O 9.457522 3.242768 8.188098

H 9.354826 3.667255 6.768012

H 9.167176 2.312105 7.998651

O 5.004361 0.822504 5.925803

H 4.405338 1.067119 6.688266

H 4.468137 1.082597 5.149613

O 6.430319 10.407121 7.677552

H 5.532575 10.103288 8.026327

H 6.297055 10.686596 6.724959

O 8.025023 9.307461 0.021212

H 8.526906 9.620931 -0.755207

H 7.496201 8.467687 -0.276393

O 6.371297 10.902769 4.985669

H 6.764934 11.786983 4.838116

H 5.446823 10.965771 4.562964

O 5.101132 3.727689 8.611357

H 4.953897 4.674226 8.241058

H 6.664243 4.054288 9.502262

O 0.619887 6.178105 5.004320

H 0.496837 6.766609 5.807622

H 0.919349 6.818243 4.230526

O 3.991188 11.055127 3.815376

H 3.949654 11.858864 3.259064

H 4.076362 10.285428 3.149759

O 6.840457 6.835411 10.772755

H 7.384935 7.681794 10.873194

H 6.742344 6.510157 11.690421

O 7.570050 4.297408 9.828091

H 8.724625 3.654341 8.745851

H 7.468809 5.265387 10.019910

O 9.661678 7.602111 9.139240

H 9.309077 8.081747 9.966421

H 9.513257 6.647004 9.283945

O 4.333603 7.419975 9.993402

H 5.280124 7.245545 10.286577

H 3.839337 6.555981 10.233037

O 3.250656 5.080236 10.433161

H 2.583970 5.079692 9.666096

H 3.961458 4.492252 10.092023

O 1.602012 5.154741 8.368339

H 1.279583 6.082008 8.198795

H 2.184026 5.034364 7.576688

O 3.331073 2.114869 7.568199

H 3.935809 2.783759 8.057948

H 2.638467 1.848395 8.202968

O 1.597642 7.722226 3.229350

H 2.533740 7.394332 3.389042

H 1.613233 8.584700 3.763233

O 4.193975 7.260728 0.343122

H 4.214318 8.013729 1.026899

H 3.389547 7.401110 -0.192827

O 2.827554 2.958929 5.102247

H 1.834018 3.038443 4.884017

H 2.899834 2.603414 6.044297

O 12.895639 10.580022 8.555593

H 13.241299 10.343604 9.439580

H 13.649381 11.025603 8.119485

O 9.010412 8.131914 2.194019

H 8.781506 8.641368 1.359471

H 8.628536 7.238530 2.013265

O 10.237743 11.241589 7.984421

H 10.331699 10.330026 7.592854

H 11.138661 11.337180 8.378018

O 0.305694 3.504484 4.553342

H 0.241062 3.481165 3.577757

H 0.283154 4.484233 4.753228

O 7.499165 5.700379 1.850995

H 7.606968 5.821503 2.877474

H 7.930794 4.826910 1.682244

O 10.480655 1.735027 4.887589

H 10.079758 1.251805 4.115783

H 10.178547 1.213352 5.683788

O 7.478598 0.437221 4.945561

H 7.291658 1.381855 4.649187

H 6.581921 0.172894 5.275840

XYZ Coordinates from $Ir_3(O\mu_2)_4(OH\mu_1)_5(H_2O)_5^{2+}$

Ir 4.620135 6.656881 5.847143

O 5.499504 4.988917 5.337506

Ir 7.287840 4.510057 5.447636

O 7.746659 6.010484 4.236456

Ir 8.001257 7.449933 5.564134

O 7.000857 3.106557 6.815495

H 6.038732 3.516696 7.994158

O 9.334555 4.104439 5.559023

H 9.828776 3.298712 5.107093

O 2.929600 5.414682 6.066208

H 2.148497 5.738596 5.446435

O 3.423483 8.200171 6.362973

H 3.796890 8.812239 7.065802

O 8.289215 8.748854 7.174577

H 7.489312 9.394831 7.409864

O 8.111268 9.096617 4.273957

H 6.577319 2.291118 6.421631

H 3.103764 4.444265 5.742146

H 8.460977 8.114471 7.996416

H 7.480408 9.867822 4.475323

O 6.092615 7.806836 5.638676

O 7.676983 5.949473 6.684172

H 8.204065 8.840262 3.260730

O 9.893132 7.360392 5.433398

H 10.423959 7.230749 6.337141

O 6.978842 2.989815 4.092071

H 7.537241 3.195044 3.275691

O 4.966127 6.217129 7.718497

H 4.620975 6.826455 8.450798

O 3.923346 6.876696 4.075120

H 4.298940 6.174835 3.453575

H 5.648027 2.894125 3.670721

O 11.141959 6.867537 7.584710

H 11.700759 7.642159 7.868460

H 10.382626 6.890567 8.239248

O 8.444892 8.764652 11.047251

H 9.100607 8.894285 11.760039

H 8.383531 9.680564 10.537192

O 4.194577 9.659018 8.477135

H 4.183377 8.902046 9.157186

H 3.530910 10.312155 8.773357

O 6.482457 10.932552 1.508295

H 6.911880 10.502682 0.708160

H 7.235463 11.156603 2.090311

O 2.024206 9.494844 4.456598

H 2.672089 10.202384 4.200269

H 2.553178 8.985213 5.140131

O 8.399068 10.962587 9.747211

H 7.639880 10.956851 9.107493

H 9.186967 10.881115 9.114728

O 5.153721 8.970881 2.528035

H 5.805458 9.645623 2.108317

H 5.649899 8.465210 3.204633

O 5.992370 7.211813 -0.440106

H 6.411044 6.702356 0.297835

H 5.053275 7.306451 -0.147918

O 0.914236 7.616971 6.957998

H 1.886060 7.880676 6.805861

H 0.443549 8.458679 7.113828

O 8.664300 3.395928 2.038834

H 9.433829 3.823074 2.468843

H 8.909486 2.423606 2.062306

O 9.379237 0.901780 2.751303

H 8.757865 0.524142 3.440103

H 9.745032 0.138089 2.264052

O 4.622134 2.861882 3.306592

H 3.993951 2.889109 4.126577

H 4.501411 3.758028 2.805046

O 9.642655 1.185644 6.964327

H 9.875716 0.333729 7.382043

H 8.903684 0.963113 6.314879

O 4.637360 5.149097 2.180950

H 5.606077 5.332189 1.980544

H 4.135210 5.855790 1.636212

O 9.725861 3.692848 8.062759

H 9.551176 4.044142 6.576365

H 9.520667 2.730449 7.904236

O 5.492204 0.830523 6.183853

H 4.830272 1.088747 6.889264

H 4.954441 0.862703 5.367871

O 6.567520 10.498977 7.710147

H 5.648789 10.203802 8.011977

H 6.468341 10.870466 6.783775

O 7.510867 9.322612 -0.285651

H 8.011755 9.422710 -1.117692

H 6.856413 8.546213 -0.426296

O 6.571443 11.096298 5.074081

H 6.979082 11.957899 4.850804

H 5.645985 11.129598 4.650312

O 5.353368 3.862466 8.669216

H 5.137967 4.780735 8.278480

H 6.809452 4.595547 9.435026

O 0.861591 6.102641 4.761087

H 0.626815 6.755360 5.489839

H 1.063229 6.656009 3.934791

O 4.219157 11.016772 3.862572

H 4.176996 11.730085 3.192932

H 4.484963 10.193633 3.323932

O 6.577785 6.918318 11.363104

H 7.231544 7.689413 11.356257

H 6.325042 6.808738 12.301291

O 7.663316 4.953044 9.805116

H 8.997930 4.039786 8.632877

H 7.345867 5.510482 10.569361

O 8.922168 7.173766 9.030944

H 8.871670 7.714154 9.887316

H 8.354833 6.344515 9.155651

O 4.361919 7.523567 9.931283

H 5.208134 7.366558 10.451994

H 3.775190 6.721931 10.194933

O 3.102742 5.309335 10.429732

H 2.428365 5.305550 9.666396

H 3.790885 4.681386 10.124024

O 1.447883 5.340343 8.385299

H 1.112500 6.239726 8.125556

H 2.069279 5.177334 7.630473

O 3.662763 2.081398 7.695831

H 4.213689 2.800619 8.165629

H 3.029635 1.739942 8.357042

O 1.436044 7.694268 2.684818

H 0.610112 8.027740 2.279819

H 1.730029 8.453560 3.324438

O 3.617294 7.289330 1.098158

H 4.202323 7.915381 1.623850

H 2.728012 7.422687 1.545636

O 3.001668 2.986133 5.303723

H 2.004217 3.000825 5.058853

H 3.129335 2.556101 6.210723

O 12.439532 9.091511 8.513973

H 12.651252 9.020715 9.467231

H 13.283449 9.361190 8.097313

O 8.619663 8.276270 1.934214

H 8.318465 8.754870 1.106001

H 8.235567 7.373357 1.802918

O 10.230826 10.611104 7.843038

H 9.665454 9.907111 7.434941

H 11.042828 10.115780 8.126511

O 0.525722 3.380464 4.590943

H 0.510756 3.223240 3.625516

H 0.492352 4.376526 4.656162

O 7.213226 5.804085 1.746056

H 7.383874 5.935362 2.754025

H 7.706815 4.968288 1.548037

O 10.777121 2.192232 4.724280

H 10.384243 1.664337 3.974388

H 10.583476 1.649865 5.539774

O 7.920656 0.665144 4.963127

H 7.558211 1.564628 4.659210

H 7.119631 0.256860 5.369036

XYZ Coordinates from $Ir_3(O\mu_2)_4(OH\mu_1)_6(H_2O)_4^+$

Ir 4.668473 6.637854 5.811470

O 5.624688 4.977855 5.413539

Ir 7.432159 4.576796 5.452353

O 7.851660 6.098792 4.243980

Ir 8.037400 7.538852 5.553781

O 7.117259 3.108488 6.774720

H 6.113512 3.538733 7.875219

O 9.514054 4.235705 5.678518

H 9.949271 3.415057 5.209110

O 2.957956 5.431534 6.115489

H 2.183843 5.792612 5.520689

O 3.470719 8.199146 6.287072

H 3.834060 8.789544 7.011106

O 8.215245 8.838213 7.183427

H 7.403114 9.459383 7.416282

O 8.221023 9.208711 4.274404

H 6.616906 2.376151 6.297093

H 2.967874 4.415486 5.842829

H 8.400017 8.193842 7.994402

H 7.544847 9.942063 4.459188

O 6.126414 7.794919 5.554831

O 7.803923 5.996000 6.693335

H 8.289402 8.932230 3.272907

O 9.952675 7.523440 5.542965

H 10.407153 7.085027 6.364365

O 7.488685 3.140045 4.108601

H 7.892116 3.444852 3.244622

O 5.059891 6.287438 7.706033

H 4.618243 6.848048 8.414238

O 3.964483 6.733143 4.036700

H 4.370012 6.004879 3.456781

O 11.095371 6.613991 7.678616

H 11.668126 7.390586 7.918435

H 10.310135 6.755627 8.284326

O 8.376089 8.801214 11.060217

H 9.031101 8.937138 11.772453

H 8.316570 9.711017 10.543791

O 4.121282 9.612361 8.483044

H 4.102509 8.847496 9.153975

H 3.400490 10.222011 8.734750

O 6.421458 10.829401 1.507361

H 6.841898 10.410739 0.695091

H 7.180355 11.167445 2.023102

O 2.086149 9.490308 4.366069

H 2.724386 10.190871 4.071411

H 2.621677 9.003045 5.061211

O 8.332437 10.990162 9.736263

H 7.575604 10.984703 9.094095

H 9.127627 10.909692 9.113777

O 5.290772 8.785369 2.630142

H 5.905143 9.471495 2.189074

H 5.782289 8.372842 3.371907

O 6.025181 7.102157 -0.378390

H 6.462944 6.619357 0.370550

H 5.088688 7.188170 -0.066895

O 0.990983 7.757263 7.013228

H 1.971563 7.957162 6.806483

H 0.578434 8.629453 7.165146

O 8.948851 3.593095 1.854895

H 9.755130 3.969635 2.263617

H 9.096001 2.604103 1.934397

O 9.456050 1.065852 2.637627

H 8.802025 0.645327 3.270530

H 9.882146 0.328072 2.159470

O 4.211186 2.402252 3.546270

H 3.479077 2.659315 4.171292

H 4.466988 3.258059 3.118690

O 9.542814 1.081590 6.901166

H 9.833296 0.224855 7.269931

H 8.843759 0.845392 6.208440

O 4.729975 4.904931 2.279037

H 5.684214 5.117386 2.071295

H 4.242365 5.579956 1.708896

O 9.692631 3.542005 8.163768

H 9.650207 4.066124 6.693240

H 9.384127 2.635154 7.894320

O 5.500375 1.247231 5.599544

H 4.828372 1.318271 6.329340

H 5.090316 1.712455 4.798399

O 6.457117 10.560468 7.723233

H 5.550745 10.232929 8.025077

H 6.356587 10.905866 6.787378

O 7.477993 9.249797 -0.288581

H 7.939234 9.362926 -1.141538

H 6.850474 8.440716 -0.396851

O 6.545209 11.121603 5.074854

H 6.932360 12.000066 4.883326

H 5.651081 11.125609 4.587879

O 5.402483 3.874012 8.542952

H 5.203198 4.807245 8.179617

H 6.792297 4.601352 9.384709

O 0.866884 6.217733 4.855964

H 0.687950 6.925178 5.548543

H 1.063222 6.692458 3.983886

O 4.289452 10.963364 3.695189

H 4.372024 11.576241 2.934872

H 4.570217 10.072335 3.291271

O 6.527391 6.923543 11.364499

H 7.167819 7.704278 11.356637

H 6.283466 6.808604 12.304360

O 7.629726 4.979148 9.777296

H 8.951760 3.939953 8.680367

H 7.290529 5.513480 10.547254

O 8.857082 7.234397 9.022343

H 8.810055 7.754530 9.889254

H 8.296323 6.399566 9.135183

O 4.272937 7.489487 9.965475

H 5.132192 7.356324 10.468398

H 3.756769 6.626092 10.160313

O 3.167519 5.152871 10.312999

H 2.441811 5.230655 9.605150

H 3.842403 4.581462 9.887193

O 1.396371 5.416971 8.379381

H 1.117342 6.346329 8.164067

H 2.019257 5.257605 7.623575

O 3.764193 1.985080 7.604611

H 4.299450 2.728957 8.043411

H 3.417257 1.437372 8.335049

O 1.482461 7.631377 2.654032

H 0.676827 7.965477 2.210481

H 1.776459 8.411814 3.263817

O 3.729509 7.131940 1.162711

H 4.316980 7.710412 1.733335

H 2.830177 7.289518 1.575741

O 2.527465 3.031758 5.543039

H 1.515707 3.152167 5.513863

H 2.795880 2.556520 6.389505

O 12.307152 8.947334 8.506280

H 12.506489 8.911768 9.464370

H 13.149798 9.236898 8.099954

O 8.683824 8.309139 1.929672

H 8.357324 8.755431 1.093364

H 8.292984 7.405016 1.838963

O 10.198037 10.633245 7.858930

H 9.590798 9.977068 7.429888

H 10.969243 10.071077 8.134111

O -0.012846 3.677367 5.304383

H -0.280201 3.324945 4.432203

H 0.164992 4.642324 5.111620

O 7.275439 5.795662 1.764824

H 7.433292 5.977365 2.762011

H 7.856319 5.008995 1.587814

O 10.798643 2.209930 4.775813

H 10.396072 1.762119 3.982109

H 10.517645 1.645777 5.551767

O 7.954818 0.674326 4.797824

H 7.827587 1.659006 4.561789

H 7.019484 0.466268 5.066872

XYZ Coordinates from $Ir_3(O\mu_2)_4(OH\mu_1)_7(H_2O)_3$

Ir 3.411435 8.035786 7.483440

O 4.243238 6.634356 6.462565

Ir 6.019746 6.177117 6.099751

O 6.355408 8.022866 5.538208

Ir 6.782576 8.889660 7.267975

O 5.607388 4.268790 6.941273

H 4.988204 4.341549 7.803213

O 7.789804 5.497942 5.598460

O 1.749629 6.950551 7.196193

H 0.926633 7.538397 7.248652

O 2.429198 9.290083 8.664403

H 3.067360 9.718539 9.298843

O 7.357803 9.664272 8.997857

O 6.733997 10.909397 6.660414

H 5.059922 3.728735 6.233185

H 1.678896 5.425709 7.597179

H 7.773105 8.996170 9.626655

H 6.404975 11.563424 7.389706

O 4.959796 9.141791 7.667902

O 6.656138 7.007400 7.733016

H 7.708240 11.211813 6.539122

O 8.682041 8.857762 6.693176

H 9.345070 8.842813 7.491750

O 5.377053 5.455212 4.263843

H 6.053504 5.845271 3.613803

O 3.874200 7.023199 9.106126

H 3.319461 7.258438 9.914078

O 2.763204 9.060514 5.917106

H 3.431292 9.236371 5.223941

H 4.290138 6.134575 3.736822

O 10.335150 8.930475 8.689158

H 10.410318 9.920532 8.784025

H 9.799651 8.643336 9.480594

O 7.407348 9.474233 12.688583

H 7.523713 9.448641 13.657895

H 7.039987 10.412566 12.469209

O 1.656957 9.640444 11.795945

H 2.070320 8.719027 11.853081

H 1.435942 9.866527 12.720239

O 5.946772 13.624751 5.679151

H 6.662402 13.236370 5.077847

H 5.716191 14.486907 5.281628

O 0.874349 10.738017 6.339510

H 1.170321 11.553302 6.789282

H 1.718104 10.168495 6.239458

O 6.331552 11.720640 11.966525

H 5.509677 11.401862 11.510575

H 6.954756 11.960630 11.223264

O 3.882243 11.912167 6.183866

H 4.618037 12.566878 5.970561

H 4.371727 11.113596 6.479255

O 7.504097 10.327162 3.032618

H 8.100522 9.608969 3.441252

H 6.599889 9.932064 3.087181

O 0.346224 8.189437 9.824307

H 1.188942 8.563430 9.387658

H 0.366302 8.703162 10.670694

O 6.950595 6.886825 2.705828

H 7.115518 6.314710 1.876862

H 6.320967 7.600323 2.462790

O 8.166994 3.993153 3.248134

H 8.049510 4.681252 3.955138

H 7.584376 3.247989 3.561062

O 3.532574 6.757642 3.258508

H 2.653484 6.675310 3.871218

H 3.967912 7.680552 3.311493

O 6.706901 1.972315 4.396613

H 6.713805 1.052318 4.069812

H 5.745491 2.202836 4.563153

O 5.029432 8.908105 3.430226

H 5.491050 8.685832 4.303877

H 4.443228 9.743100 3.553046

O 7.996018 3.033892 6.559294

H 7.932630 4.543533 5.943709

H 7.625787 2.500814 5.799989

O 1.852663 3.625402 5.311790

H 1.669393 3.754808 6.282209

H 1.655673 4.529223 4.942421

O 3.980527 10.616112 10.619443

H 3.147519 10.470126 11.154368

H 3.789476 11.399265 9.983491

O 7.923166 12.520160 4.242126

H 8.328055 13.101025 3.568215

H 7.683217 11.635882 3.733708

O 6.149050 12.873557 8.223670

H 6.971267 12.787106 8.819171

H 6.313551 13.455440 7.432821

O 4.056609 4.479795 8.934821

H 4.062758 5.501622 9.037990

H 6.092209 7.036300 10.686583

O -0.420075 8.553137 7.259947

H -0.468494 8.500153 8.253324

H -0.013568 9.450129 7.078660

O 3.598860 12.599837 8.955129

H 4.557984 12.852319 8.822500

H 3.391431 12.281492 8.041380

O 5.325219 8.355260 11.503166

H 6.048725 8.719775 12.101616

H 4.995705 9.144820 10.997349

O 6.740520 6.358905 10.339277

H 7.163451 3.271017 7.039273

H 6.655815 6.485685 9.354177

O 8.688234 8.092132 10.718770

H 8.366906 8.601296 11.514768

H 8.026921 7.320474 10.623343

O 2.924919 7.355638 11.598356

H 3.876108 7.618578 11.783951

H 2.865126 6.344795 11.681898

O 2.655685 4.738952 11.458017

H 1.781831 4.911275 11.002193

H 3.251838 4.508371 10.701505

O 0.413949 5.503788 10.112299

H -0.462046 5.291491 10.489126

H 0.418359 6.513414 10.020727

O 1.612459 4.460901 7.913361

H 3.133348 4.349112 8.526666

H 1.012698 4.595303 8.699550

O 0.739792 10.272869 3.757620

H 0.193013 10.861419 3.199655

H 0.660404 10.650817 4.690347

O 3.427552 10.930924 3.682062

H 3.513538 11.375948 4.574895

H 2.469955 10.651131 3.631901

O 1.533136 6.311209 4.666071

H 0.696460 6.883876 4.495476

H 1.734640 6.547967 5.635324

O 10.334705 11.692665 8.655667

H 11.179033 12.182849 8.693081

H 10.017254 11.770992 7.706824

O 9.228234 11.460813 6.238966

H 8.952394 11.900572 5.368331

H 9.256507 10.470663 6.104487

O 7.962939 12.042943 9.828441

H 7.722873 11.104381 9.498827

H 8.927658 12.103085 9.571537

O -0.528969 7.903862 4.480589

H -0.108047 8.752592 4.165334

H -0.679143 8.067096 5.444445

O 8.925527 8.324705 4.021397

H 8.829368 8.353555 5.013065

H 8.250145 7.670208 3.700930

O 7.395721 5.031731 0.955781

H 7.702791 4.541563 1.793464

H 6.494774 4.678148 0.817068

O 4.403012 3.164672 4.984570

H 4.719803 3.980809 4.479551

H 3.387277 3.295648 5.095398

4.6 Molecular structure of initial Ir_3O_{14} configurations for protocol (iii) calculations using R2SCAN

XYZ Coordinates from $Ir_3(O\mu_2)_4(OH\mu_1)_5(H_2O)_5^+$

Ir 4.559536 6.581970 5.808708

O 5.437591 4.935199 5.291404

Ir 7.202181 4.402146 5.529864

O 7.799596 5.879809 4.404099

Ir 7.948950 7.306186 5.750618

O 6.762842 2.949812 6.806694

H 5.783087 3.363135 7.961493

O 9.192757 3.813621 5.721304

H 9.548675 2.989694 5.244795

O 2.844604 5.395086 5.839189

H 1.971196 5.757625 5.423406

O 3.408429 8.160353 6.311661

H 3.779411 8.703536 7.049429

O 8.250816 8.698649 7.276919

H 7.446087 9.280530 7.525948

O 8.180532 8.986376 4.503494

H 6.313939 2.205970 6.344744

H 2.963997 4.449444 5.479310

H 8.654695 8.268769 8.088551

H 7.438041 9.649655 4.575793

O 6.086166 7.689700 5.803670

O 7.643700 5.773596 6.837451

H 8.375517 8.713623 3.532833

O 9.863903 7.117001 5.640251

H 10.341567 7.856110 6.088952

O 6.896495 2.920735 4.065501

H 7.543299 3.053252 3.307001

O 4.746453 6.097153 7.696498

H 4.445784 6.755743 8.366963

O 4.142659 6.946737 3.899781

H 4.484139 6.223420 3.308198

H 5.887626 2.894776 3.656000

O 11.249173 8.778443 7.296608

H 12.031213 9.291076 7.597179

H 10.889753 8.346217 8.102599

O 8.426812 9.009195 11.025151

H 8.773722 9.250595 11.897021

H 8.295855 9.887799 10.502682

O 4.041474 9.551336 8.569434

H 4.054637 8.795735 9.227624

H 3.333491 10.152102 8.846131

O 6.537593 10.605289 1.836657

H 7.140601 10.272238 1.125024

H 7.126423 10.875586 2.555526

O 1.827714 9.804657 4.876463

H 2.470713 10.450195 4.512190

H 2.416231 9.210667 5.409191

O 8.178634 11.152860 9.654957

H 7.459172 11.028722 8.996558

H 8.988956 11.200198 9.072012

O 4.520755 9.016636 2.328893

H 5.425020 9.428023 2.209898

H 4.560736 8.297344 3.023785

O 6.795158 7.152578 -0.467330

H 7.097651 6.624934 0.300435

H 5.826768 7.249914 -0.302537

O 1.010904 7.573036 7.233505

H 1.941101 7.821769 6.932222

H 0.617371 8.396996 7.557718

O 8.751779 3.099038 2.183765

H 9.568324 3.384267 2.626972

H 8.846049 2.114540 2.132306

O 8.996966 0.464863 2.728924

H 8.338254 0.141553 3.388742

H 9.328697 -0.320394 2.268476

O 4.626347 2.845744 3.111474

H 3.959930 2.927794 3.839380

H 4.576360 3.704778 2.611460

O 9.178350 0.717707 7.062394

H 9.376921 -0.152094 7.440833

H 8.446340 0.553428 6.412423

O 4.878515 5.215115 1.964361

H 5.860117 5.329296 1.878869

H 4.495767 5.823935 1.282688

O 9.483477 3.236889 8.165068

H 9.353995 3.652691 6.739305

H 9.211006 2.307100 7.997567

O 4.987262 0.889838 5.870060

H 4.387208 1.107973 6.623647

H 4.451642 1.116405 5.093151

O 6.392541 10.386442 7.700784

H 5.497797 10.105498 8.038235

H 6.271629 10.662679 6.758289

O 8.063011 9.356533 0.043162

H 8.589492 9.644740 -0.716806

H 7.546318 8.534288 -0.251204

O 6.383488 10.906940 5.002161

H 6.810914 11.771458 4.885212

H 5.483817 11.009944 4.567301

O 5.102433 3.711802 8.627809

H 4.938570 4.643860 8.275261

H 6.692062 4.037477 9.523585

O 0.604810 6.218080 4.956818

H 0.483177 6.805733 5.745645

H 0.905347 6.833254 4.197988

O 4.010120 11.123881 3.812229

H 3.964297 11.910818 3.246294

H 4.084417 10.351603 3.173938

O 6.889670 6.838488 10.731021

H 7.406408 7.677938 10.873254

H 6.776075 6.482841 11.627293

O 7.596241 4.269940 9.827438

H 8.759862 3.634443 8.722877

H 7.522462 5.235053 9.985453

O 9.671896 7.628107 9.202406

H 9.345465 8.108992 10.019669

H 9.563614 6.681413 9.382904

O 4.316279 7.434944 10.053290

H 5.266233 7.265942 10.282263

H 3.857965 6.567786 10.276142

O 3.274748 5.046656 10.460220

H 2.629768 5.051554 9.695294

H 3.980490 4.454003 10.146061

O 1.636580 5.145790 8.366902

H 1.305490 6.055676 8.188030

H 2.212106 5.010800 7.587150

O 3.285602 2.112100 7.584812

H 3.889463 2.754489 8.071687

H 2.635006 1.799899 8.231228

O 1.647602 7.752570 3.165995

H 2.560116 7.414152 3.352234

H 1.645862 8.600830 3.692312

O 4.192069 7.268583 0.360182

H 4.213623 8.009367 1.033022

H 3.395344 7.412291 -0.172272

O 2.785924 2.934200 5.098052

H 1.809878 3.020896 4.873648

H 2.842315 2.591709 6.031085

O 12.901898 10.703305 8.349669

H 13.321571 10.526399 9.206001

H 13.602213 11.122199 7.825403

O 8.998437 8.147957 2.246045

H 8.785988 8.661991 1.424997

H 8.666879 7.250401 2.042120

O 10.173642 11.254659 7.894301

H 10.260186 10.340920 7.540147

H 11.082936 11.398280 8.222301

O 0.266215 3.517277 4.523173

H 0.173640 3.474854 3.558948

H 0.247275 4.485721 4.709837

O 7.565321 5.675477 1.854972

H 7.629148 5.797044 2.861606

H 7.999450 4.811841 1.697916

O 10.368771 1.697180 4.804654

H 9.988184 1.197256 4.047705

H 10.126827 1.171827 5.602542

O 7.493123 0.397275 4.961392

H 7.324919 1.334363 4.687146

H 6.602708 0.133206 5.274726

XYZ Coordinates from $Ir_3(O\mu_2)_4(OH\mu_1)_5(H_2O)_5^{2+}$

Ir 4.596448 6.655632 5.861604

O 5.486766 5.005620 5.396786

Ir 7.272156 4.541012 5.512609

O 7.691198 6.063447 4.317988

Ir 7.966327 7.463152 5.650121

O 7.007686 3.123934 6.851868

H 5.994729 3.510347 8.051363

O 9.298889 4.140458 5.588293

H 9.759136 3.337580 5.127211

O 2.911528 5.452750 5.876547

H 2.122221 5.774131 5.277917

O 3.436772 8.189534 6.339467

H 3.815522 8.798301 7.028403

O 8.271608 8.773121 7.235293

H 7.500353 9.445509 7.447237

O 8.085233 9.103900 4.363137

H 6.605279 2.319691 6.441060

H 3.088243 4.485758 5.594976

H 8.444782 8.173652 8.057265

H 7.387435 9.811945 4.523082

O 6.100843 7.803670 5.803380

O 7.696779 5.931646 6.779083

H 8.150543 8.833873 3.370111

O 9.834277 7.357321 5.525195

H 10.405371 7.165419 6.406787

O 7.011375 3.046384 4.148378

H 7.567302 3.250078 3.346084

O 4.781497 6.177005 7.737073

H 4.626693 6.855907 8.451648

O 4.133024 7.026630 4.010355

H 4.357878 6.245787 3.431789

H 5.673324 2.909709 3.698010

O 11.114519 6.842904 7.592138

H 11.719689 7.590992 7.817110

H 10.394041 6.899540 8.273282

O 8.546792 8.782956 11.161807

H 9.227659 8.934083 11.834936

H 8.479707 9.650565 10.625858

O 4.207600 9.644944 8.482292

H 4.233998 8.920102 9.172829

H 3.533673 10.277850 8.774878

O 6.735537 10.861089 2.063042

H 6.908833 10.552431 1.143816

H 7.610505 11.125074 2.383187

O 2.028445 9.455994 4.364238

H 2.615047 10.209908 4.133923

H 2.564608 8.966044 5.034187

O 8.496170 10.931926 9.724260

H 7.737938 10.971598 9.104921

H 9.272728 10.842284 9.105287

O 4.885512 8.977326 2.437106

H 5.797841 9.348824 2.446680

H 4.775484 8.310074 3.172206

O 6.054566 7.134105 -0.487866

H 6.440470 6.652354 0.271714

H 5.093187 7.093839 -0.326774

O 0.870970 7.617207 6.841599

H 1.826200 7.888787 6.729084

H 0.394827 8.438464 7.037322

O 8.738919 3.436973 2.094354

H 9.537369 3.824779 2.489260

H 8.936402 2.467283 2.081600

O 9.334478 0.873196 2.726487

H 8.739789 0.502312 3.422708

H 9.696502 0.114254 2.244779

O 4.676414 2.853055 3.315469

H 4.026563 2.862405 4.104802

H 4.540345 3.730789 2.807111

O 9.673084 1.244801 7.047107

H 9.908480 0.411085 7.481924

H 8.939321 1.008606 6.419576

O 4.649083 5.133643 2.191426

H 5.607999 5.309107 1.990626

H 4.148186 5.772531 1.592575

O 9.719155 3.772676 8.074367

H 9.531947 4.089343 6.596043

H 9.538555 2.810960 7.951052

O 5.474135 0.871727 6.161889

H 4.801323 1.113108 6.844179

H 4.951275 0.815751 5.347104

O 6.566131 10.555123 7.701626

H 5.675103 10.239012 8.022454

H 6.423599 10.880309 6.775574

O 7.264598 9.454967 -0.169041

H 7.828569 9.585866 -0.945653

H 6.730328 8.626247 -0.358862

O 6.435332 11.059168 5.041597

H 6.888215 11.883346 4.798206

H 5.550822 11.115272 4.568594

O 5.315173 3.849028 8.710220

H 5.066438 4.742096 8.318497

H 6.807266 4.606996 9.469357

O 0.845924 6.134937 4.613504

H 0.598288 6.785270 5.322479

H 1.042671 6.667061 3.787152

O 4.113992 11.128525 3.729202

H 4.084585 11.906970 3.150748

H 4.334445 10.362788 3.119139

O 6.688811 6.896519 11.462382

H 7.323746 7.662687 11.475289

H 6.411469 6.781386 12.384607

O 7.665065 4.943705 9.816922

H 8.999227 4.115407 8.643331

H 7.393799 5.473835 10.603047

O 8.935681 7.195610 9.095821

H 8.913809 7.696045 9.960619

H 8.373925 6.376253 9.195341

O 4.466892 7.541931 10.009628

H 5.312044 7.376266 10.502142

H 3.898624 6.742243 10.243500

O 3.181936 5.290290 10.403640

H 2.541955 5.306813 9.635027

H 3.853193 4.642467 10.131508

O 1.543016 5.361849 8.309114

H 1.154907 6.224765 8.045558

H 2.128934 5.193006 7.543518

O 3.595732 2.090895 7.679397

H 4.136780 2.780038 8.167287

H 2.958140 1.738756 8.319093

O 1.456867 7.692386 2.533354

H 0.642010 8.033875 2.131390

H 1.752985 8.427784 3.169310

O 3.584390 7.157491 0.906983

H 4.151179 7.854685 1.322833

H 2.717918 7.315144 1.363119

O 2.994218 2.974471 5.249757

H 2.012185 2.979685 5.007700

H 3.108109 2.575723 6.159429

O 12.615276 9.012422 8.315380

H 12.962329 8.959074 9.220530

H 13.387129 9.238838 7.771599

O 8.502849 8.309870 1.977444

H 8.138669 8.812324 1.205637

H 8.185506 7.397236 1.813333

O 10.349697 10.554847 7.816499

H 9.776415 9.872910 7.411974

H 11.168522 10.066601 8.048438

O 0.499347 3.366448 4.558417

H 0.406718 3.144416 3.618964

H 0.459465 4.350255 4.568698

O 7.235626 5.781882 1.789781

H 7.375347 5.932989 2.779647

H 7.750308 4.965157 1.609740

O 10.688674 2.210163 4.712359

H 10.320948 1.659194 3.981631

H 10.561009 1.674792 5.529635

O 7.961295 0.690092 5.022563

H 7.603447 1.574268 4.722915

H 7.168245 0.268610 5.405693

XYZ Coordinates from $Ir_3(O\mu_2)_4(OH\mu_1)_6(H_2O)_4^+$

Ir 4.665179 6.640577 5.831283

O 5.621826 4.975091 5.470039

Ir 7.416109 4.589672 5.473650

O 7.803418 6.124624 4.293679

Ir 8.019389 7.532820 5.598261

O 7.138712 3.107671 6.766746

H 6.101802 3.523264 7.899635

O 9.490562 4.256519 5.681969

H 9.900138 3.447397 5.205262

O 2.959695 5.443256 6.094572

H 2.184827 5.798957 5.520221

O 3.488908 8.197473 6.277029

H 3.862393 8.802414 6.966751

O 8.191442 8.840252 7.204996

H 7.410572 9.492943 7.403577

O 8.201825 9.193018 4.331524

H 6.640759 2.386964 6.293415

H 2.981727 4.441091 5.840044

H 8.351450 8.228838 8.023381

H 7.524734 9.919546 4.482674

O 6.120741 7.783924 5.628418

O 7.815845 5.976567 6.722957

H 8.284089 8.943758 3.340559

O 9.923502 7.521988 5.580339

H 10.368234 7.068655 6.376697

O 7.521895 3.182231 4.130232

H 7.891084 3.490230 3.264113

O 4.990120 6.273568 7.719638

H 4.548755 6.847447 8.398139

O 4.000523 6.747660 4.046194

H 4.398808 6.026762 3.477011

O 11.071857 6.591918 7.717701

H 11.655522 7.353695 7.934084

H 10.300011 6.738353 8.316521

O 8.371720 8.817736 11.153138

H 9.029442 8.962440 11.850078

H 8.336489 9.682782 10.612126

O 4.120735 9.664494 8.470931

H 4.089234 8.914948 9.132443

H 3.387373 10.257129 8.695991

O 6.523097 10.873223 1.674718

H 6.866231 10.528980 0.812147

H 7.312481 11.201900 2.131739

O 2.050241 9.427762 4.339809

H 2.649772 10.135461 4.021048

H 2.603029 8.970032 5.024300

O 8.397034 10.960428 9.699514

H 7.646694 11.000494 9.070593

H 9.183303 10.856124 9.094842

O 5.333691 8.716951 2.580355

H 5.989144 9.399336 2.256734

H 5.723580 8.266488 3.348761

O 6.035440 7.152551 -0.395941

H 6.460266 6.678670 0.351102

H 5.093443 7.189202 -0.133796

O 0.977937 7.798366 7.015580

H 1.945563 7.980578 6.819673

H 0.579881 8.668317 7.169623

O 8.964896 3.613266 1.846753

H 9.799464 3.950689 2.211089

H 9.072557 2.631329 1.902563

O 9.390858 1.037933 2.587536

H 8.768421 0.622594 3.233970

H 9.820178 0.306464 2.118816

O 4.239180 2.412681 3.518473

H 3.503685 2.657826 4.124652

H 4.493676 3.262592 3.099784

O 9.594148 1.105885 6.946043

H 9.894039 0.264800 7.322399

H 8.887731 0.858268 6.289963

O 4.790313 4.895255 2.279337

H 5.728740 5.115449 2.070507

H 4.289184 5.539604 1.706488

O 9.691485 3.596557 8.169046

H 9.633257 4.092279 6.684713

H 9.406308 2.684712 7.934943

O 5.498931 1.245506 5.592978

H 4.830900 1.320357 6.311922

H 5.103414 1.705150 4.799449

O 6.463042 10.615095 7.669862

H 5.571728 10.301134 7.986893

H 6.343361 10.936629 6.741127

O 7.433971 9.369268 -0.302585

H 7.917173 9.477457 -1.134819

H 6.839963 8.562502 -0.419972

O 6.490011 11.115994 4.991946

H 6.876801 11.984153 4.791217

H 5.614416 11.105997 4.500981

O 5.393771 3.844144 8.549454

H 5.176708 4.759985 8.198187

H 6.792890 4.573878 9.409232

O 0.850407 6.224044 4.865831

H 0.674805 6.946779 5.523625

H 1.032411 6.660012 3.985482

O 4.236572 10.924209 3.587298

H 4.363071 11.528646 2.836542

H 4.519982 10.043967 3.209821

O 6.521641 6.899939 11.383820

H 7.145213 7.674508 11.404670

H 6.260472 6.767641 12.308616

O 7.623461 4.941741 9.795538

H 8.962544 3.980664 8.696588

H 7.299649 5.470530 10.561236

O 8.809236 7.253337 9.072382

H 8.780033 7.739744 9.942656

H 8.268324 6.421047 9.167035

O 4.235426 7.532943 9.999788

H 5.089115 7.385622 10.480739

H 3.726892 6.685179 10.186381

O 3.132447 5.166499 10.326789

H 2.418991 5.235053 9.628959

H 3.799365 4.584320 9.925330

O 1.363586 5.422854 8.363353

H 1.079472 6.340168 8.155388

H 1.990042 5.264639 7.626066

O 3.756229 1.933900 7.622065

H 4.278197 2.666408 8.059753

H 3.433202 1.367870 8.339036

O 1.450168 7.565576 2.611300

H 0.653544 7.895021 2.165772

H 1.741451 8.332068 3.208759

O 3.712870 7.067750 1.131949

H 4.301178 7.654683 1.667721

H 2.826532 7.222532 1.545798

O 2.537966 3.020444 5.541132

H 1.540853 3.142635 5.531346

H 2.794307 2.539017 6.371734

O 12.445751 8.884478 8.445333

H 12.720022 8.876112 9.376587

H 13.253381 9.120478 7.960737

O 8.630423 8.391368 1.935517

H 8.304822 8.842451 1.116687

H 8.311828 7.472255 1.815120

O 10.294796 10.548747 7.838778

H 9.685378 9.918731 7.401825

H 11.066080 9.994073 8.088388

O -0.019214 3.669110 5.369848

H -0.343131 3.306736 4.531121

H 0.142107 4.620504 5.164264

O 7.338058 5.855499 1.750985

H 7.458334 6.009265 2.738125

H 7.901623 5.069383 1.571583

O 10.729689 2.221687 4.725432

H 10.339712 1.767124 3.943717

H 10.500724 1.652553 5.498194

O 7.982936 0.680052 4.832797

H 7.875805 1.649605 4.607277

H 7.058532 0.474447 5.100742

XYZ Coordinates from $Ir_3(O\mu_2)_4(OH\mu_1)_7(H_2O)_3$

Ir 3.382402 8.037222 7.432495

O 4.193029 6.639099 6.400644

Ir 5.975569 6.177335 6.071318

O 6.363258 7.990243 5.491673

Ir 6.755860 8.853538 7.229565

O 5.591193 4.279939 6.898956

H 5.000972 4.356689 7.758224

O 7.746956 5.525829 5.616324

O 1.731979 6.943155 7.262059

H 0.932042 7.538247 7.290637

O 2.414808 9.295920 8.595936

H 3.051324 9.725233 9.215100

O 7.283657 9.627683 8.965577

O 6.823225 10.846857 6.578786

H 5.063072 3.721303 6.219818

H 1.656900 5.386988 7.651374

H 7.646212 8.959354 9.602726

H 6.519646 11.504733 7.296712

O 4.923581 9.195493 7.526555

O 6.540868 7.006648 7.731113

H 7.786491 11.105811 6.405575

O 8.624760 8.719595 6.758616

H 9.280658 8.842208 7.541273

O 5.375351 5.439262 4.241482

H 6.045156 5.814301 3.597070

O 3.924998 7.108254 9.069258

H 3.351274 7.319723 9.853692

O 2.687226 9.004377 5.863629

H 3.355814 9.207534 5.187965

H 4.299041 6.095212 3.749465

O 10.254421 8.980796 8.733545

H 10.338228 9.958454 8.827633

H 9.729294 8.680838 9.513151

O 7.428184 9.494891 12.759102

H 7.527834 9.480044 13.722539

H 7.051271 10.404249 12.530304

O 1.640154 9.704558 11.786059

H 2.030832 8.789168 11.839620

H 1.441841 9.943625 12.703798

O 5.862652 13.548148 5.691384

H 6.586340 13.223452 5.089339

H 5.533970 14.361587 5.280167

O 0.893187 10.809047 6.341698

H 1.245791 11.585385 6.802763

H 1.682809 10.194611 6.230637

O 6.302111 11.718527 11.963186

H 5.497159 11.394861 11.503887

H 6.929164 11.949214 11.238620

O 3.885364 11.732443 6.354749

H 4.626156 12.354202 6.132402

H 4.320160 10.929915 6.711713

O 7.521261 10.324067 3.089480

H 8.103929 9.611663 3.487988

H 6.627482 9.929865 3.100878

O 0.343754 8.188147 9.833798

H 1.165427 8.550450 9.385980

H 0.346448 8.710956 10.661363

O 6.960020 6.888856 2.687980

H 7.149773 6.365039 1.853710

H 6.334910 7.598421 2.454624

O 8.139412 4.065345 3.244523

H 8.028698 4.743022 3.947165

H 7.591017 3.312179 3.561230

O 3.518460 6.714686 3.291318

H 2.665553 6.642494 3.903679

H 3.935900 7.633127 3.315983

O 6.741419 1.974860 4.409241

H 6.792871 1.064741 4.081347

H 5.783739 2.152802 4.573065

O 5.011027 8.873093 3.410382

H 5.486250 8.651916 4.265309

H 4.467512 9.712827 3.549779

O 8.062550 3.059630 6.548649

H 7.920646 4.592189 5.961257

H 7.677760 2.535773 5.804573

O 1.820777 3.589013 5.341253

H 1.654978 3.714611 6.303529

H 1.615085 4.477374 4.971939

O 3.971495 10.601542 10.594010

H 3.144447 10.465655 11.121710

H 3.795566 11.401937 10.001958

O 7.916897 12.603901 4.206280

H 8.246282 13.183612 3.502157

H 7.698519 11.716347 3.748026

O 6.175328 12.757877 8.201526

H 6.977492 12.725876 8.801702

H 6.304505 13.393592 7.462093

O 4.095019 4.537584 8.924945

H 4.112909 5.544289 9.020716

H 6.026515 6.989223 10.704022

O -0.427664 8.620432 7.270096

H -0.489934 8.547143 8.249124

H -0.010184 9.502623 7.111215

O 3.603287 12.653176 9.003313

H 4.559843 12.859825 8.872953

H 3.390586 12.318665 8.105478

O 5.332628 8.380862 11.532751

H 6.046287 8.724829 12.130997

H 5.018998 9.165960 11.030307

O 6.646006 6.312292 10.340568

H 7.270906 3.228772 7.093339

H 6.548953 6.453587 9.369318

O 8.614504 8.052522 10.743361

H 8.310250 8.552758 11.536241

H 7.968123 7.290742 10.649415

O 2.894758 7.399876 11.568005

H 3.830560 7.650343 11.777191

H 2.842680 6.403440 11.650616

O 2.689499 4.758483 11.450979

H 1.819734 4.900030 11.003117

H 3.287729 4.535095 10.708474

O 0.395953 5.490111 10.159173

H -0.456222 5.295948 10.577810

H 0.409206 6.482721 10.053202

O 1.611495 4.432417 7.957279

H 3.175406 4.389590 8.552932

H 1.015634 4.533914 8.735344

O 0.755024 10.359106 3.739856

H 0.222780 10.932683 3.166604

H 0.663462 10.742615 4.653002

O 3.463777 10.947168 3.766672

H 3.529027 11.303165 4.687590

H 2.510950 10.710728 3.659566

O 1.509405 6.294951 4.709187

H 0.712386 6.892730 4.532189

H 1.711200 6.510759 5.666667

O 10.376807 11.734591 8.599926

H 11.233650 12.185113 8.643822

H 10.084924 11.800034 7.658317

O 9.311877 11.491427 6.108808

H 9.016958 12.025023 5.313007

H 9.599800 10.628773 5.762117

O 8.010323 11.992377 9.843794

H 7.762103 11.079723 9.503982

H 8.954624 12.077632 9.569238

O -0.493767 7.975838 4.499749

H -0.081007 8.812144 4.178833

H -0.656034 8.146333 5.450053

O 8.942967 8.289854 4.066030

H 8.821132 8.303616 5.043207

H 8.265319 7.660627 3.733063

O 7.517726 5.089342 0.876093

H 7.770601 4.603406 1.712469

H 6.653670 4.712102 0.651543

O 4.375555 3.116556 4.962458

H 4.677009 3.911644 4.447996

H 3.379464 3.245600 5.089519
